# Supplementary material for: Trends in inequalities in Children Looked After in England between 2004 and 2019: a local area ecological analysis
Source: BMJ Open. 2020 Nov 23;10(11):e041774. doi: 10.1136/bmjopen-2020-041774 (PMC7684833; doi:10.1136/bmjopen-2020-041774)
Supplement: Supplementary data [file bmjopen-2020-041774supp001.pdf]

SUPPLEMENTARY MATERIALS

Appendix 1: Harmonising data

Where changes to LA boundaries in 2009 led to the formation of two upper tier unitary authorities from a single county, CLA numbers for preceding years were split between these LAs based on their 2009 child population ratio. In the publicly available data, for reasons of confidentiality, numbers from one to five inclusive were supressed. For each missing value we therefore imputed a random integer in this range. There were only three cases of missing data, across two years, early in the implementation of the CIN census: age stratified data were not available for Havering and Newham in 2012, or Norfolk in 2013. Given the low degree of missingness, we performed complete case analyses.

Appendix 2: Model formulae

Segmented linear regression model for age standardised CLA rate, including linear spline:

$$y_{ij} = \beta_0 + \beta_1 x_{1ij} + \beta_2 x_{2i} + \beta_3 x_{3j} + \beta_4 x_{4j} + \beta_5 x_{3j} x_{2i} + \beta_5 x_{4j} x_{2i} + U_i + V_i x_{3j} + \varepsilon_{ij}$$

Let:

- y_{ij} denote the rate of children taken into care in LA i in year j
- x_{1ij} denote covariate lagged unemployment rate, coded as a continuous variable and dependent on LA i and on year j
- x_{2i} denote the weighted rank of deprivation dependent on LA i, a continuous variable ranging from 0 to 1
- x_{3j} denote the first spline term, which is year j coded as continuous variable and centered at 2004
- x_{4j} denote the second spline term, a continuous variable that takes the value of 0 for year j≤2007, and j–2007 for year j>2007. This defines a segmented regression with knot in 2007.
- $(U_{i,}\ V_i) \sim BVN(0, S_0)$ denote random intercept and slope for LA i
- $\varepsilon_{ij} \sim N\ (0, S_1)$ denote the random error for LA i in year j

Linear regression model for age standardised CPP and CIN rates:

$$y_{ij} = \beta_0 + \beta_1 x_{1ij} + \beta_2 x_{2i} + \beta_3 x_{3j} + \beta_4 x_{3j} x_{2i} + U_i + V_i x_{3j} + \varepsilon_{ij}$$

Let:

- y_{ij} denote the rate of children taken into care in LA i in year j
- x_{1ij} denote covariate lagged unemployment rate, coded as a continuous variable and dependent on LA i and on year j

- x_{2i} denote the weighted rank of deprivation dependent on LA i, a continuous variable ranging from 0 to 1
- x_{3j} denote year j, coded as continuous variable and centered at 2004
- $(U_{i,}\ V_i) \sim BVN(0, S_0)$ denote random intercept and slope for LA i
- $\varepsilon_{ij} \sim N\ (0, S_1)$ denote the random error for LA i in year j

Appendix 3: Breakpoint analysis

In our model for age standardised CLA rates, we used an iterative search procedure in order to identify which breakpoint offered the best fit. Appendix figure 17 shows the BIC value for each successive breakpoint used in the model. This led us to fit a knot in 2007.

Appendix 4: Age stratified analyses of crude CLA, CPP and CIN rates by LA deprivation quintile

For the age stratified analyses, we calculated rates for each of our outcomes using child population data, broken down by the same age bands available in the routine and FoI data, sourced from the Office for National Statistics (ONS) mid-year population estimates, accessed via Stat-Xplore[1]. These formed our denominator. We plotted rates for all age-stratified outcomes, across years for which data were available, enabling a comparison, by age group, across outcomes. Appendix figure 1 shows that the rise in CLA rates was mainly due to children under the age of 1 and children aged 16-17 entering care. Though wide, the gap in rates between most and least deprived LAs for the youngest age group does not appear to be widening. In the oldest age group however, there is a pronounced increase in the gap from 2010.

We sought to determine whether trends in CLA were reflected through the funnel of children's social care (appendix figures 2-3). The funnel remains widest in children under the age of 1. However, the gap between most and least deprived areas is relatively stable over time regardless of the stage. In children aged 16-17, the funnel narrows considerably from CIN to CPP, then widens once more at the level of CLA. The discontinuity is unique to this age group and may relate to the CPP's focus on risks within the family home. Acute risks to older children are often in the community, from peer groups and criminal networks. This may lead children to be placed directly on a CLA when need becomes acute. The gap in rates

[1] Office for National Statistics. Population Estimates for UK, England and Wales, Scotland and Northern Ireland: Stat-Xplore; 2019 [Available from: https://stat-xplore.dwp.gov.uk].

between most and least deprived areas appears to be widening in both CIN and CLA for this older age group: trends in CLA may well be reflecting, and concentrating, trends in CIN.

Appendix 5: Crude CPP rates by deprivation quintile, stratified by category of abuse

We plotted CPP rates for all categories of abuse, enabling a comparison across categories (appendix figure 4). Neglect, then emotional abuse, are the most commonly recorded primary categories of abuse. Rates for these categories are rising. Where neglect is recorded, the gap in rates between most and least deprived areas appears to have declined slightly from 2014. In contrast, where emotional abuse is recorded, the gap increased dramatically from 2014. Further research is needed to understand how recording practices, child welfare systems, social care practices, and underlying need, may differ by area level income deprivation.

Appendix 6: predictions based on the model

We predict expected CLA rates if the rise in rates from 2007 had occurred in more deprived LAs as it did in the median LA (such that 50% of the 2008 child population live in more deprived areas):

$$y_{ij} = \beta_0 + \beta_1 x_{1ij} + \beta_2 x_{2i} + \beta_3 x_{3j} + \beta_4 x_{4j} + \beta_5 x_{3j} x_{2i} + \beta_5 x_{4j} \boldsymbol{x_5} + U_i + V_i x_{3j} + \varepsilon_{ij}$$

Where $\boldsymbol{x_5}$ denotes the weighted rank of deprivation in the median LA in the cumulative distribution. This scenario preserves the change in trend from 2007 and unemployment rates, but posits that, after controlling for unemployment rates, the change in trend should not disproportionately affect areas based on their levels of income deprivation. Appendix figure 18, showing LAs grouped by quintiles, illustrates predicted rates according to this scenario.

Appendix 7: Full model output[2]

The following tables summarise the full output for each of the models in turn:

- Age standardised CLA rates
- Age standardised CLA rates, log-transformed (results exponentiated)
- Age standardised CPP rates
- Age standardised CIN rates

[2] Hlavac M. stargazer: Well-Formatted Regression and Summary Statistics Tables. R package version 5.2.2.; 2018.

Model	**CLA, 2004-2019**	**CLA, 2004-2019**	**CPP, 2012-2019**	**CIN, 2012-2019**
Dependent variable	CLA rate (per 100,000)	Log-transformed CLA rate (per 100,000)	CPP rate (per 100,000)	CIN rate (per 100,000)
Intercept	104.31** (71.56, 137.05)	114.41 ** (101.41, 129.07)	365.74** (306.30, 425.19)	2,190.79** (1,798.70, 2,582.88)
Unemployment rate (lagged)	8.95** (6.48, 11.43)	1.04** (1.03, 1.05)	-10.41 (-22.17, 1.36)	68.52 (-3.06, 140.10)
Spline 1	3.43 (-3.41, 10.27)	1.03** (1.01, 1.06)	12.69** (3.09, 22.29)	-6.76 (-74.71, 61.19)
Deprivation	192.93** (140.01, 245.86)	2.51** (2.07, 3.05)	304.12** (198.42, 409.81)	1,637.02** (949.98, 2,324.07)
Spline 2	1.89 (-5.21, 8.99)	1.00 (0.98, 1.03)	4.38 (-11.20, 19.95)	47.08 (-62.71, 156.88)
Spline 1: deprivation	-11.38* (-22.27, -0.49)	-0.94** (-0.90, -0.98)	-	-
Spline 2: deprivation	14.86* (3.55, 26.16)	1.06** (1.01, 1.10)	-	-
Observations	2,400	2,400	1,197	1,195
Log Likelihood	-13,279.74	211.43	-7,599.82	-9,727.10
Akaike Inf. Crit.	26,581.49	-400.87	15,217.63	19,472.20
Bayesian Inf. Crit.	26,645.10	-337.25	15,263.42	19,517.98
Note:	* p < 0.05, ** p < 0.01	* p < 0.05, ** p < 0.01	* p < 0.05, ** p < 0.01	* p < 0.05, ** p < 0.01
		All coefficients are exponentiated		

Appendix 8: residual diagnostics

The residuals from our model are normally distributed. Plotting standard normal quantiles against the data results in a relatively linear pattern. When grouped by quintile, predicted and observed values of CLA rates appear relatively consistent:

a. CLA model (absolute inequalities): see appendix figures 5-7
b. CLA model (relative inequalities): see appendix figures 8-10
c. CPP model: see appendix figures 11-13
d. CIN model: see appendix figures 14-16
