# Supplementary material for: Trends in inequalities in Children Looked After in England between 2004 and 2019: a local area ecological analysis
Source: BMJ Open. 2020 Nov 23;10(11):e041774. doi: 10.1136/bmjopen-2020-041774 (PMC7684833; doi:10.1136/bmjopen-2020-041774)
Supplement: Supplementary data [file bmjopen-2020-041774supp002.pdf]

BMJ Publishing Group Limited (BMJ) disclaims all liability and responsibility arising from any reliance placed on this supplemental material which has been supplied by the author(s)

Appendix figures

Appendix figure 1: CLA rates by LA income deprivation quintile, stratified by age

Appendix figure 2: CPP rates by LA income deprivation quintile, stratified by age

Appendix figure 3: CIN rates by LA income deprivation quintile, stratified by age

Appendix figure 4: CPP rates by LA income deprivation quintile, stratified by category of abuse

Appendix figure 5: CLA model - absolute inequalities - histogram of standardised residuals

Appendix figure 6: CLA model - absolute inequalities - quantile quantile plot

Appendix figure 7: CLA model - absolute inequalities - comparing observed and predicted rates

Appendix figure 8: CLA model - relative inequalities - histogram of standardised residuals

Appendix figure 9: CLA model - relative inequalities - quantile quantile plot

Appendix figure 10: CLA model - relative inequalities - comparing observed and predicted rates

Appendix figure 11: CPP model - histogram of standardised residuals

Appendix figure 12: CPP model - quantile quantile plot

Appendix figure 13: CPP model - comparing observed and predicted rates

Appendix figure 14: CIN model - histogram of standardised residuals

Appendix figure 15: CIN model - quantile quantile plot

Appendix figure 16: CIN model - comparing observed and predicted rates

Appendix figure 17: CLA model - breakpoint analysis

Appendix figure 18: CLA model - predictions based on the model
