# Supplementary figures and images for: Trends in inequalities in Children Looked After in England between 2004 and 2019: a local area ecological analysis
Source: BMJ Open. 2020 Nov 23;10(11):e041774. doi: 10.1136/bmjopen-2020-041774 (PMC7684833; doi:10.1136/bmjopen-2020-041774)

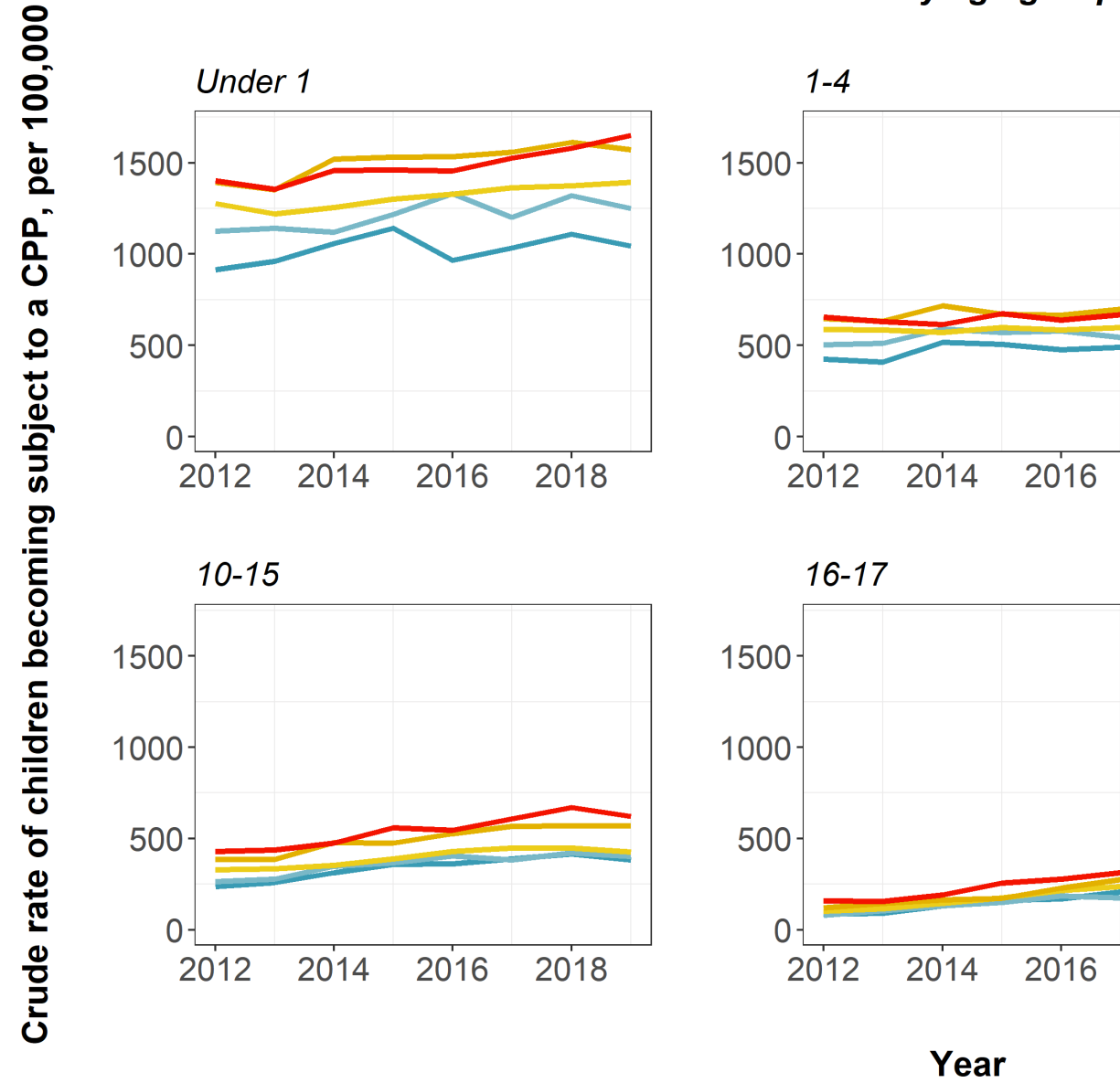

Supplement: Supplementary data [file bmjopen-2020-041774supp004.pdf]

CLA by age group

Crude rate of children entering care, per 100,000

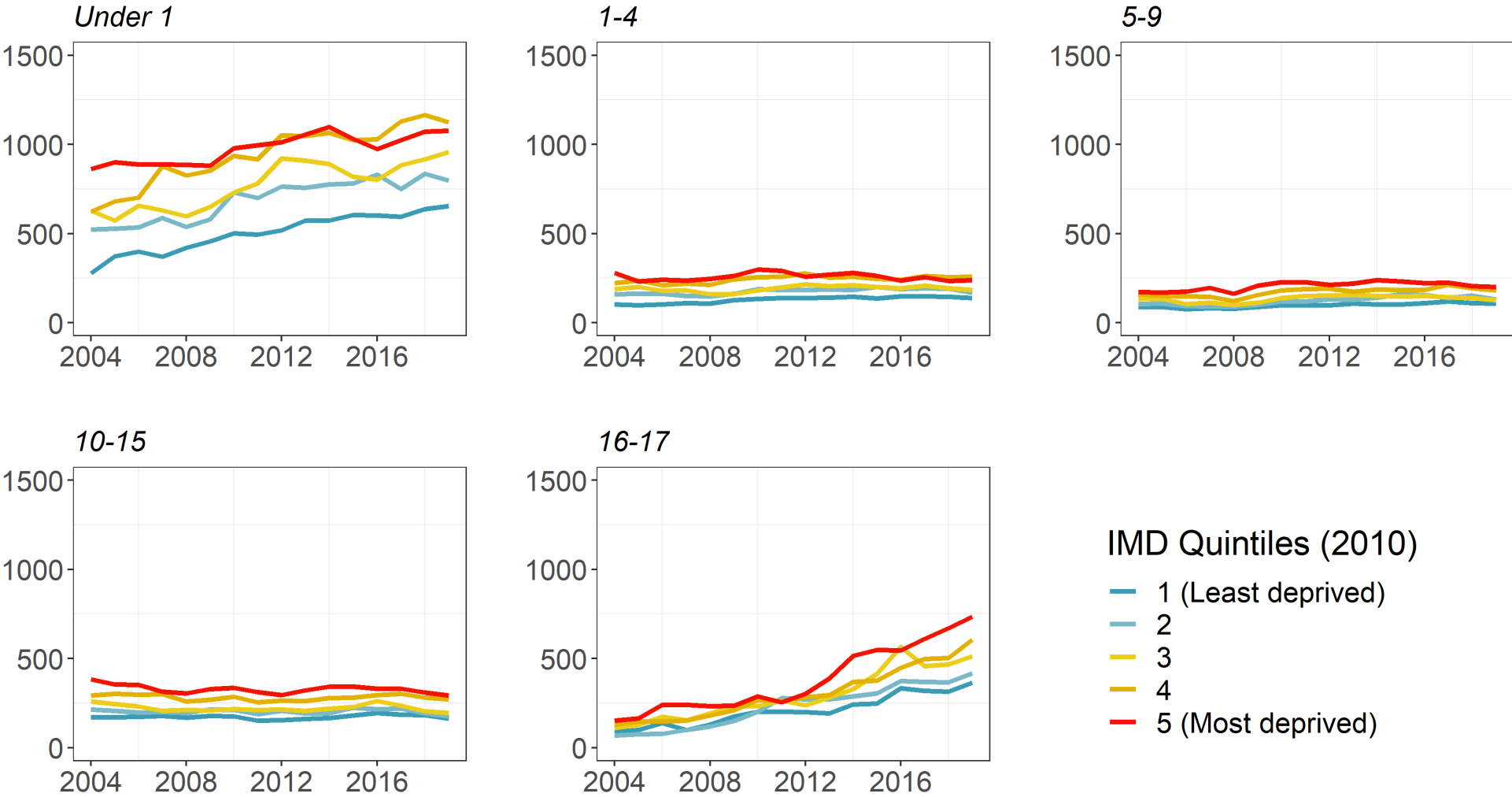

Supplement: Supplementary data [file bmjopen-2020-041774supp003.pdf]

Crude rate of children beginning an episode of need, per 100,000

CIN by age group

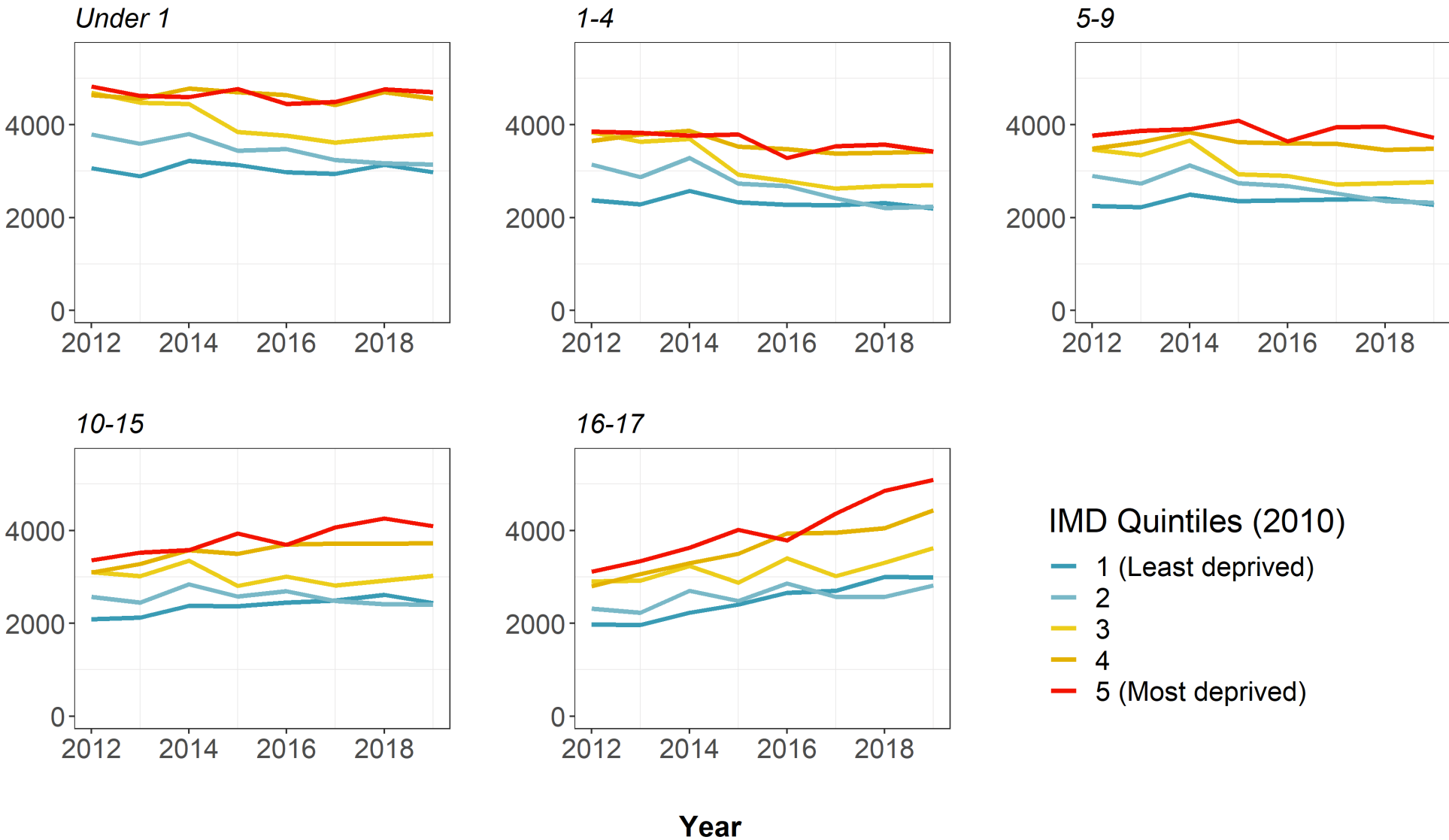

Supplement: Supplementary data [file bmjopen-2020-041774supp005.pdf]

CPP by category of abuse

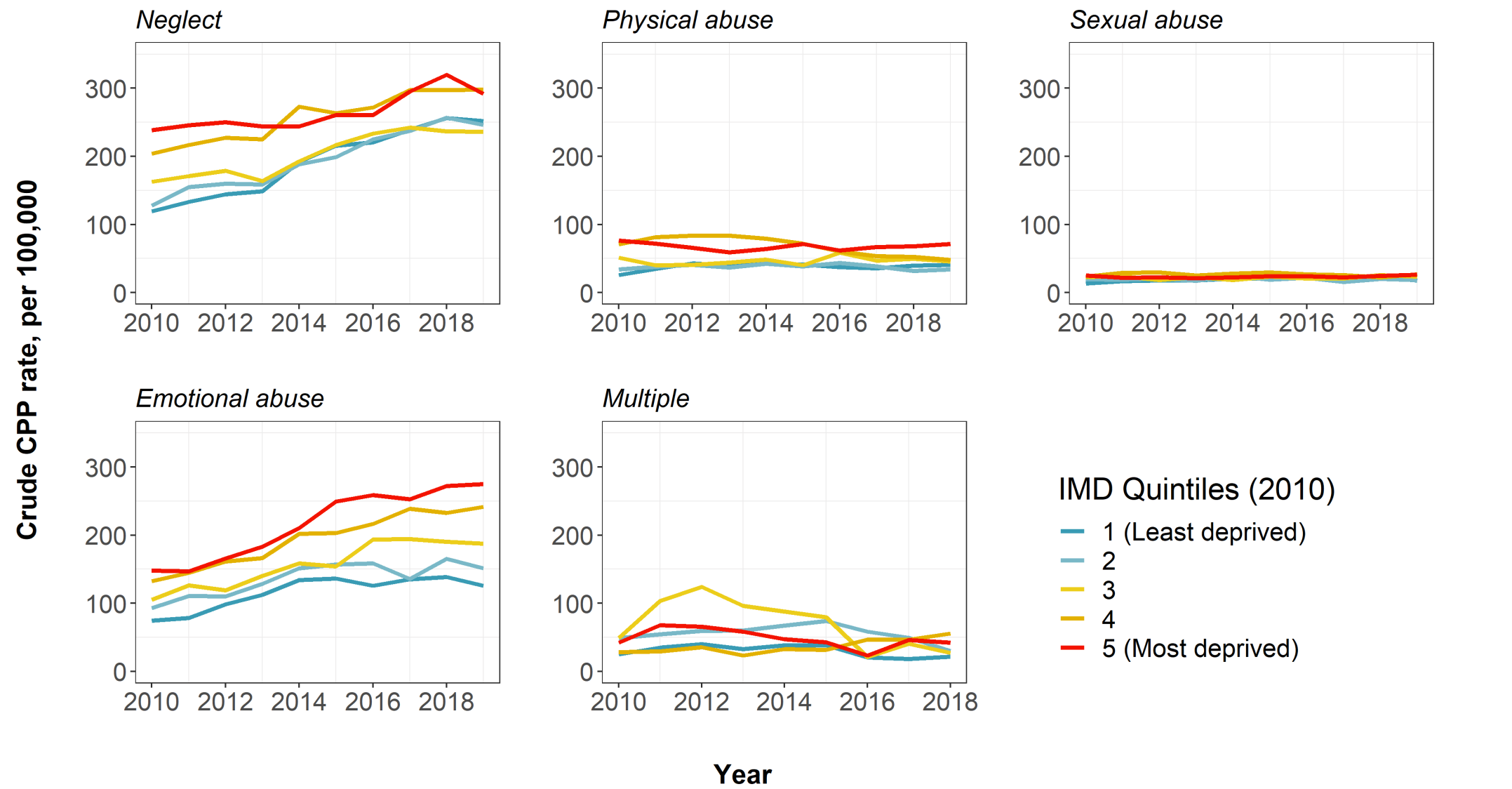

Supplement: Supplementary data [file bmjopen-2020-041774supp006.pdf]

# Histogram of stdres

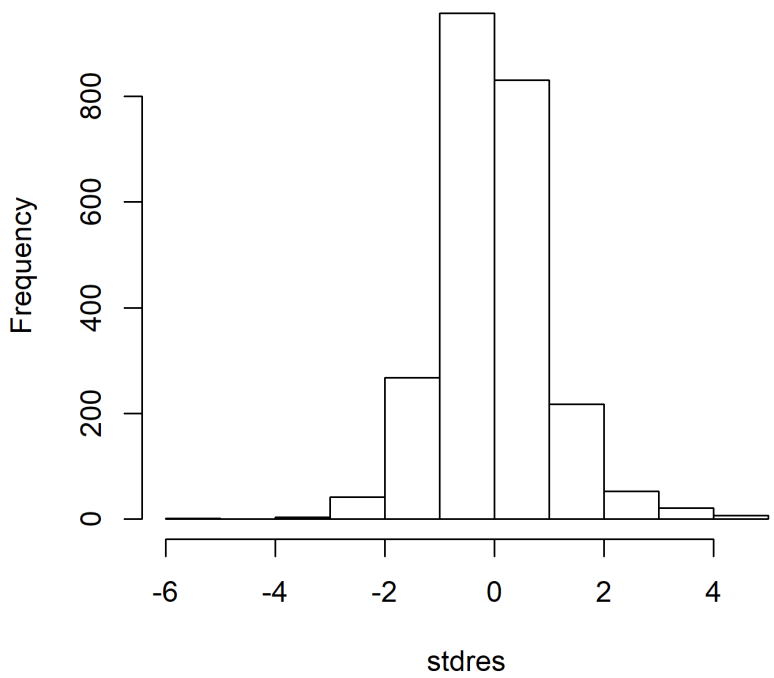

Supplement: Supplementary data [file bmjopen-2020-041774supp007.pdf]

## Normal Q-Q Plot

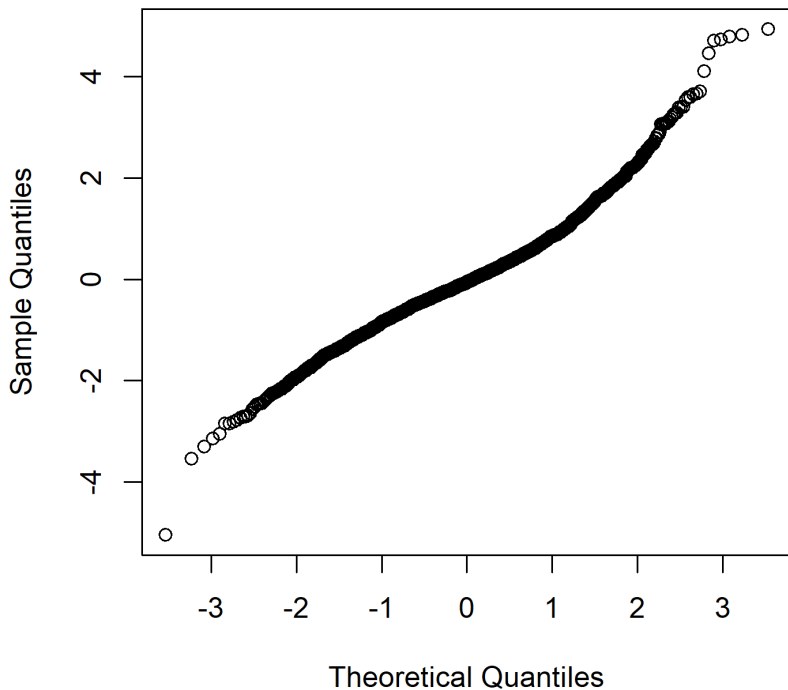

Supplement: Supplementary data [file bmjopen-2020-041774supp008.pdf]

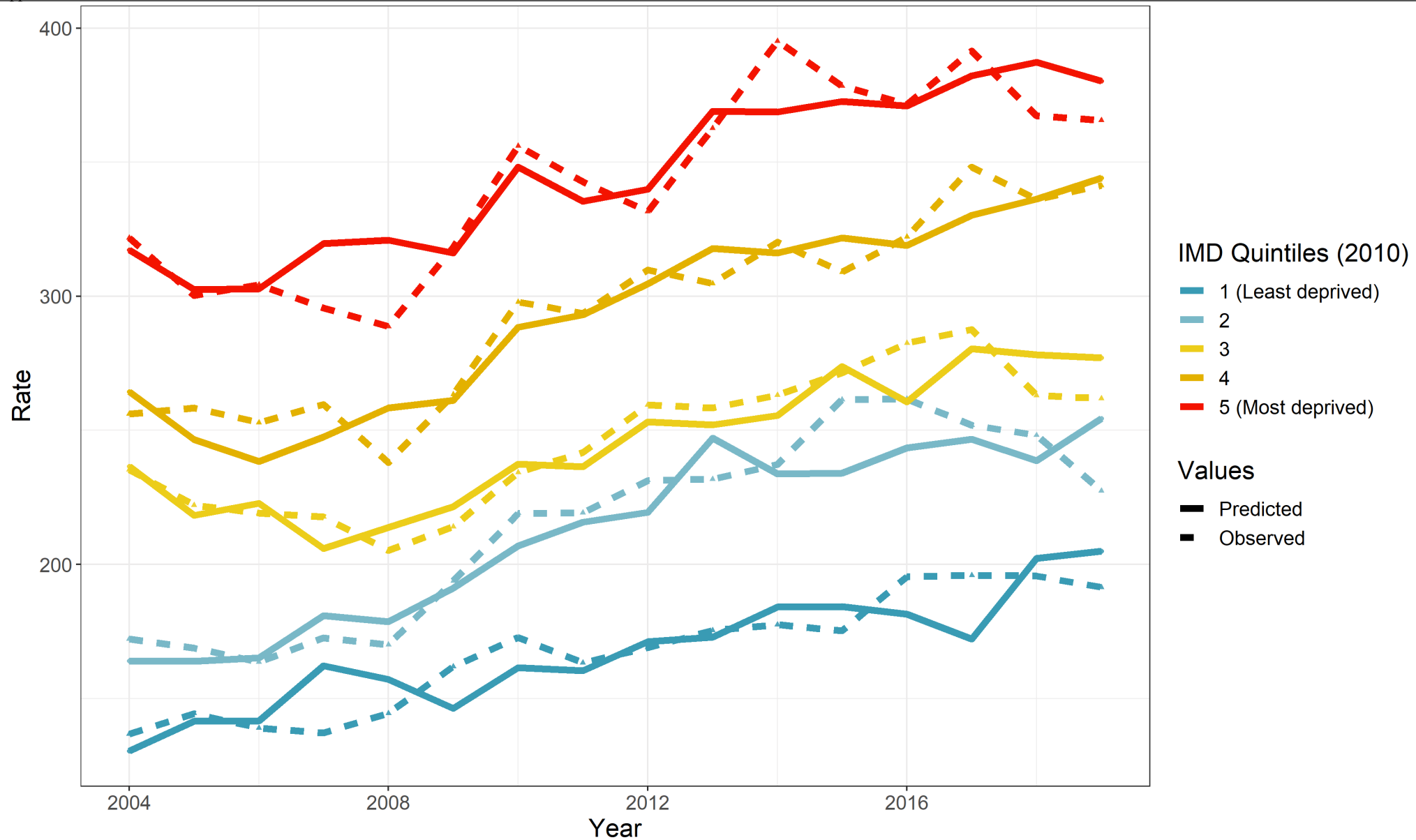

Supplement: Supplementary data [file bmjopen-2020-041774supp009.pdf]

## Histogram of stdres

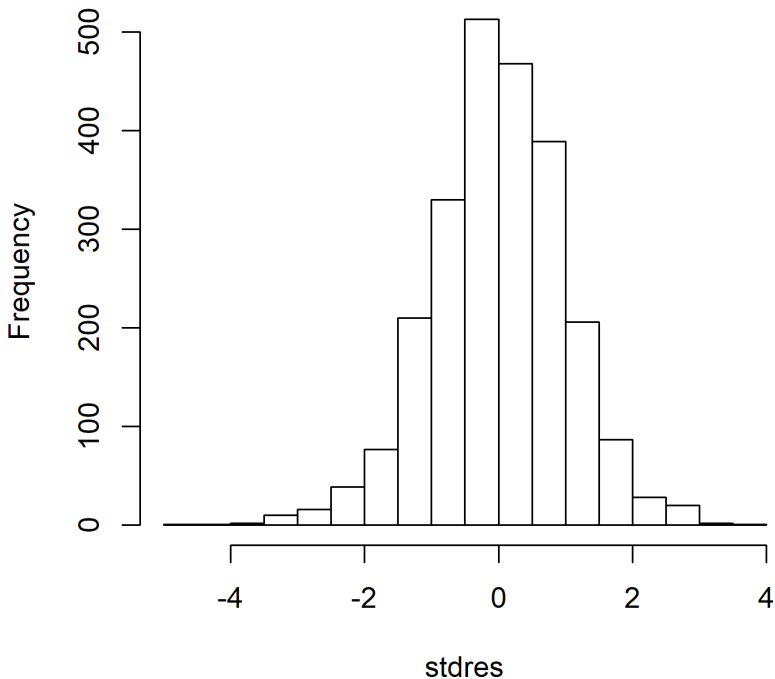

Supplement: Supplementary data [file bmjopen-2020-041774supp010.pdf]

## Normal Q-Q Plot

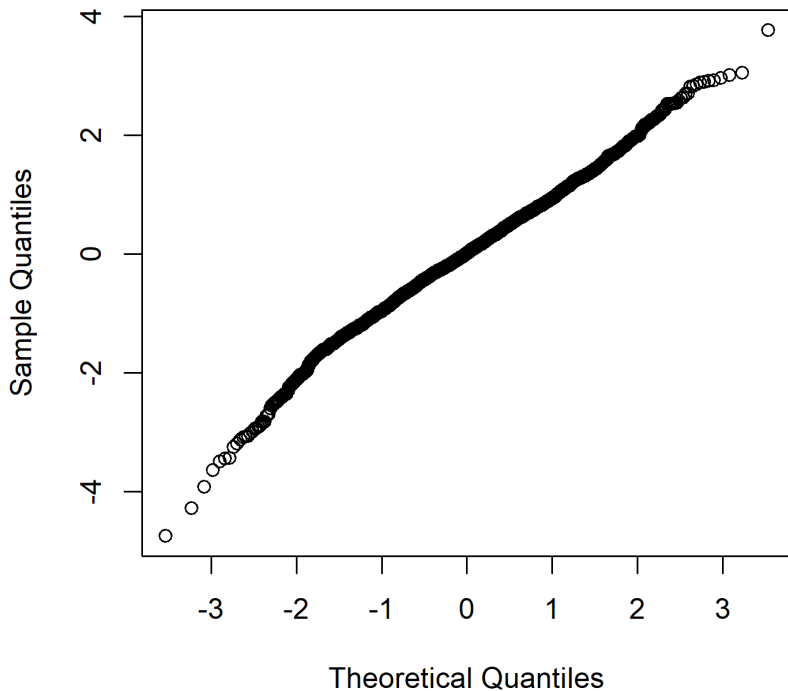

Supplement: Supplementary data [file bmjopen-2020-041774supp011.pdf]

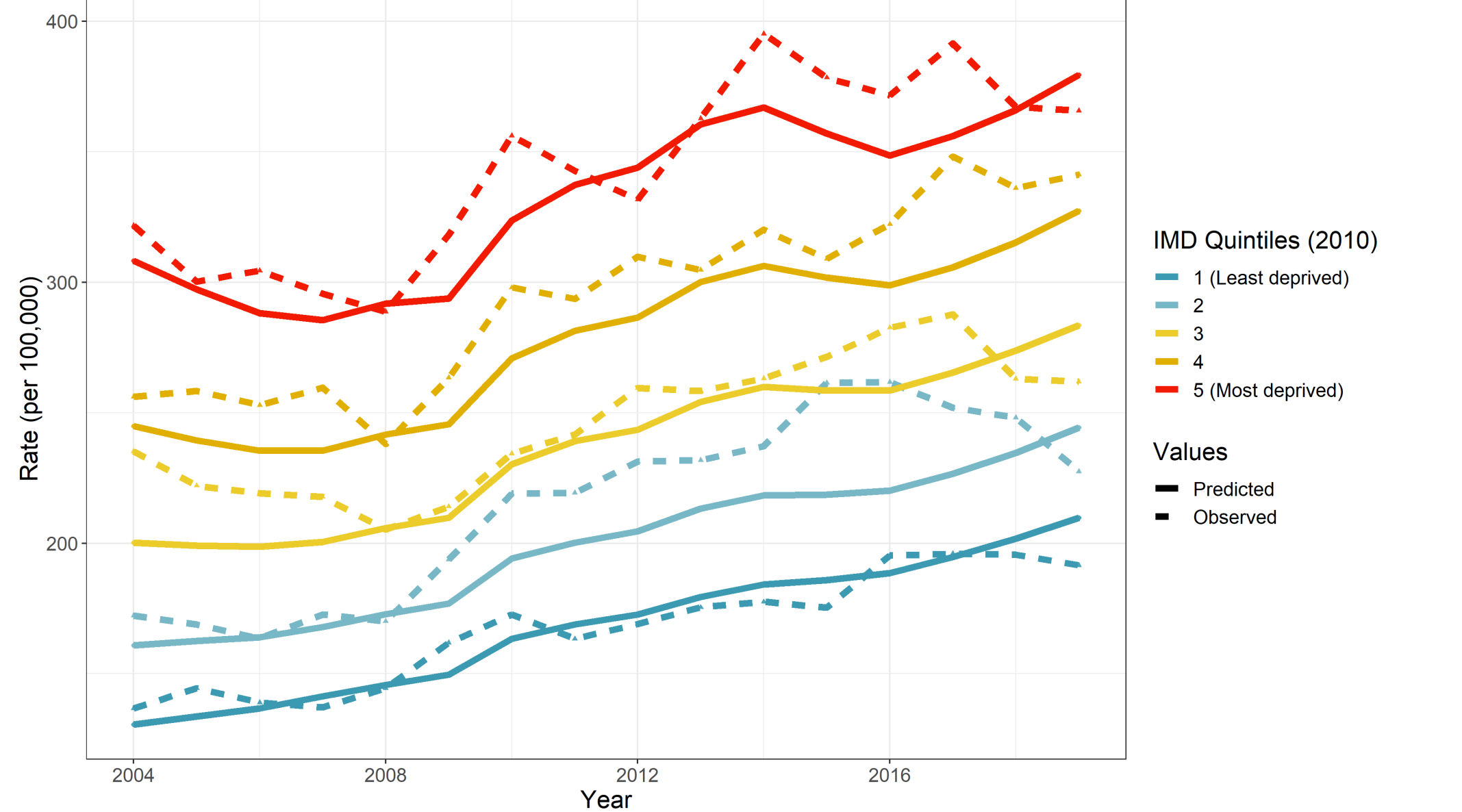

Supplement: Supplementary data [file bmjopen-2020-041774supp012.pdf]

## Histogram of stdres

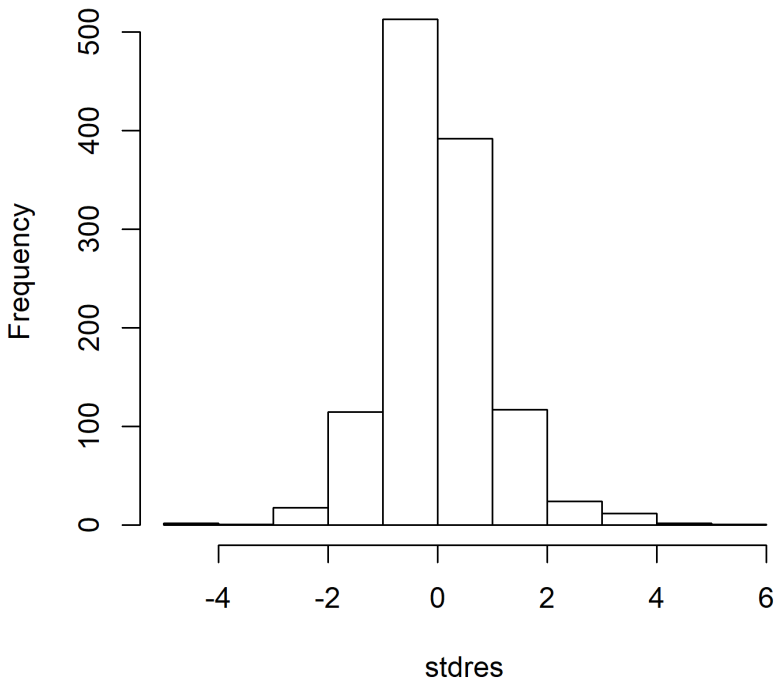

Supplement: Supplementary data [file bmjopen-2020-041774supp013.pdf]

## Normal Q-Q Plot

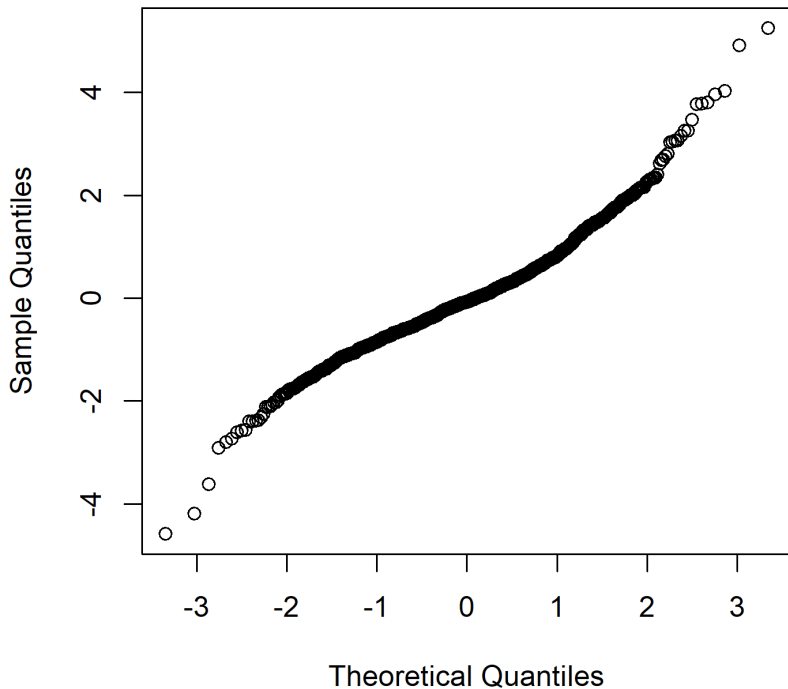

Supplement: Supplementary data [file bmjopen-2020-041774supp014.pdf]

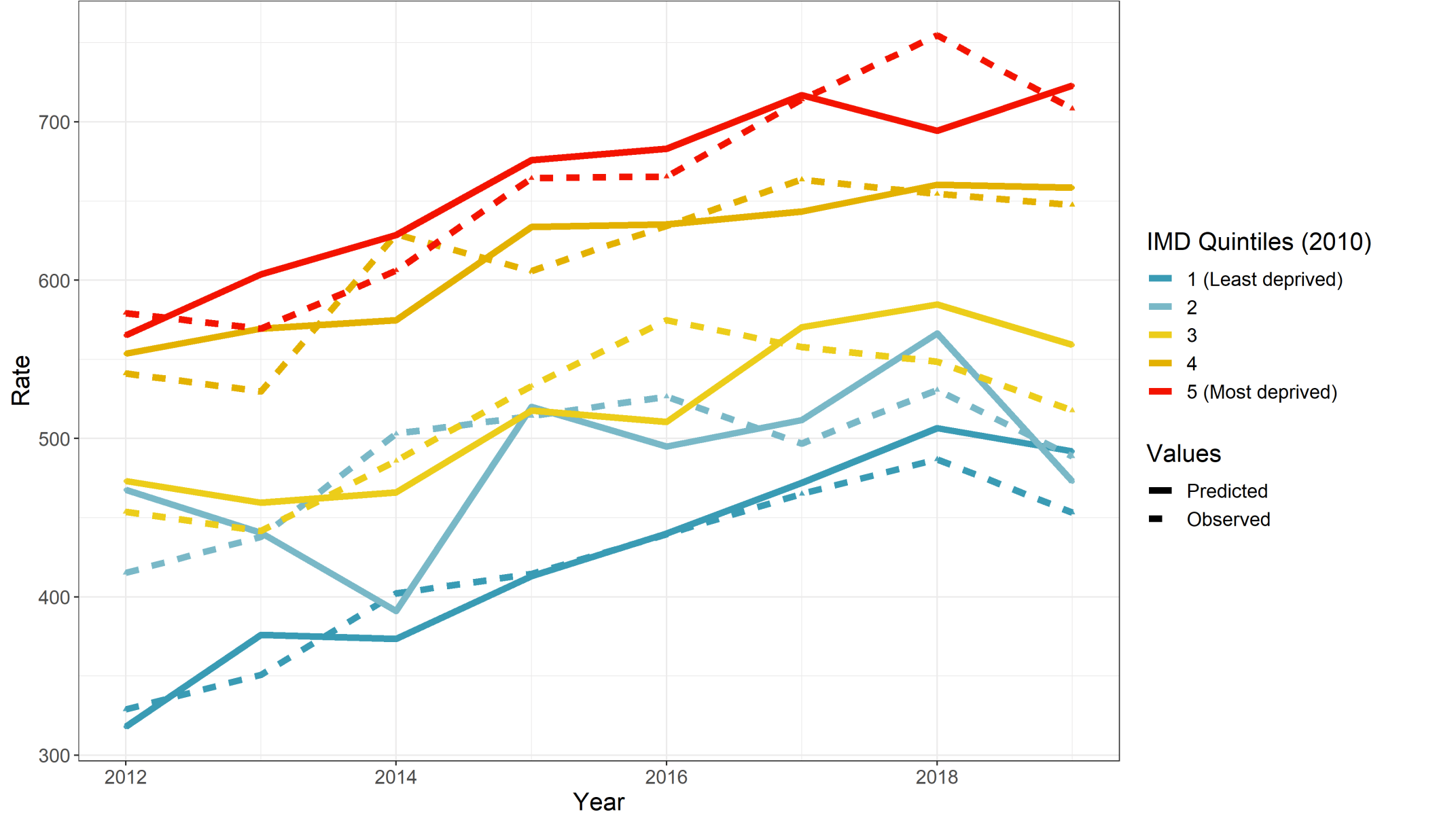

Supplement: Supplementary data [file bmjopen-2020-041774supp015.pdf]

## Histogram of stdres

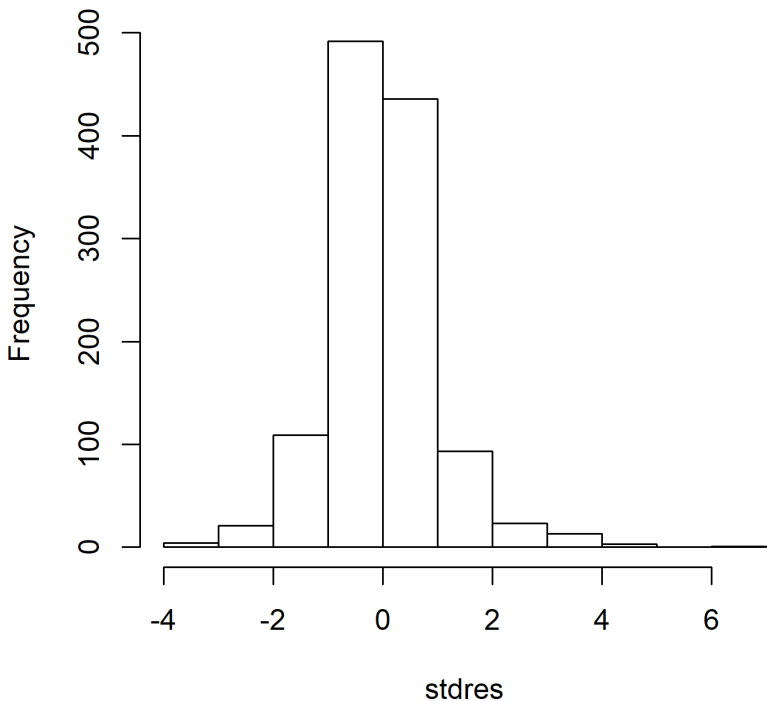

Supplement: Supplementary data [file bmjopen-2020-041774supp016.pdf]

## Normal Q-Q Plot

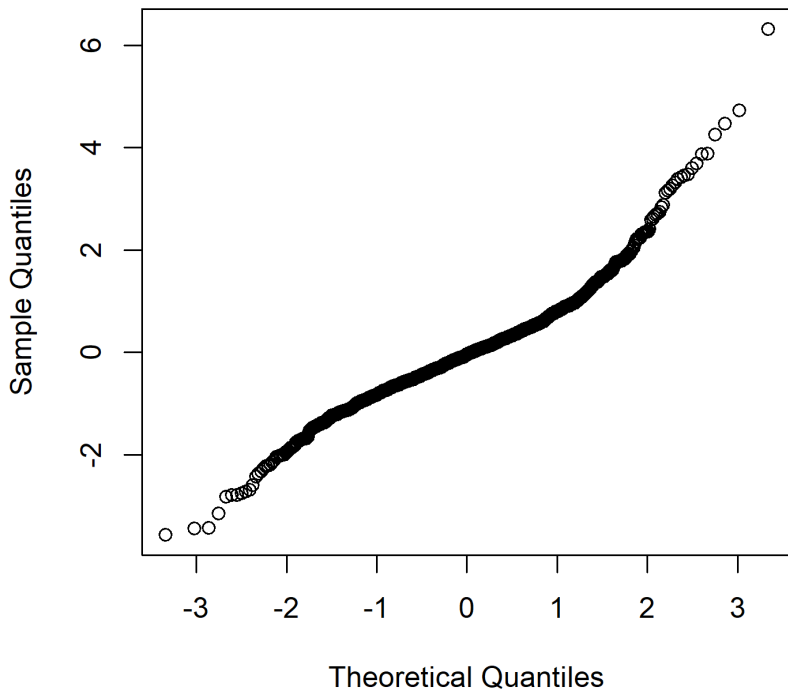

Supplement: Supplementary data [file bmjopen-2020-041774supp017.pdf]

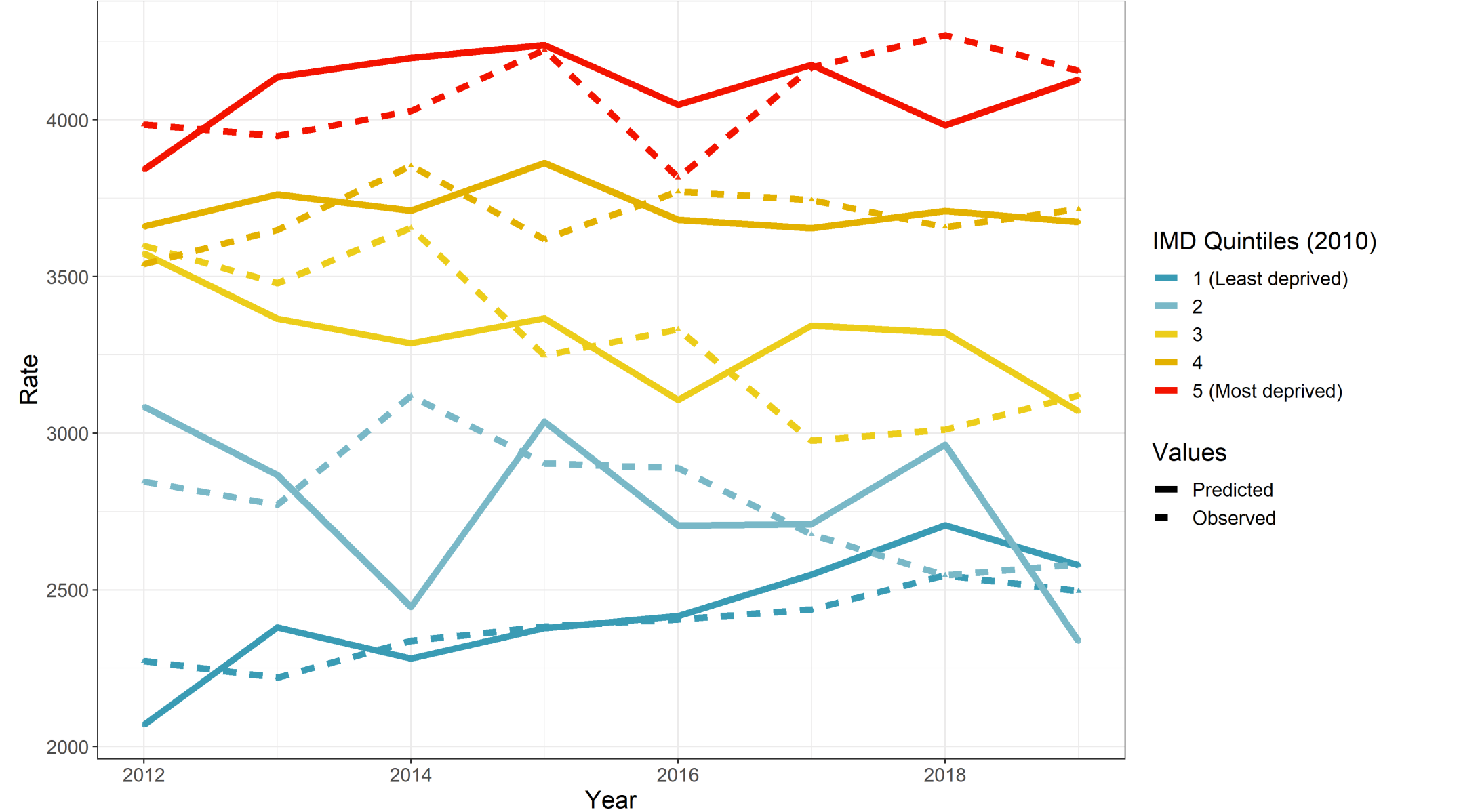

Supplement: Supplementary data [file bmjopen-2020-041774supp018.pdf]

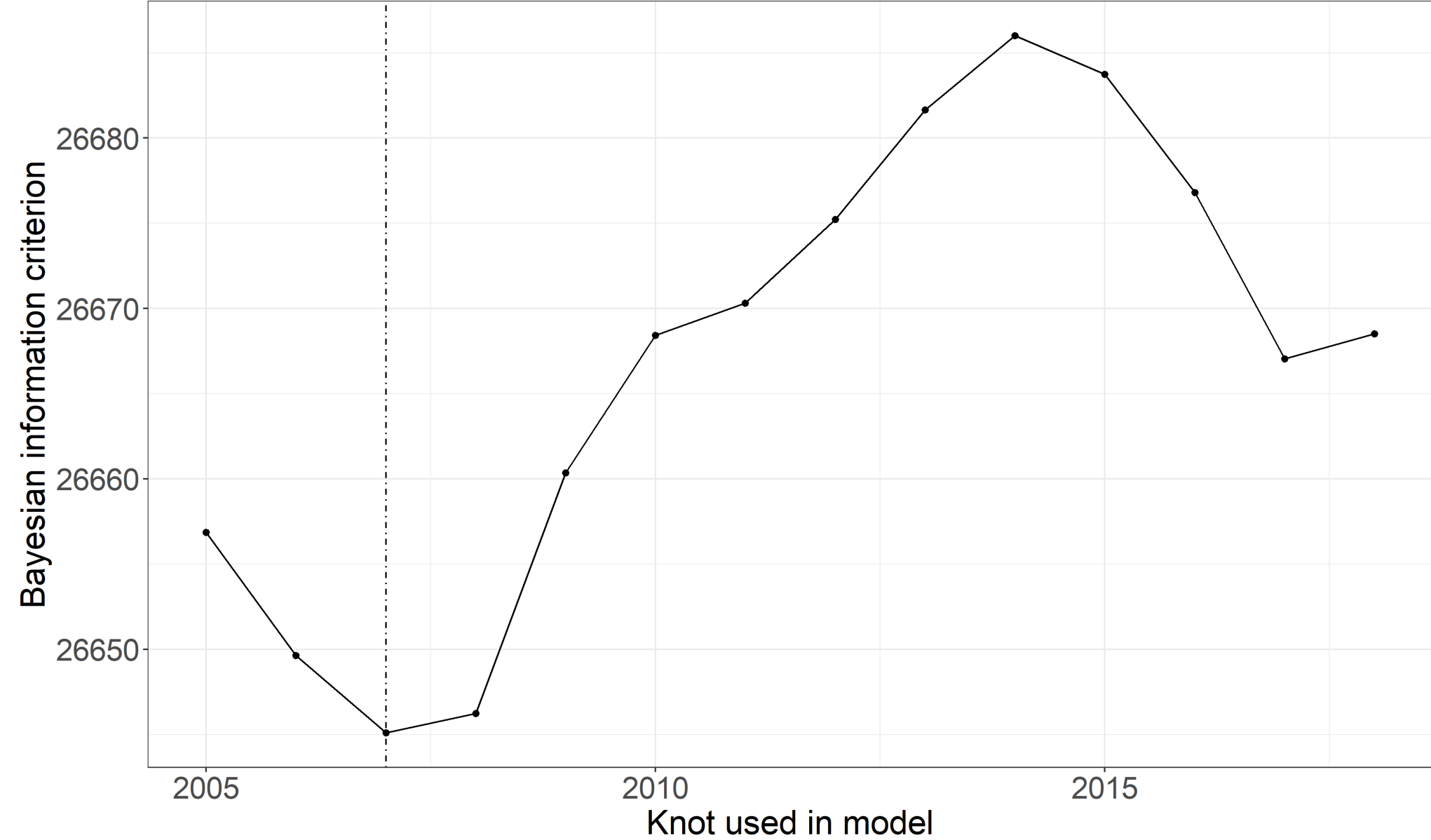

Supplement: Supplementary data [file bmjopen-2020-041774supp019.pdf]

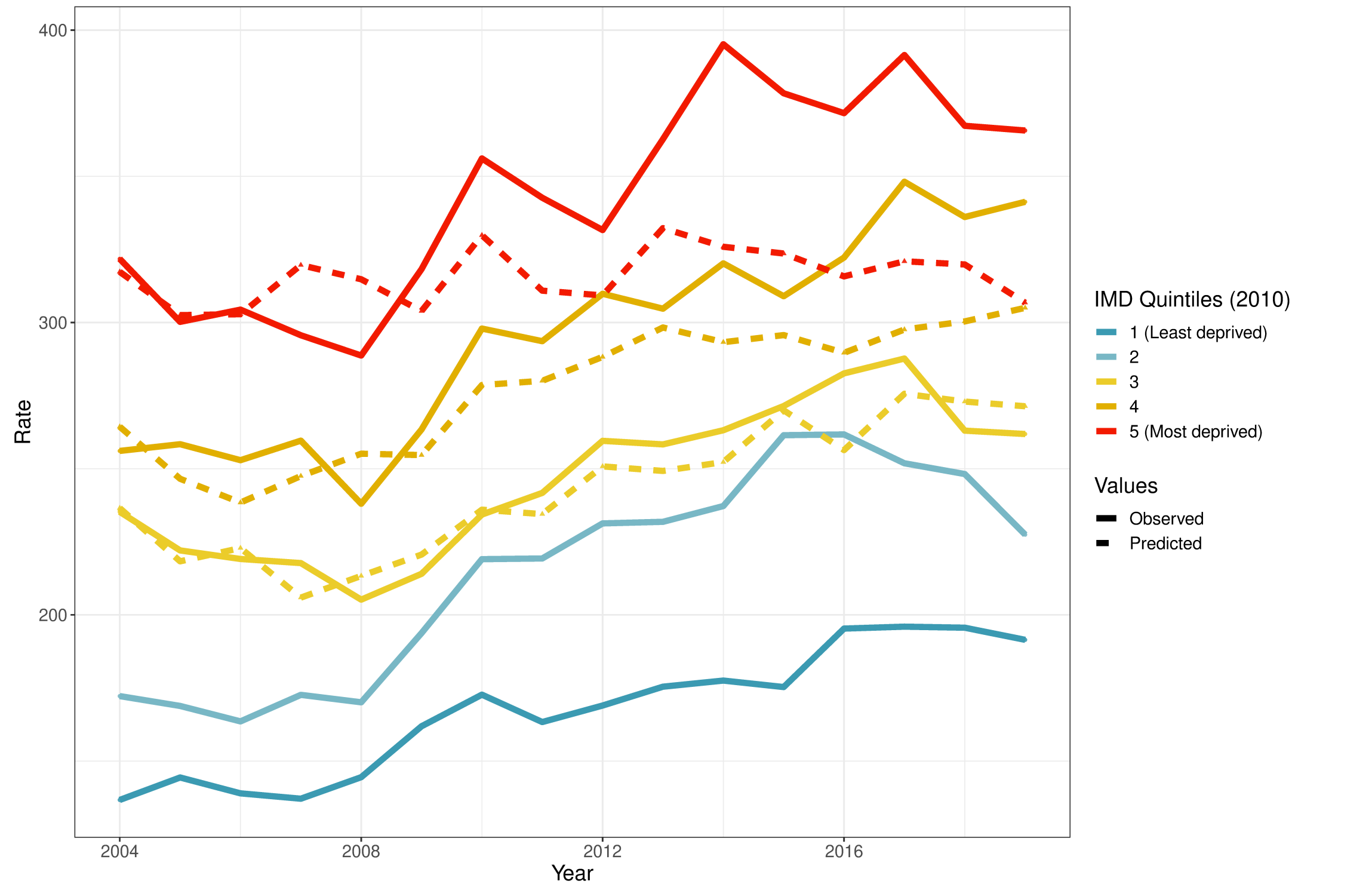

Supplement: Supplementary data [file bmjopen-2020-041774supp020.pdf]
