# Supplementary material for: Trends in inequalities in Children Looked After in England between 2004 and 2019: a local area ecological analysis
Source: BMJ Open. 2020 Nov 23;10(11):e041774. doi: 10.1136/bmjopen-2020-041774 (PMC7684833; doi:10.1136/bmjopen-2020-041774)
Supplement: Author's manuscript [file bmjopen-2020-041774.draft_revisions.pdf]

BMJ Open is committed to open peer review. As part of this commitment we make the peer review history of every article we publish publicly available.

When an article is published we post the peer reviewers' comments and the authors' responses online. We also post the versions of the paper that were used during peer review. These are the versions that the peer review comments apply to.

The versions of the paper that follow are the versions that were submitted during the peer review process. They are not the versions of record or the final published versions. They should not be cited or distributed as the published version of this manuscript.

BMJ Open is an open access journal and the full, final, typeset and author-corrected version of record of the manuscript is available on our site with no access controls, subscription charges or pay-per-view fees (http://bmjopen.bmj.com).

If you have any questions on BMJ Open's open peer review process please email info.bmjopen@bmj.com

BMJ Open

Trends in inequalities in looked after children in England 2004-2019: a local area ecological analysis

Journal:	*BMJ Open*
Manuscript ID	bmjopen-2020-041774
Article Type:	Original research
Date Submitted by the Author:	19-Jun-2020

Complete List of Authors:	Bennett, Davara; University of Liverpool Faculty of Health and Life Sciences, Department of Public Health and Policy<br>Mason, Kate; University of Liverpool Faculty of Health and Life Sciences, Department of Public Health and Policy<br>Schlüter, Daniela; University of Liverpool Faculty of Health and Life Sciences, Department of Public Health and Policy<br>Wickham, S; University of Liverpool Faculty of Health and Life Sciences, Department of Public Health and Policy<br>Lai, Eric; University of Liverpool Faculty of Health and Life Sciences, Department of Public Health and Policy<br>Alexiou, Alexandros; University of Liverpool Faculty of Health and Life Sciences, Department of Public Health and Policy<br>Barr, Ben ; University of Liverpool Faculty of Health and Life Sciences, Department of Public Health and Policy<br>Taylor-Robinson, David; University of Liverpool Faculty of Health and Life Sciences, Department of Public Health and Policy

Keywords:	Child protection < PAEDIATRICS, PUBLIC HEALTH, Community child health < PAEDIATRICS

SCHOLARONE™
Manuscripts

I, the Submitting Author has the right to grant and does grant on behalf of all authors of the Work (as defined in the below author licence), an exclusive licence and/or a non-exclusive licence for contributions from authors who are: i) UK Crown employees; ii) where BMJ has agreed a CC-BY licence shall apply, and/or iii) in accordance with the terms applicable for US Federal Government officers or employees acting as part of their official duties; on a worldwide, perpetual, irrevocable, royalty-free basis to BMJ Publishing Group Ltd ("BMJ") its licensees and where the relevant Journal is co-owned by BMJ to the co-owners of the Journal, to publish the Work in this journal and any other BMJ products and to exploit all rights, as set out in our licence.

The Submitting Author accepts and understands that any supply made under these terms is made by BMJ to the Submitting Author unless you are acting as an employee on behalf of your employer or a postgraduate student of an affiliated institution which is paying any applicable article publishing charge ("APC") for Open Access articles. Where the Submitting Author wishes to make the Work available on an Open Access basis (and intends to pay the relevant APC), the terms of reuse of such Open Access shall be governed by a Creative Commons licence – details of these licences and which Creative Commons licence will apply to this Work are set out in our licence referred to above.

Other than as permitted in any relevant BMJ Author's Self Archiving Policies, I confirm this Work has not been accepted for publication elsewhere, is not being considered for publication elsewhere and does not duplicate material already published. I confirm all authors consent to publication of this Work and authorise the granting of this licence.

For peer review only

TITLE PAGE

Trends in inequalities in looked after children in England 2004-2019: a local area ecological analysis

Davara L Bennett[1] MSc, Kate E Mason[1] PhD, Daniela K Schlüter[1] PhD, Sophie Wickham[1] PhD, Eric TC Lai[1] PhD, Alexandros Alexiou[1] PhD, Ben Barr[1*] PhD, David Taylor-Robinson[1*] PhD

1. University of Liverpool, Faculty of Health and Life Sciences, Department of Public Health and Policy.

- Joint Senior Author

Correspondence to: Davara L Bennett, University of Liverpool, Waterhouse Building 2nd Floor Block F, L69 3GL, Liverpool, United Kingdom

E-mail: davara.bennett@liverpool.ac.uk

Word Count: 3,153

For peer review only

ABSTRACT

Objective: To determine whether there were inequalities in the precipitous rise in rates of children becoming looked after (CLA) in England over the last decade, and the contribution of unemployment, a marker of the recession, to these trends.

Setting: 150 English upper-tier local authorities.

Participants: Children under the age of 18.

Primary outcome measure: The annual age-standardised rate of children becoming looked after (CLA rate) across English local authorities, grouped into quintiles based on their level of income deprivation. Slope indices of inequality (SII) were estimated using longitudinal segmented mixed effects models.

Results: Since 2008, CLA rates have risen dramatically, with a greater rise in the most disadvantaged quintiles. Between 2007 and 2019 the gap between most and least deprived areas increased by 15 children per 100,000 per year (95% CI 4-26) relative to the previous trend. The financial recession was independently associated with rising CLA rates. For each percentage point increase in the unemployment rate, an estimated additional 9 children per 100,000 (95% CI 6-11) per year became looked after the following year.

Conclusions: The dramatic rise in the numbers of CLA has been greater in poorer areas, and in areas more deeply affected by the recession. Policies should urgently address inequalities in the determinants of children becoming looked after at local area level.

For peer review only

STRENGTHS AND LIMITATIONS OF THE STUDY

- This study is the first to quantify inequalities in child welfare outcomes in England longitudinally, to show that the gap in rates of children entering care between most and least deprived areas is on the rise. This finding is consistent with the hypothesis that adverse socioeconomic conditions precipitated by policies subsequent to the recession have exacerbated inequalities in child welfare outcomes.
- The study uses routinely available data for the whole of England, and explores several child welfare outcomes in order to describe trends throughout the child welfare system.
- An important limitation is that, using an ecological area-level analysis, we cannot conclude that children entering care were directly affected by the exposures of interest.

For peer review only

INTRODUCTION

Improving the health outcomes and life chances of children looked after (CLA) is a matter of public health concern (1). In England, over the last decade, the prevalence of CLA increased dramatically, from 53 to 64 per 10,000 children, a rise of 20 percent. At last count, in March 2019, their number exceeded 78,000 (2). The health outcomes and life chances of these children, many of whom have experienced abuse, neglect and other forms of acute adversity, may differ markedly from those of their peers. On average, individuals who have been looked after face worse outcomes across a range of measures, throughout the life-course – physical and mental health, education, offending, employment, income – relative to those who have not come in contact with child welfare services. (3)

Reducing the economic burden associated with the consequences of CLA is of particular concern to policymakers: supporting CLA represents a major expenditure at local authority (LA) level. Across England, between 2011 and 2018, CLA spend increased by £1.9bn in real terms, to £4.6bn. Children's services have been described as approaching breaking point (4). Internationally, there have been increasing calls for a preventative approach to CLA that addresses upstream risk factors for child abuse and neglect (5).

A number of factors may have contributed to rising CLA rates in England over the last decade. High profile serious case reviews (6), shifting understanding of the impact of different forms of childhood adversity (7), and legal judgements clarifying LA statutory responsibilities (8), may all affect thresholds for child welfare intervention. Wider economic changes may also underlie trends in CLA rates. Growing up in adverse socioeconomic circumstances (SECs) is an important risk factor for child abuse and neglect and for children being taken into care (9), with poverty, unemployment and parental financial stress recognised as contributory causal factors (10,11). Several experimental and quasi-experimental studies from the US have shown that raising family income and reducing poverty leads to a reduction in rates of child abuse and neglect (10,12).

In 2008, the onset of the financial recession led to rising unemployment in England, and to fiscal policy with far-reaching social consequences. In 2010 the UK government began introducing a series of austerity measures with the stated intention of eliminating the budget deficit and reducing the national debt(13). The welfare system has been a principal focus of cuts and reforms (14). These have adversely affected, in particular, families with children and those at greatest risk of poverty, fuelling a rise in child poverty (15). At the same time,

For peer review only

regressive cuts to LA budgets have led to reduced spending on early childhood education and care, and other prevention services(16). Whilst increases in unemployment during the recession were dispersed across all parts of the country, changes in welfare provision and cuts to prevention have disproportionately affected deprived areas (17). If these changes are leading to increased incidence of child abuse and neglect, we would expect CLA rates to rise more rapidly in more deprived areas.

There are stark differences in rates of CLA across LAs in England (1). Less clear is how these are changing over time. Our aim in this study is to determine whether the rate of children becoming looked after increased more in deprived areas of the country, and the contribution of unemployment, a marker of recession, to these trends. We further quantify trends in inequalities in children experiencing other forms of child welfare intervention, in order to assess whether findings for CLA are consistent across child welfare outcomes.

METHODS

Data sources and measures

We undertook segmented longitudinal analysis of CLA rates at local authority level. We used routinely available data from 150 English upper-tier LAs between 2004 and 2019, based on 2010 boundaries (see appendix 1). Two LAs, the City of London and the Isles of Scilly, were excluded due to their small population size.

Our primary outcome of interest was the annual age-standardised rate of children becoming looked after by LAs in England (hereafter referred to as ‘CLA rate’). Panel data for the number of CLA, by age group, were drawn from the ‘children looked after data return’, submitted by LAs to the Department for Education on 31st March annually (2). We refer to the financial year by the latter year throughout. Direct age standardisation was performed using the national population distribution of children.

Secondary outcomes captured the wider population of children known to children’s social care. Figure 1 outlines the different child welfare outcomes. We used the annual age standardised rate of children becoming the subject of a Child Protection Plan (‘CPP rate’), and children beginning an ‘episode of need’ (‘CIN rate’). Data for these outcomes between 2010 and 2019 were sourced from the Children in Need (CIN) Census records of children referred for social care support in England (18). For children on a CPP, a breakdown of

For peer review only

numbers by category of abuse were available. Disaggregation by age group was requested via a Freedom of Information request, and obtained for years 2012 to 2019.

As a measure of SEC, we used the income deprivation score of the 2010 Indices of Multiple Deprivation (19). This is a non-overlapping count of individuals who, as a result of low earnings, qualify for means-tested benefits, as a proportion of the total population (20). We used 2010 scores based on 2008 data, collected prior to the implementation of austerity policies. For our categorical measure of deprivation, LAs were assigned to quintiles according to their score such that 20% of the 2008 child population was apportioned to each quintile. We also used a continuous measure of the income deprivation score, converted to a weighted rank by assigning a value from 0 to 1 based on the midpoint of the LA's range in the cumulative distribution. When using this value as a continuous exposure variable in the regression model, the estimated coefficient expresses the Slope Index of Inequality (SII), a commonly used indicator of the association between health outcomes and socioeconomic deprivation (21). In our statistical analyses, the SII represents the absolute difference in child welfare outcomes between LAs of lowest and highest levels of income deprivation, taking into account the distribution of the child population across LAs (22).

Our analyses also included LA unemployment rates as a covariate in order to separate out the impact of the recession on child welfare outcomes. We used data on the number of people claiming Jobseeker's Allowance, plus those claiming Universal Credit who are out of work, as a proportion of residents aged 16-64, in the financial year(23). Since the effect of unemployment on child welfare outcomes are unlikely to be immediate, we lagged the variable by one year.

Statistical analysis

First, we assessed descriptive trends for our outcome CLA rate, across LAs grouped into quintiles of income deprivation, between 2004 and 2019. Second, we estimated a segmented linear regression model, with: age-standardised CLA rate as the outcome; year, unemployment rate and income deprivation weighted rank as continuous independent variables; and random intercept and slope terms to account for the correlation between measurements within LAs. Based on our initial descriptive analysis, we included a linear spline for the effect of calendar year, with one knot indicating the timing of the change in trend. We used an iterative search procedure to confirm the knot position resulting in the model with the smallest Bayesian Information Criterion value (24,25). We included an

For peer review only

interaction between the spline terms for the effect of year and deprivation to allow for potential differences in trend by SEC. Full details are provided in appendices 2-3.

We used this model to assess whether there was a significant change in the trend in CLA over this period, whether this differed by level of LA income deprivation, and the potential contribution of unemployment to trends in our outcome. We estimated all model parameters by maximum likelihood, using generalized likelihood ratio statistics to compare nested models, and Wald statistics to test hypotheses about model parameters. Similar models were fitted for each of our secondary outcomes, CPP and CIN rates, across years for which data were available, 2012-2019 – based on our descriptive analysis no linear splines were included in these models. Models were estimated using the lme4 package (26), in R version 3.5.1. We carried out supplementary analyses, assessing descriptive trends for all outcomes stratified by age, and, for CPP, by category of abuse (appendices 4-5), and making predictions based on the model (appendix 6).

Patient and Public Involvement

The research question was informed by early conversations with policymakers and practitioners in the Merseyside area, and reflects the evidence needs identified by senior leaders within Children's Social Care in a priority-mapping exercise facilitated by the What Works Centre for Children's Social Care (27). Early plots were shared with local contacts, and the ensuing discussions informed our hypotheses about drivers of recent trends, in particular age-stratified trends. These hypotheses have informed our research agenda.

RESULTS

Trends in child welfare outcomes

Figure 2 shows CLA rate, by LA income deprivation quintile. Between 2004 and 2008, overall CLA rates dipped slightly: a small increase in the most affluent quintile was offset by decreases in more deprived areas. In 2008, the absolute difference in CLA rate between most and least deprived quintiles was 144 per 100,000 (95% CI 104-184). From around 2008, there was a change in trend and CLA rates rose. A social gradient in CLA is apparent throughout, with the absolute difference between most and least deprived quintiles rising to 174 per 100,000 (95% CI 127-221) in 2019, an increase of 21% from 2008.

Figure 3 shows the CPP and CIN rates. As with CLA rates, CPP rates have risen since 2012, and show a clear social gradient. However, the increase occurred relatively evenly

For peer review only

across all groups of LAs, in all age groups. CIN rates also exhibit a social gradient, but trends appear to be relatively stable over time.

Supplementary analyses (appendix 4, appendix figures 2-4) show that the gap in CLA rates between most and least deprived quintiles differed by age. The gap is wide, but relatively stable over time in the youngest age group, children under 1. The gap is widening in the oldest age group, those aged 16-17. Finally, stratifying CPP rates by category of abuse complicates the overall picture of an even rise in rates across all LA income quintiles: we uncovered a widening gap between most and least deprived areas in rates of children becoming subject to a CPP due to concerns about emotional abuse (appendix 5, appendix figure 5).

Segmented linear regression models

Tables 1-2 summarise the results of the segmented regression analyses. For full model output and residual diagnostics, see appendices 7-8 (appendix figures 7-15). For CLA, a knot in 2007, ahead of the 2008 change in trend identified in our descriptive analysis, resulted in the best model fit, indicating a change in trend at this point (appendix 3, appendix figure 1). In our model, rising unemployment in the wake of the financial recession was independently associated with rising CLA rates: for each percentage point increase in the unemployment rate, an estimated additional 9 children per 100,000 (95% CI 6-11) per year entered care the following year. There were no associations between CPP and CIN rates and unemployment rates.

Table 1: Association between outcomes and unemployment rate

Outcome and time period	Annual change (in children per 100,000) for a 1% increase in the unemployment rate the previous year [95% CI]
**CLA rate,** 2004-19	9.0 [6.5, 11.4]
**CPP rate,** 2012-19	-10.4 [-22.2, 1.4]
**CIN rate,** 2012-19	68.5 [-3.1, 140.1]

But unemployment rates do not account for differences in trends between more and less deprived LAs. In 2004, after controlling for LA unemployment, the SII was 193, indicating that there were 193 more CLA per 100,000 in the most deprived areas, compared to the least deprived (95% CI 140-246). Between 2004 and 2007, this gap declined by 11

For peer review only

children per 100,000 per year (95% CI 0-22) (table 2). From 2007 there was a significant change in the trend in inequalities: the gap increased by 15 children per 100,000 per year (95% CI 4-26) relative to the previous trend. Altogether, based on our model, we estimate that an additional 18,567 (95% CI 3,553 - 33,394) children were taken into care between 2007 and 2019, than would have been expected had the rise from 2007 occurred in more deprived LAs as it did in the median LA (appendix 6, appendix figure 6).

Table 2: Trends in the Slope Index of Inequality across child welfare outcomes

Outcome and time period	Annual change (in children per 100,000) in the Slope Index of Inequality [95% CI]
**CLA**	
2004-07	-11.4 [-22.3, -0.5]
2007-19, relative to previous trend	14.9 [3.6, 26.2]
**CPP**	
2012-19	4.4 [-11.2, 20.0]
**CIN**	
2012-19	47.1 [-62.7, 156.9]

DISCUSSION

Main findings

The dramatic rise in CLA in England since 2008 has been greater in poorer areas of the country, increasing inequalities. Overall an additional 18,567 (95% CI 3,553 - 33,394) children were taken into LA care between 2007 and 2019 than would be expected if the rise from 2007 had occurred more evenly across LAs. These findings are consistent with our hypothesis that austerity measures may have contributed to rising rates of child welfare outcomes. Our analysis also shows that the rise in CLA was associated with rising unemployment at LA level, a marker of the recession.

Trends in inequalities in CLA are not simply mirroring broader trends throughout the ‘funnel’ of children’s social care. Whilst CPP rates are also rising, and show a clear social gradient, we did not find a greater increase in more deprived compared to less deprived areas for children becoming the subject of a CPP and beginning an episode of need.

Strengths and limitations

This study is the first to quantify inequalities in child welfare outcomes longitudinally. A strength is that it uses routinely available data for the whole of England, and

For peer review only

explores several child welfare outcomes in order to describe trends throughout the child welfare system.

There are several important study limitations. Due to the lack of individual level data, we used an ecological area-level analysis, and cannot identify whether children entering care were directly affected by income deprivation and unemployment. Conceptually, our portrayal of children's social care as a funnel reflects a theoretical model of how a well-functioning system might operate, and may not reflect the trajectory of many individual children and families experiencing child welfare intervention. The association between income deprivation and unemployment rates and child welfare outcomes in our analysis may be due to trends in unobserved time-varying confounding factors that varied between LAs.

Trends in the data reflect the interaction between underlying need and children's services response and we interpret our findings in this light, with caution. Previous analyses by Bywater and colleagues demonstrated the existence of an "inverse intervention law" in child welfare outcomes: a greater risk of intervention in affluent compared to deprived LAs for the same level of neighbourhood deprivation (28), despite lower overall intervention rates. Our models at the level of LAs do not account for the inverse intervention law or rising thresholds reported in more deprived areas. However, this must add weight to our findings: insofar as they reflect changing underlying need, our estimates of the SII are likely to be highly conservative.

Potential explanations of our findings

Changing thresholds

Several changes during this time period may have influenced thresholds for intervention. Firstly, the death by violence of baby Peter Connelly occurred in 2007, when we see a change in the trend of CLA in our data (29). Media and political narratives that emerged in the aftermath of his death centred on the failure of children's services to intervene (30), and ensuing reports by The Children and Family Court Advisory and Support Service note a 'Baby P effect', a marked, short-term rise in applications for care orders in a risk-averse environment (6). This likely accounts for some of the change in trend and initial rise in CLA rates from 2007. Secondly, in 2009 the Southwark Judgement clarified and reinforced LAs' statutory duties in relation to 16-17 year olds presenting to the LA as homeless (8). This, together with a general shift in practice towards regarding adolescents as vulnerable children rather than nascent adults (31), and greater awareness of extra-familial forms of abuse and

For peer review only

principles of contextual safeguarding (32), may be contributing to the rising rates of 16-17 year olds across all outcomes. However, these events are unlikely to fully explain the long-term rise in CLA rates disproportionately affecting more deprived areas.

Economic trends

We found evidence of a positive association between unemployment and CLA rates. This aligns with Gillham et al.'s finding of a correlation between male unemployment and child physical abuse in Scotland in the early 1990s (33) and more recent research from the US demonstrating an association between the recession and increased risk of abuse (34). The family stress model posits that heightened stress due to adverse SECs may erode mental health and strain domestic relationships, leading to negative parenting behaviours and increased risk of child abuse and neglect. Barr et al.'s study of the mental health impact of the recession lends credence to this theorised mechanism, demonstrating an association between unemployment and mental health problems in the UK over the same period(17). Yet unemployment did not fully explain changes in CLA rates in our analysis, and unemployment rates have fallen rapidly since 2012: unemployment cannot explain the continued increase in CLA after 2012. Austerity policies subsequent to the initial recession "shock" may have compounded poor outcomes, affecting inequalities in CLA in several ways.

Changes to welfare provision and prevention

Regressive cuts to English LA budgets, with deeper cuts in more deprived areas, have precipitated a shift in expenditure away from prevention towards acute services(16). Between 2011 and 2018, spending on CLA increased by 68% in real terms, whereas spending on early years preventative services (including Sure Start) and non-statutory young people's services fell about 21%. Reports of rising thresholds for early help in more resource constrained settings, have raised concerns that we are 'storing up trouble' for the future(35). A surge in children entering care who might have benefited from early support could explain the greater rise in more deprived LAs. Adolescents may be particularly susceptible to the consequences of austerity, exposed as they are on multiple fronts, not just in the household and schools, but increasingly in the wider community. Combined cuts to welfare benefits, youth services(36), children's mental health services (37), and community policing(38), might disproportionately affect adolescents in more deprived areas, contributing to a widening inequalities in this age group. Further research is needed to investigate the impact of changing LA prevention spend on child welfare outcomes.

For peer review only

Changes to welfare benefits have led to rising child poverty, a contributory causal factor in child abuse and neglect (10,15). Averages losses in earning were particularly high in the more deprived West Midlands and the North West (15). The most vulnerable children on the edge of care, living in families already struggling to cope, may be particularly sensitive to changes in welfare benefit provision. In particular, the phased introduction of Universal Credit from 2013, with its monthly payments in arrears, enhanced conditionality and punitive sanctions, may have compounded financial stress (15) and parental mental health (39). This would increasingly lead to more children entering care in deprived areas, contributing to trends in inequalities uncovered in our study.

Policy and practice implications

We demonstrate that the increase in CLA rates from 2007 has been greater in more deprived LAs. Although it is not possible to say what constitutes an appropriate CLA rate (40), a differential rise by LA deprivation is consistent with an increase in underlying need fuelled by welfare changes and cuts to prevention services. While anti-poverty social work practice has a crucial role to play in safely reducing CLA rates and inequalities (41), this must be supported by wider policies to address the social conditions of children's lives. At the national level, this must begin with a renewed commitment to ending child poverty. Tightened social security for families with children, and increased funding for LA Children's Services, are safeguarding priorities. At the local level, holding the line on prevention services, amidst statutory pressures, may yield long-term social and economic benefits. Investment in children is key.

For peer review only

Funding and declarations of interest

DB, DT-R, BB, KM and AA are funded by the National Institute for Health Research School for Public Health Research. SW is funded by a Wellcome Trust Society and Ethics Research Fellowship (200335/Z/15/Z). DT-R and ETCL are funded by the Medical Research Council (MRC) on a Clinician Scientist Fellowship (MR/P008577/1). BB is also supported by the National Institute for Health Research Applied Research Collaboration North West Coast (ARC NWC). The views expressed in this publication are those of the authors and not necessarily those of the National Institute for Health Research or the Department of Health and Social Care, MRC or Wellcome Trust. The funders of the study had no role in study design, data collection, data analysis, data interpretation, or the writing of the report. The corresponding author had access to all of the data in the study and had final responsibility for the decision to submit for publication. We declare no competing interests.

Data sharing and ethics

The publicly available, aggregate data can be accessed from the Place-based Longitudinal Data Resource (PLDR) (42), and the code is available upon reasonable request. Research Ethics committee approval was not required for secondary data analysis.

Contributorship statement

DB is lead author and guarantor. DT-R and BB are joint senior author. DB and DT-R planned the study. DB, BB and DT-R analysed the data, supported by KM, DS, SW, ET and AA. DB, DT-R and BB led the drafting and revision of the manuscript. All authors contributed to the interpretation of the data and revision of the manuscript; all authors approved the submitted version of the manuscript. We confirm that authors have no conflicts of interest to disclose.

License statement

I, the Submitting Author has the right to grant and does grant on behalf of all authors of the Work (as defined in the below author licence), an exclusive licence and/or a non-exclusive licence for contributions from authors who are: i) UK Crown employees; ii) where BMJ has agreed a CC-BY licence shall apply, and/or iii) in accordance with the terms applicable for US Federal Government officers or employees acting as part of their official duties; on a worldwide, perpetual, irrevocable, royalty-free basis to BMJ Publishing Group Ltd ("BMJ") its licensees and where the relevant Journal is co-owned by BMJ to the co-owners of the

For peer review only

Journal, to publish the Work in Journal of Epidemiology & Community Health and any other BMJ products and to exploit all rights, as set out in our licence.

The Submitting Author accepts and understands that any supply made under these terms is made by BMJ to the Submitting Author unless you are acting as an employee on behalf of your employer or a postgraduate student of an affiliated institution which is paying any applicable article publishing charge ("APC") for Open Access articles. Where the Submitting Author wishes to make the Work available on an Open Access basis (and intends to pay the relevant APC), the terms of reuse of such Open Access shall be governed by a Creative Commons licence – details of these licences and which Creative Commons licence will apply to this Work are set out in our licence referred to above.

For peer review only

REFERENCES

1. Bywaters P. Inequalities in child welfare: Towards a new policy, research and action agenda. British Journal of Social Work. 2015;45(1):6–23.

2. Department for Education. Statistics: looked-after children (Collection) [Internet]. 2013 [cited 2020 Jun 16]. Available from: https://www.gov.uk/government/collections/statistics-looked-after-children

3. Simkiss D. Outcomes for looked after children and young people. Paediatrics and Child Health (United Kingdom). 2012;22(9):388–92.

4. Local Government Association. LGA Budget Submission: Autumn 2017. 2017.

5. O'Donnell M, Scott D, Stanley F. Child abuse and neglect - Is it time for public health approach? Australian and New Zealand Journal of Public Health. 2008;32(4):325–30.

6. CAFCASS. Annual report and accounts 2009-10. Crown; 2010.

7. Featherstone B, Gupta A, Morris K, Warner J. Let's stop feeding the risk monster: Towards a social model of 'child protection.' Families, Relationships and Societies. 2018;7(1):7–22.

8. R (on the application of G) v Southwark London Borough Council 2009.

9. Walsh D, McCartney G, Smith M, Armour G. Relationship between childhood socioeconomic position and adverse childhood experiences (ACEs): A systematic review. Vol. 73, Journal of Epidemiology and Community Health. BMJ Publishing Group; 2019. p. 1087–93.

10. Bywaters P, Bunting L, Davidson G, Hanratty J, Mason W, McCartan C, et al. The relationship between poverty, child abuse and neglect: an evidence review. 2016.

11. Hillson JMC, Kuiper NA. A stress and coping model of child maltreatment. Clinical Psychology Review. 1994;14(4):261–85.

12. Cancian M, Yang MY, Slack KS. The effect of additional child support income on the risk of child maltreatment. Social Service Review. 2013;87(3):417–37.

13. HM Treasury. Budget 2010. London; 2010.

14. Department for Work & Pensions. 2010 to 2015 government policy: welfare reform - GOV.UK [Internet]. 2010 [cited 2020 Jun 16]. Available from: https://www.gov.uk/government/publications/2010-to-2015-government-policy-welfare-reform/2010-to-2015-government-policy-welfare-reform

15. Tucker J. The Austerity Generation: the impact of a decade of cuts on family incomes and child poverty [Internet]. Child Poverty Action Group. 2017. Available from: http://www.cpag.org.uk/sites/default/files/Austerity Generation FINAL.pdf

16. Webb CJR, Bywaters P. Austerity, rationing and inequity: trends in children's and young peoples' services expenditure in England between 2010 and 2015. Local Government Studies. 2018 May 4;44(3):391–415.

17. Barr B, Kinderman P, Whitehead M. Trends in mental health inequalities in England during a period of recession, austerity and welfare reform 2004 to 2013. Social Science and Medicine. 2015;147:324–31.

18. Emmott EH, Jay MA, Woodman J. Cohort profile: Children in Need Census (CIN) records of children referred for social care support in England. BMJ Open. 2019;9(2).

For peer review only

19. Ministry of Housing Communities & Local Government. English Indices of Deprivation 2010 (Official Statistics).

20. Department for Communities and Local Government. The English Indices of Deprivation 2015: Technical report. 2015.

21. Straatmann VS, Lai E, Lange T, Campbell MC, Wickham S, Andersen AMN, et al. How do early-life factors explain social inequalities in adolescent mental health? Findings from the UK Millennium Cohort Study. Journal of Epidemiology and Community Health. 2019;73(11):1049–60.

22. Regidor E. Measures of health inequalities: Part 2. Journal of Epidemiology and Community Health. 2004;58(11):900–3.

23. ONS. NOMIS - official labour market statistics 2019.

24. Taylor-Robinson D, Lai ETC, Wickham S, Rose T, Norman P, Bambra C, et al. Assessing the impact of rising child poverty on the unprecedented rise in infant mortality in England, 2000-2017: time trend analysis. BMJ Open. 2019;9(10).

25. Barr B, Higgerson J, Whitehead M. Investigating the impact of the English health inequalities strategy: Time trend analysis. BMJ (Online). 2017;358.

26. Bates D, Mächler M, Bolker BM, Walker SC. Fitting linear mixed-effects models using lme4. Journal of Statistical Software. 2015;67(1).

27. Jones L, Scholten C, Whelan E, Clayton V, Sanders M. What Matters For What Works?: Setting research priorities for what works for Children's Social care. 2020.

28. Bywaters P, Brady G, Sparks T, Bos E, Bunting L, Daniel B, et al. Exploring inequities in child welfare and child protection services: Explaining the "inverse intervention law." Children and Youth Services Review. 2015;57:98–105.

29. Haringey Local Safeguarding Children Board. Serious Case Review "Child A." 2009.

30. Warner J. "Heads Must Roll"? Emotional Politics, the Press and the Death of Baby P. British Journal of Social Work. 2014;44(6):1637–53.

31. NSPCC. Teenagers: learning from case reviews. 2014.

32. Lloyd J, Firmin C. No Further Action: Contextualising Social Care Decisions for Children Victimised in Extra-Familial Settings. Youth Justice. 2020;20(1–2):79–92.

33. Gillham B, Tanner G, Cheyne B, Freeman I, Rooney M, Lambie A. Unemployment rates, single parent density, and indices of child poverty: Their relationship to different categories of child abuse and neglect. Child Abuse and Neglect. 1998;22(2):79–90.

34. Brooks-Gunn J, Schneider W, Waldfogel J. The great recession and the risk for child maltreatment. Child Abuse and Neglect. 2013;37(10):721–9.

35. All Party Parliamentary Group for Children. Storing Up Trouble: a postcode lottery of children's social care. 2018.

36. Kelly E, Lee T, Sibieta L, Waters T. Public Spending on Children: 2000 to 2020. 2018.

37. Children's Commissioner. Briefing: Children's Mental Healthcare in England. 2017.

38. Allen G, Audickas L. Briefing paper: Police Service Strength [Internet]. 2020. Available from: http://researchbriefings.files.parliament.uk/documents/SN00634/SN00634.pdf

For peer review only

39. Wickham S, Bentley L, Rose T, Whitehead M, Taylor-Robinson D, Barr B. Effects on mental health of a UK welfare reform, Universal Credit: a longitudinal controlled study. The Lancet Public Health. 2020;5(3):e157–64.

40. Bywaters P, Scourfield J, Jones C, Sparks T, Elliott M, Hooper J, et al. Child welfare inequalities in the four nations of the UK. Journal of Social Work. 2020;20(2):193–215.

41. Morris K, Mason W, Bywaters P, Featherstone B, Daniel B, Brady G, et al. Social work, poverty, and child welfare interventions. Child and Family Social Work. 2018;23(3):364–72.

42. Place-based Longitudinal Data Resource [Internet]. [cited 2020 Jun 16]. Available from: https://pldr.org/

For peer review only

The children's social care system has been described as series of 'filters and funnels'∞. Using this analogy, through the funnel, successive phases of risk assessment and service response determine a child's incident status:

1. **Referrals**. At the wide end of the funnel are all referrals.
2. **Children in Need**. A child is 'in need' (CIN), if deemed to require additional support in order to achieve a reasonable standard of health and development. An episode of need relates to an open case following a LA's acceptance of a referral to children's social care.
3. **Child Protection Plan**. A Child Protection Plan (CPP) may be drawn up where, following an investigation, concerns persist as to whether a child is suffering, or likely to suffer, significant harm.
4. **Children Looked After** (CLA) are at the narrow end of the funnel, enduring adversity sufficiently severe for the State to intervene in their upbringing£.

This figure represents the 'funnel' of children's social care, and shows the overlap between incident child welfare status. Size and overlaps are not to scale. Child population estimates are taken from most recent data returns, for the period 2018-19. In rare cases, due to residual safeguarding concerns, a child in care may also be subject to a CPP.

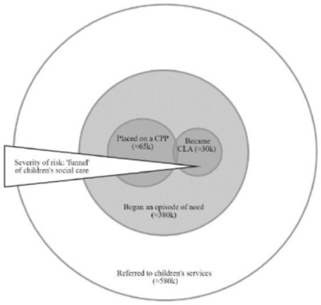

∞ Gibbons J, Bell C, Conroy S. Operating the child protection system: a study of child protection practices in English local authorities. London: HMSO; 1995.
£ Emmott EH, Jay MA, Woodman J. Cohort profile: Children in Need Census (CIN) records of children referred for social care support in England. BMJ Open. 2019;9(2):e023771.

Figure 1 - Description of the children's social care system in England

297x210mm (150 x 150 DPI)

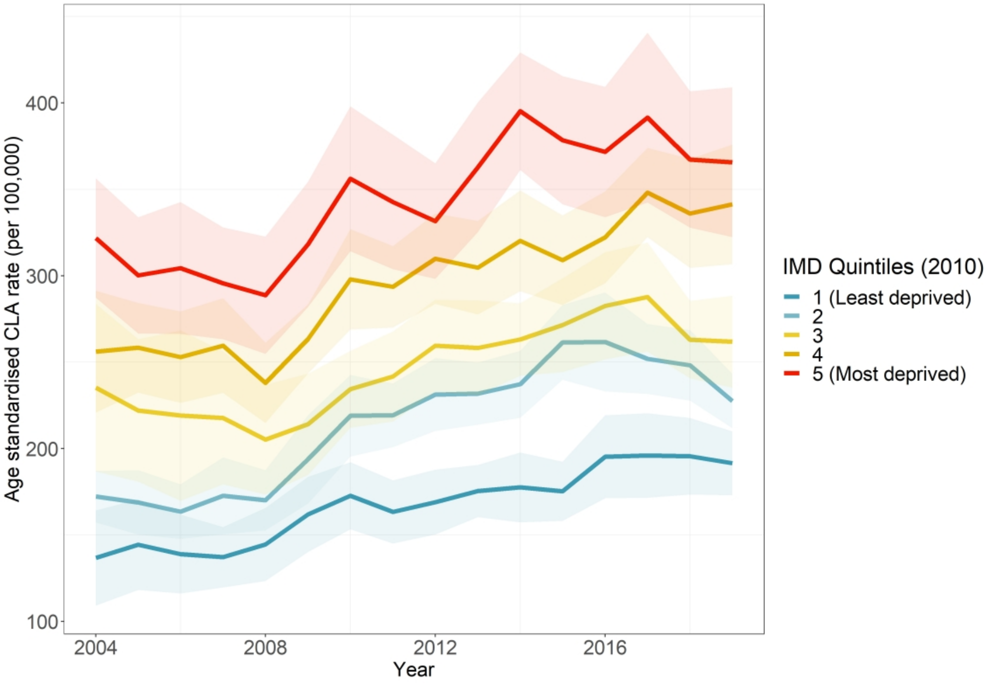

Figure 2 - CLA rates by LA income deprivation quintile, 2004-2019, with 95% CIs

319x219mm (320 x 320 DPI)

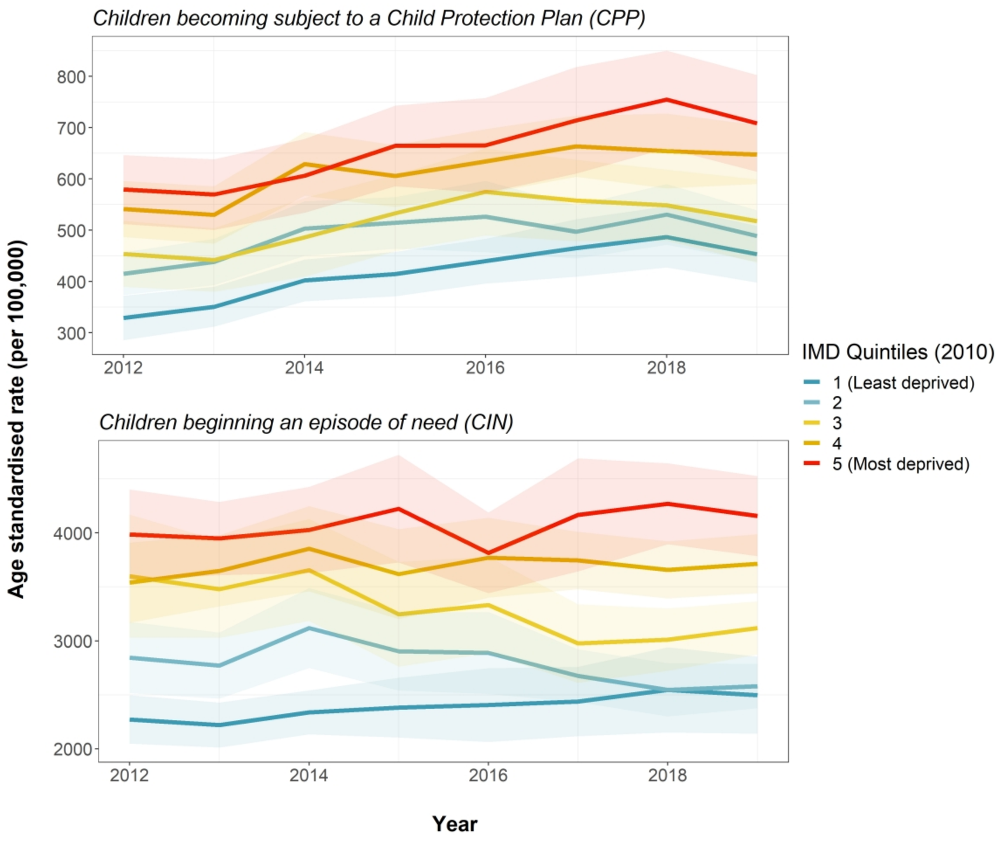

Figure 3 - CPP and CIN rates by deprivation quintile, 2012-19, with 95% CIs

299x249mm (320 x 320 DPI)

SUPPLEMENTARY MATERIALS

Appendix 1: Harmonising data

Where changes to LA boundaries in 2009 led to the formation of two upper tier unitary authorities from a single county, CLA numbers for preceding years were split between these LAs based on their 2009 child population ratio. In the publicly available data, for reasons of confidentiality, numbers from one to five inclusive were supressed. For each missing value we therefore imputed a random integer in this range. There were only three cases of missing data, across two years, early in the implementation of the CIN census: age stratified data were not available for Havering and Newham in 2012, or Norfolk in 2013. Given the low degree of missingness, we performed complete case analyses.

Appendix 2: Model formulae

Segmented linear regression model for age standardised CLA rate, including linear spline:

$$y_{ij} = \beta_0 + \beta_1 x_{1ij} + \beta_2 x_{2i} + \beta_3 x_{3j} + \beta_4 x_{4j} + \beta_5 x_{3j} x_{2i} + \beta_5 x_{4j} x_{2i} + U_i + V_i x_{3j} + \varepsilon_{ij}$$

Let:

- y_{ij} denote the rate of children taken into care in LA i in year j
- x_{1ij} denote covariate lagged unemployment rate, coded as a continuous variable and dependent on LA i and on year j
- x_{2i} denote the weighted rank of deprivation dependent on LA i, a continuous variable ranging from 0 to 1
- x_{3j} denote the first spline term, which is year j coded as continuous variable and centered at 2004
- x_{4j} denote the second spline term, a continuous variable that takes the value of 0 for year j≤2007, and j−2007 for year j>2007. This defines a segmented regression with knot in 2007.
- $(U_{i,}\ V_i) \sim BVN(0, S_0)$ denote random intercept and slope for LA i
- $\varepsilon_{ij} \sim N\ (0, S_1)$ denote the random error for LA i in year j

Linear regression model for age standardised CPP and CIN rates:

$$y_{ij} = \beta_0 + \beta_1 x_{1ij} + \beta_2 x_{2i} + \beta_3 x_{3j} + \beta_4 x_{3j} x_{2i} + U_i + V_i x_{3j} + \varepsilon_{ij}$$

Let:

- y_{ij} denote the rate of children taken into care in LA i in year j
- x_{1ij} denote covariate lagged unemployment rate, coded as a continuous variable and dependent on LA i and on year j

For peer review only

- x_{2i} denote the weighted rank of deprivation dependent on LA i, a continuous variable ranging from 0 to 1
- x_{3j} denote year j, coded as continuous variable and centered at 2004
- $(U_{i,}\ V_i) \sim BVN(0, S_0)$ denote random intercept and slope for LA i
- $\varepsilon_{ij} \sim N\ (0, S_1)$ denote the random error for LA i in year j

Appendix 3: Breakpoint analysis

In our model for age standardised CLA rates, we used an iterative search procedure in order to identify which breakpoint offered the best fit. Appendix figure 1 shows the BIC value for each successive breakpoint used in the model. This led us to fit a knot in 2007.

Appendix 4: Age stratified analyses of crude CLA, CPP and CIN rates by LA deprivation quintile

For the age stratified analyses, we calculated rates for each of our outcomes using child population data, broken down by the same age bands available in the routine and FoI data, sourced from the Office for National Statistics (ONS) mid-year population estimates, accessed via Stat-Xplore[1]. These formed our denominator. We plotted rates for all age-stratified outcomes, across years for which data were available, enabling a comparison, by age group, across outcomes. Appendix figure 2 shows that the rise in CLA rates was mainly due to children under the age of 1 and children aged 16-17 entering care. Though wide, the gap in rates between most and least deprived LAs for the youngest age group does not appear to be widening. In the oldest age group however, there is a pronounced increase in the gap from 2010.

We sought to determine whether trends in CLA were reflected through the funnel of children's social care (appendix figures 3-4). The funnel remains widest in children under the age of 1. However, the gap between most and least deprived areas is relatively stable over time regardless of the stage. In children aged 16-17, the funnel narrows considerably from CIN to CPP, then widens once more at the level of CLA. The discontinuity is unique to this age group and may relate to the CPP's focus on risks within the family home. Acute risks to older children are often in the community, from peer groups and criminal networks. This may lead children to be placed directly on a CLA when need becomes acute. The gap in rates

[1] Office for National Statistics. Population Estimates for UK, England and Wales, Scotland and Northern Ireland: Stat-Xplore; 2019 [Available from: https://stat-xplore.dwp.gov.uk].

For peer review only

between most and least deprived areas appears to be widening in both CIN and CLA for this older age group: trends in CLA may well be reflecting, and concentrating, trends in CIN.

Appendix 5: Crude CPP rates by deprivation quintile, stratified by category of abuse

We plotted CPP rates for all categories of abuse, enabling a comparison across categories (appendix figure 5). Neglect, then emotional abuse, are the most commonly recorded primary categories of abuse. Rates for these categories are rising. Where neglect is recorded, the gap in rates between most and least deprived areas appears to have declined slightly from 2014. In contrast, where emotional abuse is recorded, the gap increased dramatically from 2014. Further research is needed to understand how recording practices, child welfare systems, social care practices, and underlying need, may differ by area level income deprivation.

Appendix 6: predictions based on the model

We predict expected CLA rates if the rise in rates from 2007 had occurred in more deprived LAs as it did in the median LA (such that 50% of the 2008 child population live in more deprived areas):

$$y_{ij} = \beta_0 + \beta_1 x_{1ij} + \beta_2 x_{2i} + \beta_3 x_{3j} + \beta_4 x_{4j} + \beta_5 x_{3j} x_{2i} + \beta_5 x_{4j} \boldsymbol{x_5} + U_i + V_i x_{3j} + \varepsilon_{ij}$$

Where $\boldsymbol{x_5}$ denotes the weighted rank of deprivation in the median LA in the cumulative distribution.

This scenario preserves the change in trend from 2007 and unemployment rates, but posits that, after controlling for unemployment rates, the change in trend should not disproportionately affect areas based on their levels of income deprivation. Appendix figure 6, showing LAs grouped by quintiles, illustrates expected rates according to this scenario.

Appendix 7: Full model output[2]

The following tables summarise the full output for each of the models in turn:

- Age standardised CLA rates
- Age standardised CPP rates
- Age standardised CIN rates

[2] Hlavac M. stargazer: Well-Formatted Regression and Summary Statistics Tables. R package version 5.2.2.; 2018.

CLA, 2004-2019

	Dependent variable:
	CLA rate (per 100,000)
Intercept	104.31*** (71.56, 137.05)
Unemployment rate (lagged)	8.95*** (6.48, 11.43)
Spline 1	3.43 (-3.41, 10.27)
Deprivation	192.93*** (140.01, 245.86)
Spline 2	1.89 (-5.21, 8.99)
Spline 1: deprivation	-11.38** (-22.27, -0.49)
Spline 2: deprivation	14.86** (3.55, 26.16)
Observations	2,400
Log Likelihood	-13,279.74
Akaike Inf. Crit.	26,581.49
Bayesian Inf. Crit.	26,645.10
Note:	*p**p***p<0.01

CPP, 2012-2019

	Dependent variable:
	CPP rate (per 100,000)
Intercept	365.74*** (306.30, 425.19)
Unemployment rate (lagged)	-10.41* (-22.17, 1.36)
Year	12.69*** (3.09, 22.29)
Deprivation	304.12*** (198.42, 409.81)
Year:Deprivation	4.38 (-11.20, 19.95)
Observations	1,197
Log Likelihood	-7,599.82
Akaike Inf. Crit.	15,217.63
Bayesian Inf. Crit.	15,263.42
Note:	*p**p***p<0.01

For peer review only

CIN, 2012-2019

	Dependent variable:
	CIN rate (per 100,000)
Intercept	2,190.79*** (1,798.70, 2,582.88)
Unemployment rate (lagged)	68.52* (-3.06, 140.10)
Year	-6.76 (-74.71, 61.19)
Deprivation	1,637.02*** (949.98, 2,324.07)
Year:Deprivation	47.08 (-62.71, 156.88)
Observations	1,195
Log Likelihood	-9,727.10
Akaike Inf. Crit.	19,472.20
Bayesian Inf. Crit.	19,517.98
Note:	*p**p***p<0.01

Appendix 8: residual diagnostics

The residuals from our model are normally distributed. Plotting standard normal quantiles against the data results in a relatively linear pattern. When grouped by quintile, predicted and observed values of CLA rates appear relatively consistent:

a. CLA model: see appendix figures 7-9
b. CPP model: see appendix figures 10-12
c. CIN model: see appendix figures 13-15

For peer review only

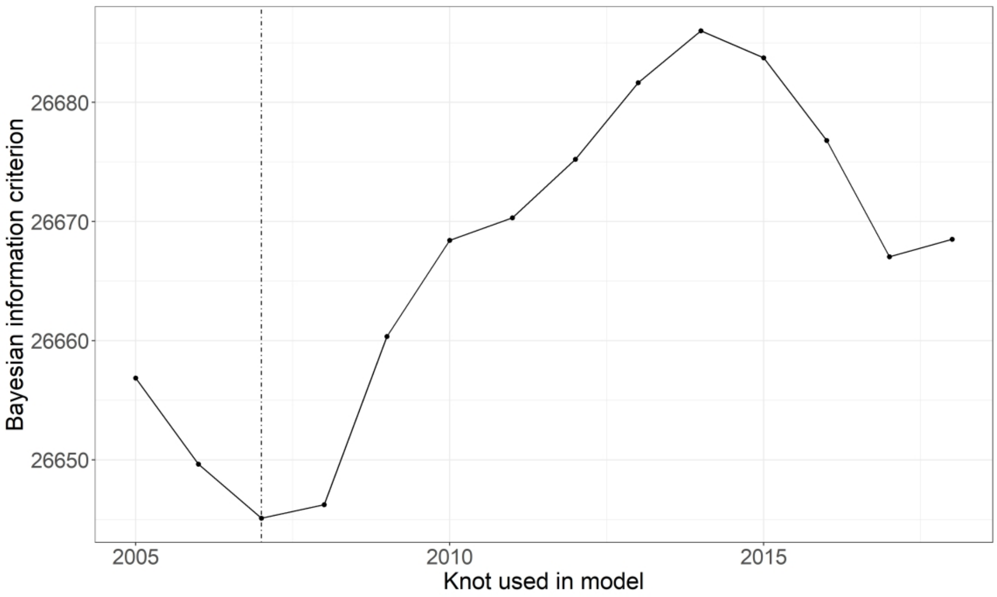

Appendix figure 1 - CLA model - breakpoint analysis

299x179mm (320 x 320 DPI)

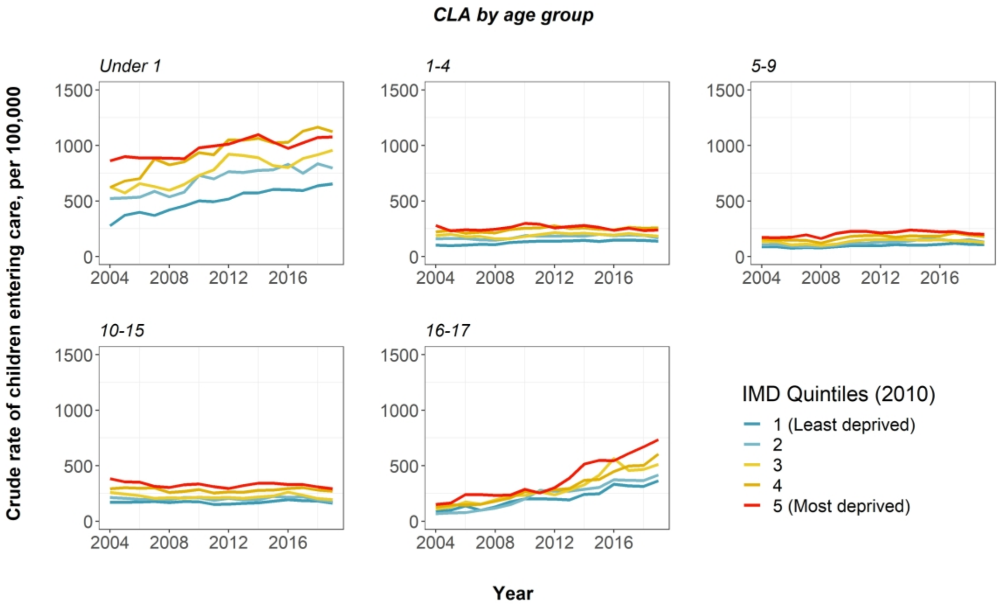

Appendix figure 2 - CLA rates by deprivation quintile, stratified by age

299x179mm (300 x 300 DPI)

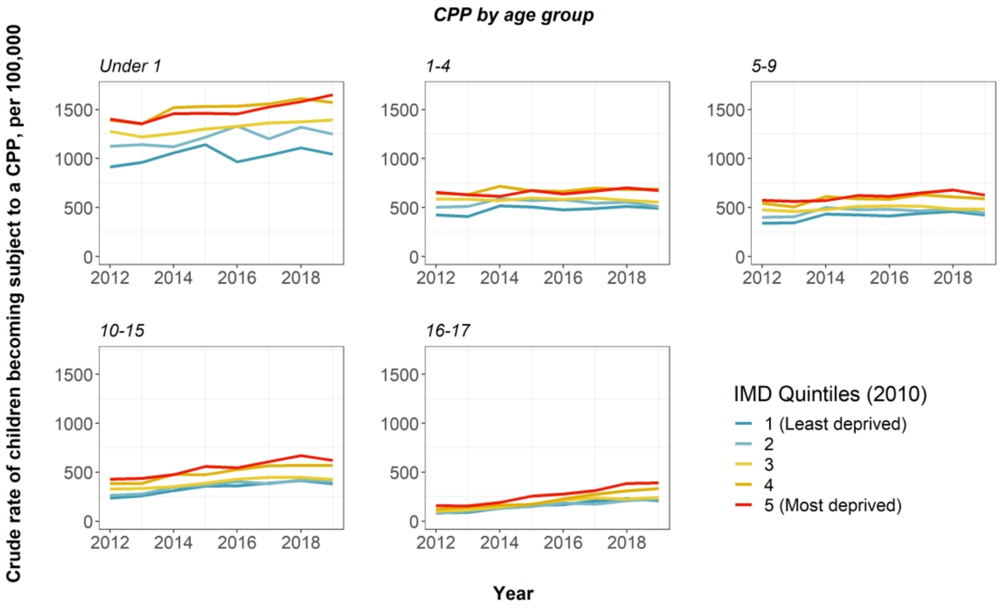

Appendix figure 3 - CPP rates by deprivation quintile, stratified by age

299x179mm (300 x 300 DPI)

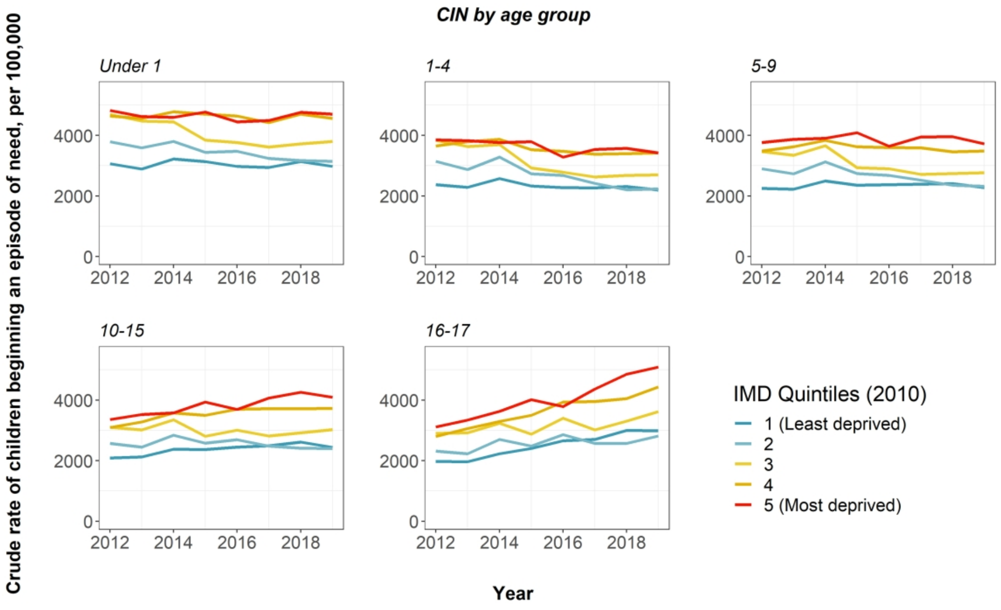

Appendix figure 4 - CIN rates by deprivation quintile, stratified by age

299x179mm (300 x 300 DPI)

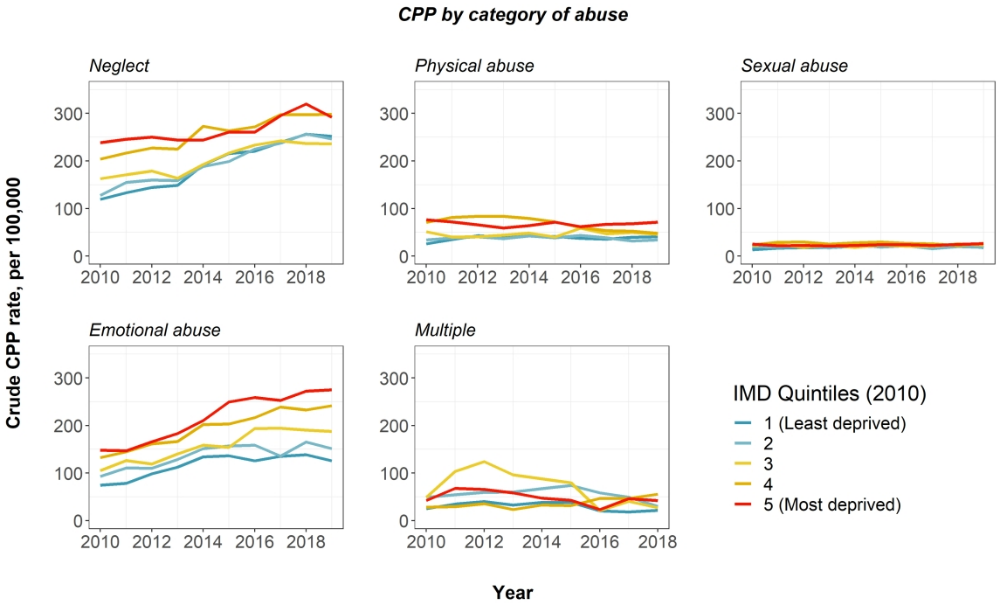

Appendix figure 5 - CPP rates by deprivation quintile, stratified by category of abuse

299x179mm (300 x 300 DPI)

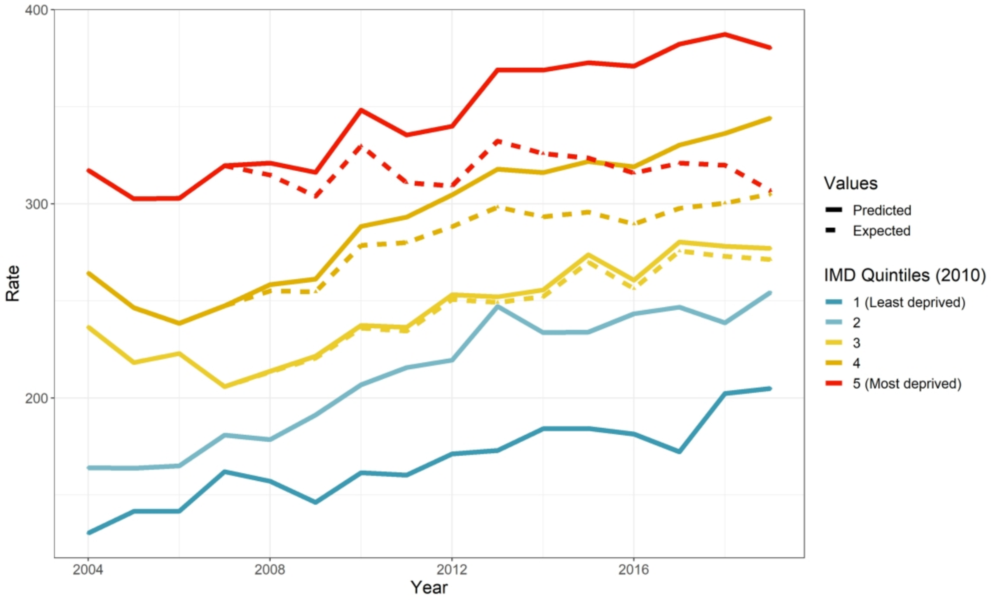

Appendix figure 6 - CLA model predictions

299x179mm (300 x 300 DPI)

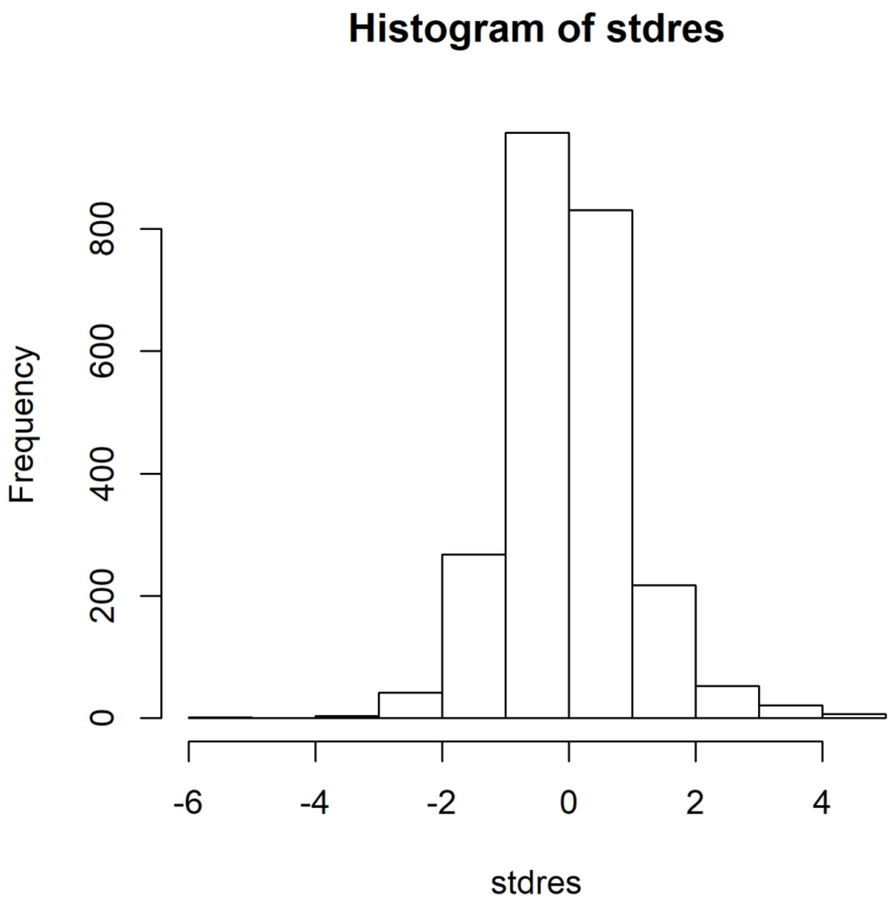

Appendix figure 7 - CLA model - histogram of standardised residuals

127x127mm (300 x 300 DPI)

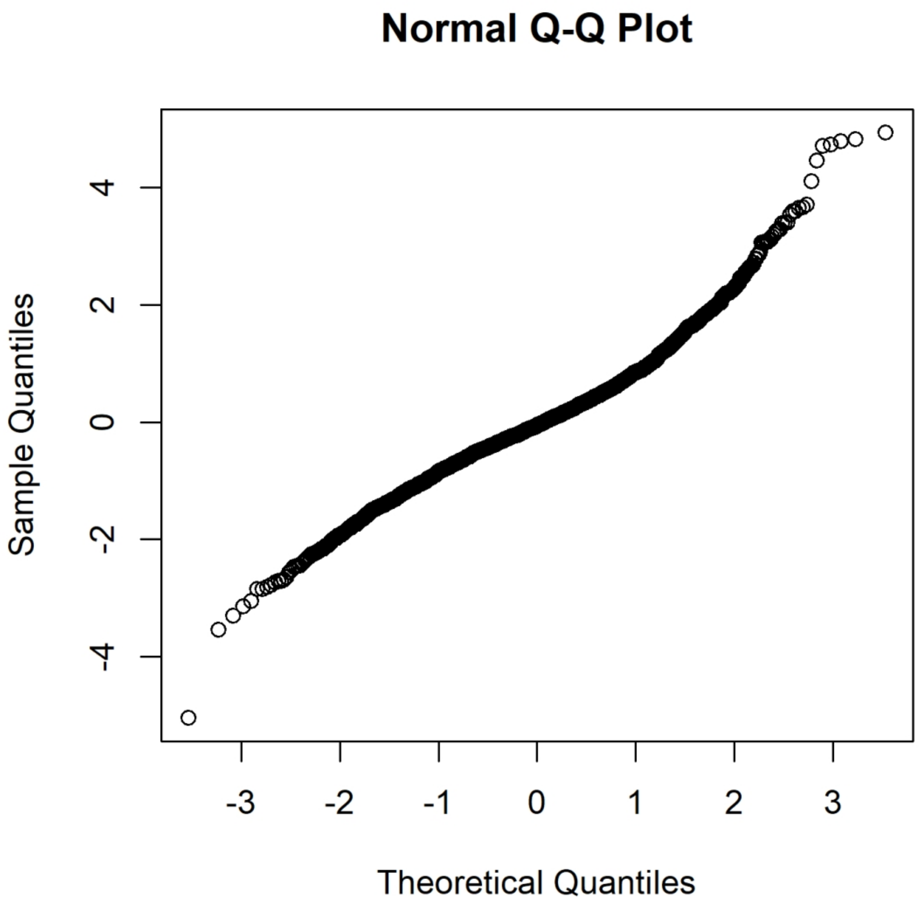

Appendix figure 8 - CLA model - quantile quantile plot

127x127mm (300 x 300 DPI)

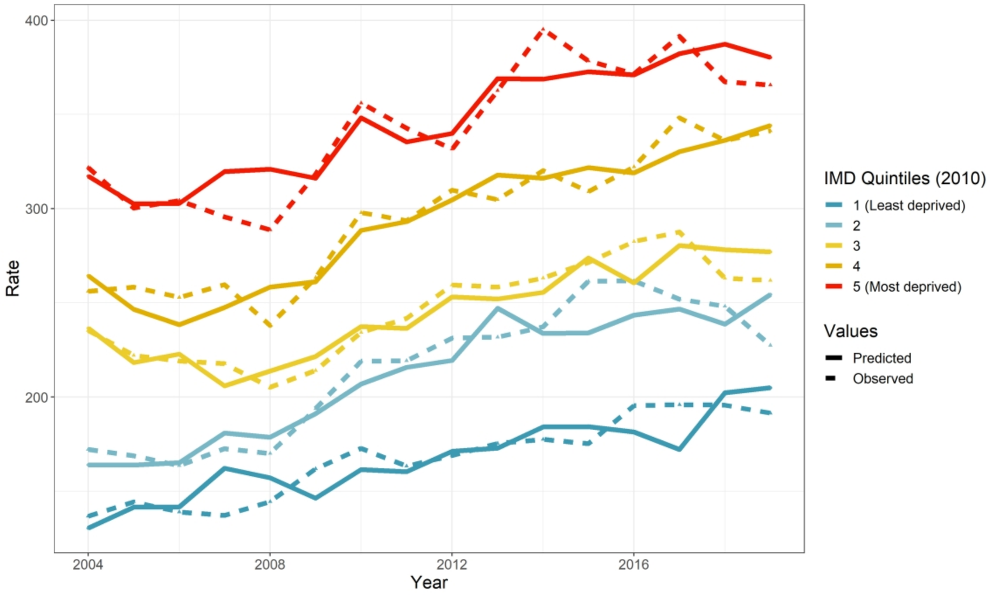

Appendix figure 9 - CLA model - comparing observed and predicted rates

299x179mm (300 x 300 DPI)

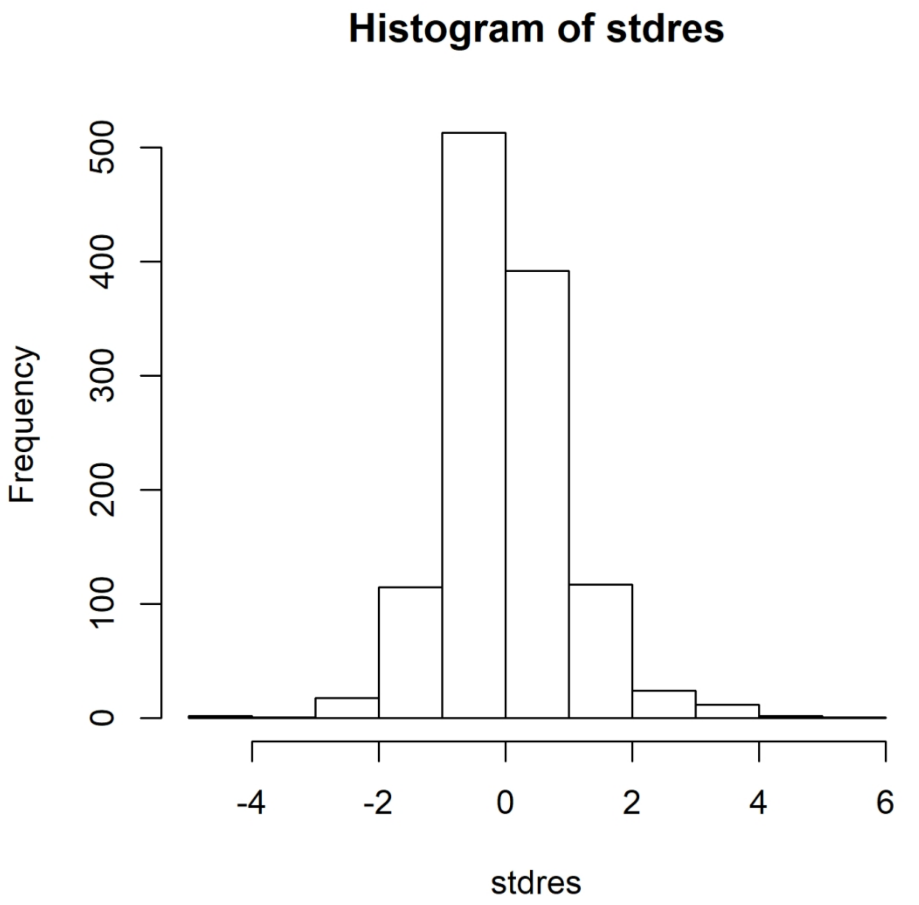

Appendix figure 10 - CPP model - histogram of standardised residuals

127x127mm (300 x 300 DPI)

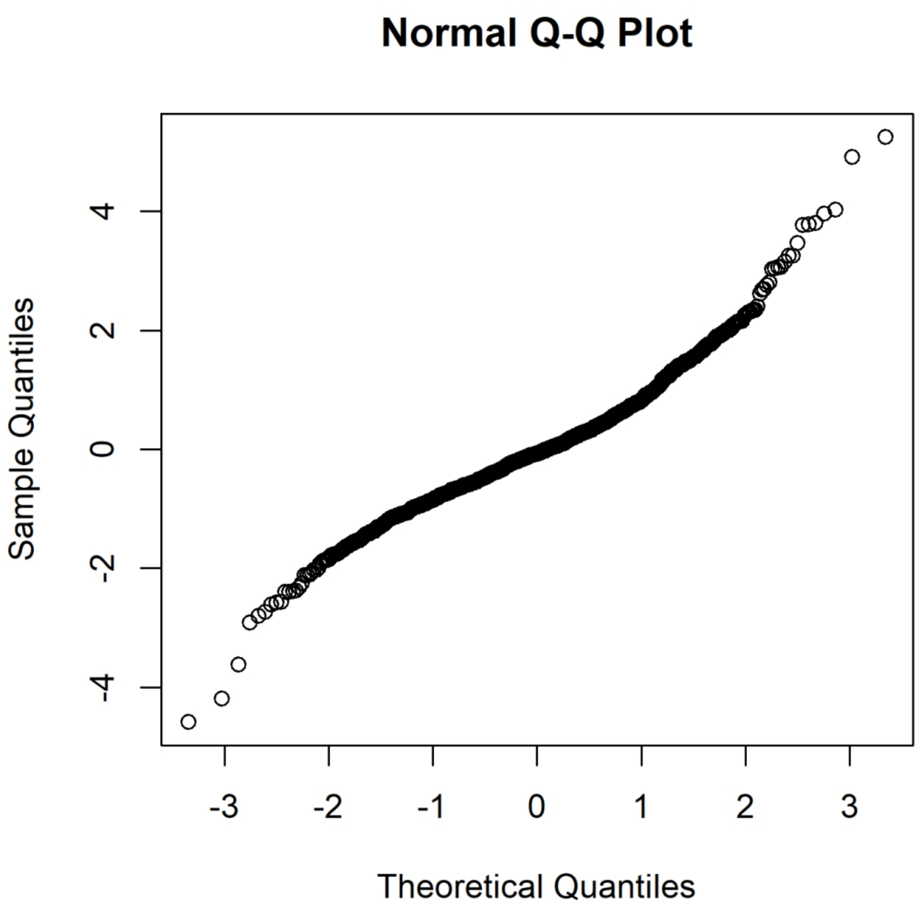

Appendix figure 11 - CPP model - quantile quantile plot

127x127mm (300 x 300 DPI)

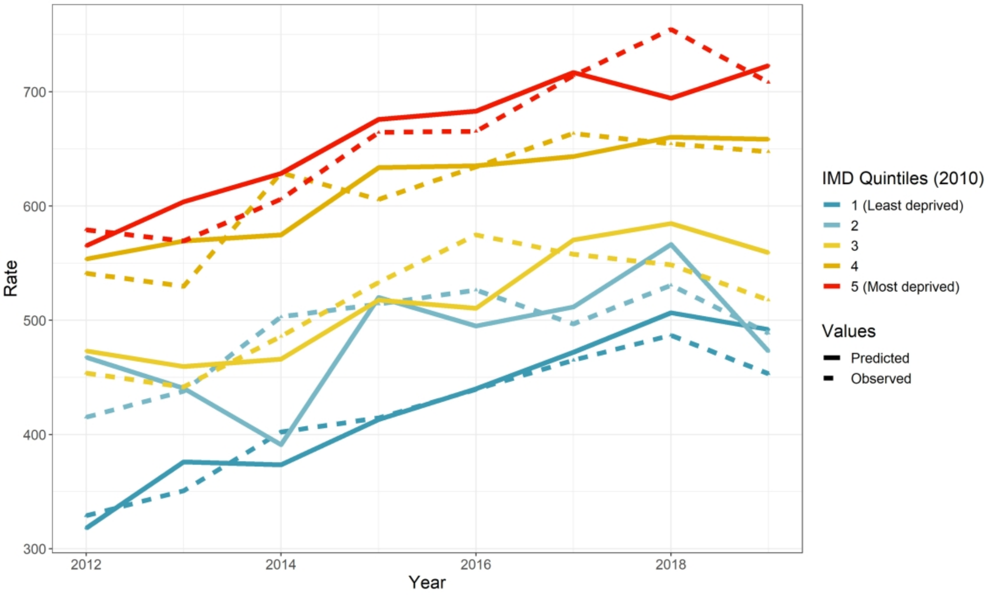

Appendix figure 12 - CPP model - comparing observed and predicted rates

299x179mm (300 x 300 DPI)

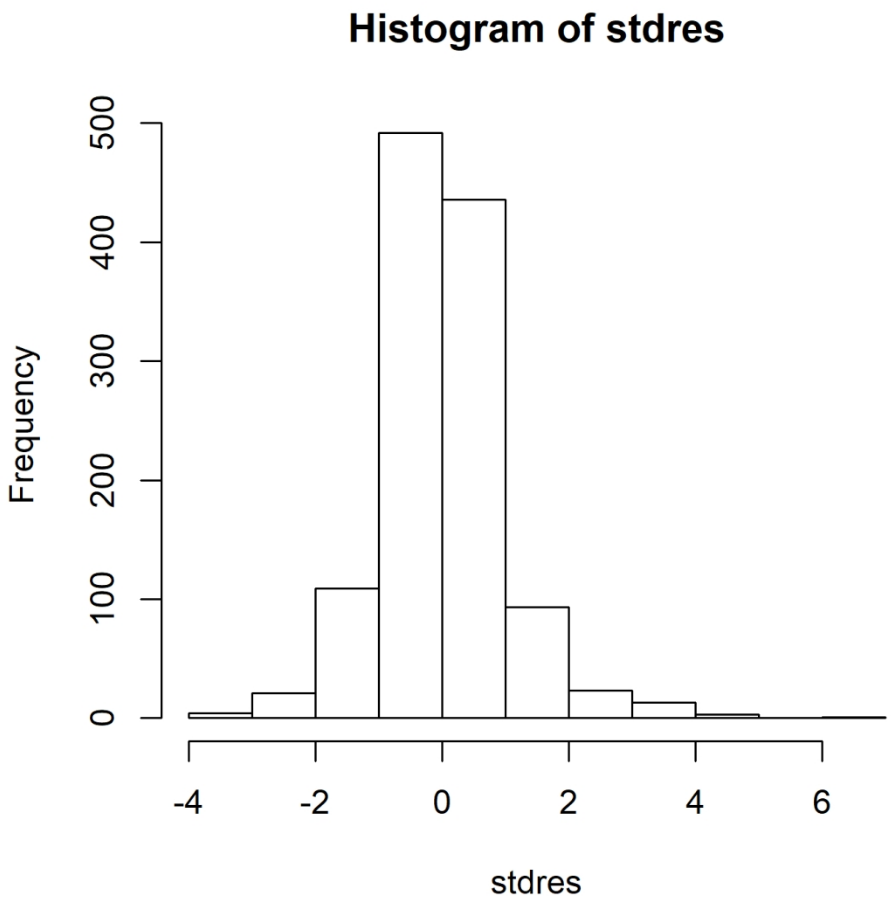

Appendix figure 13 - CIN model - histogram of standardised residuals

127x127mm (300 x 300 DPI)

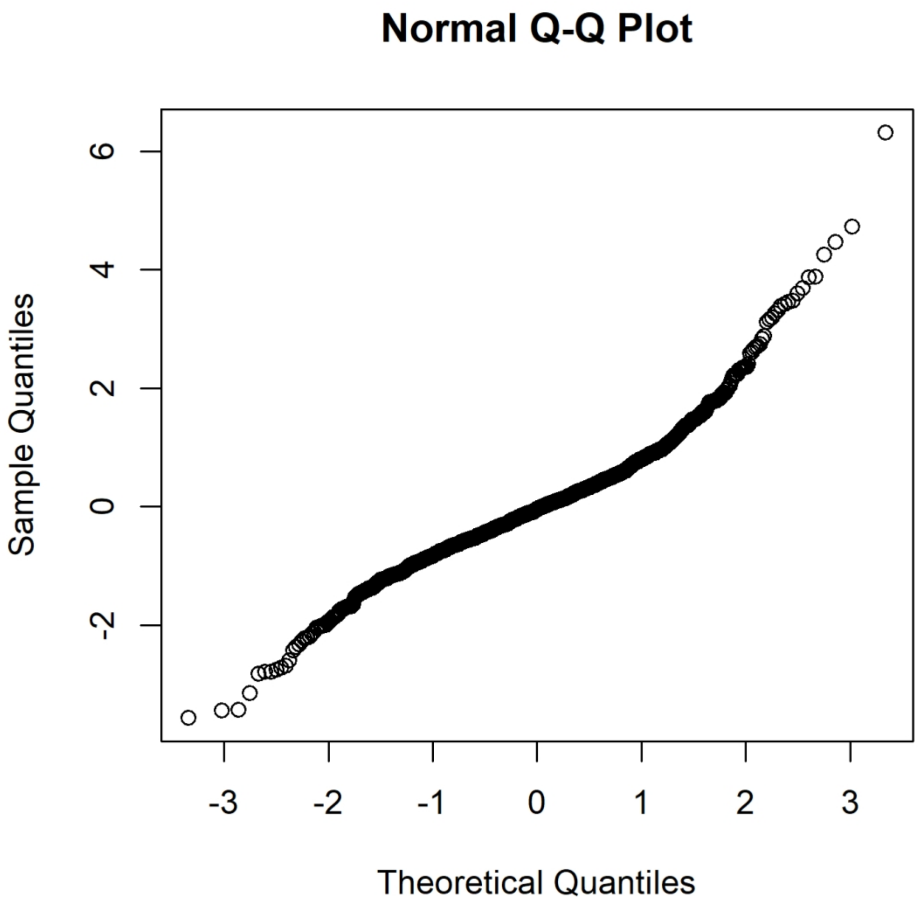

Appendix figure 14 - CIN model - quantile quantile plot

127x127mm (300 x 300 DPI)

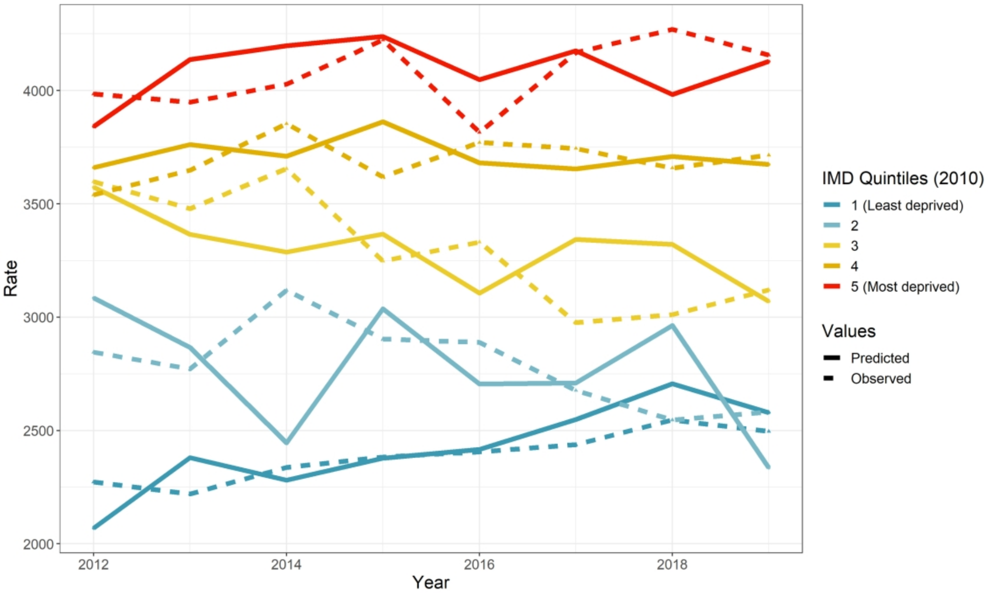

Appendix figure 15 - CIN model - comparing observed and predicted rates

299x179mm (300 x 300 DPI)

STROBE Statement—checklist of items that should be included in reports of observational studies

	Item No	Recommendation
**Title and abstract**	1	(*a*) Indicate the study's design with a commonly used term in the title or the abstract<br>Done. TITLE: "Trends in inequalities in looked after children in England 2004-2019: a local area ecological analysis"
		(*b*) Provide in the abstract an informative and balanced summary of what was done and what was found<br>Done. ABSTRACT: page 2
**Introduction**		
Background/rationale	2	Explain the scientific background and rationale for the investigation being reported.<br>Done. BACKGROUND: page 3
Objectives	3	State specific objectives, including any prespecified hypotheses<br>Done. BACKGROUND: page 2. "We investigated whether the rate of children becoming looked after between 2004 and 2019 increased more in deprived areas of the country – areas that, relative to more affluent areas, have higher levels of poverty – and the contribution of unemployment to these trends. We further quantify trends in inequalities in children experiencing other forms of child welfare intervention, in order to assess whether findings for CLA are consistent across child welfare outcomes."
**Methods**		
Study design	4	Present key elements of study design early in the paper<br>Done. METHODS: page 4
Setting	5	Describe the setting, locations, and relevant dates, including periods of recruitment, exposure, follow-up, and data collection.<br>Done. METHODS / DATA SOURCES AND MEASURES: pages 4-5
Participants	6	(*a*) *Cohort study*—Give the eligibility criteria, and the sources and methods of selection of participants. Describe methods of follow-up<br>NA: participants are the entire child population<br>*Case-control study*—Give the eligibility criteria, and the sources and methods of case ascertainment and control selection. Give the rationale for the choice of cases and controls<br>NA<br>*Cross-sectional study*—Give the eligibility criteria, and the sources and methods of selection of participants<br>NA
		(*b*) *Cohort study*—For matched studies, give matching criteria and number of exposed and unexposed<br>NA<br>*Case-control study*—For matched studies, give matching criteria and the number of controls per case<br>NA
Variables	7	Clearly define all outcomes, exposures, predictors, potential confounders, and effect modifiers. Give diagnostic criteria, if applicable.<br>Done. METHODS / DATA SOURCES AND MEASURES: pages 4-5

For peer review only - http://bmjopen.bmj.com/site/about/guidelines.xhtml

Data sources/ measurement	8*	For each variable of interest, give sources of data and details of methods of assessment (measurement). Describe comparability of assessment methods if there is more than one group.<br>Done. METHODS / DATA SOURCES AND MEASURES: pages 4-5
Bias	9	Describe any efforts to address potential sources of bias<br>Done. METHODS / DATA SOURCES AND MEASURES: pages 4-5. Use of routinely available data 2004-19 to assess descriptive trends.
Study size	10	Explain how the study size was arrived at.<br>NA: the study size is the entire child population of England, analysed as aggregate data for 150 local authorities
Quantitative variables	11	Explain how quantitative variables were handled in the analyses. If applicable, describe which groupings were chosen and why.<br>Done. METHODS/ / DATA SOURCES AND MEASURES: pages 4-5. "For our categorical measure of deprivation, LAs were assigned to quintiles according to their income deprivation score such that 20% of the 2008 child population was apportioned to each quintile."; "We also used a continuous measure of the income deprivation score, converted to a weighted rank by assigning a value from 0 to 1 based on the midpoint of the LA's range in the cumulative distribution. When using this value as a continuous exposure variable in the regression model, the estimated coefficient expresses the Slope Index of Inequality (SII)"
Statistical methods	12	(a) Describe all statistical methods, including those used to control for confounding.<br>Done. METHODS / STATISTICAL ANALYSIS: page 7
		(*b*) Describe any methods used to examine subgroups and interactions<br>Done. METHODS / STATISTICAL ANALYSIS: page 7 . "We included an interaction between the spline terms for the effect of year and deprivation to allow for potential differences in trend by SEC."
		(*c*) Explain how missing data were addressed<br>Done. SUPPLEMENTARY MATERIALS / APPENDIX 3. "In the data, for reasons of confidentiality, numbers from one to five inclusive were supressed. For each missing value we therefore imputed a random integer in this range. There were only three cases of missing data, across two years, early in the implementation of the CIN census: age stratified data were not available for Havering and Newham in 2012, or Norfolk in 2013. Given the low degree of missingness, we performed complete case analyses."
		(*d*) *Cohort study*—If applicable, explain how loss to follow-up was addressed<br>NA<br>*Case-control study*—If applicable, explain how matching of cases and controls was addressed<br>NA<br>*Cross-sectional study*—If applicable, describe analytical methods taking account of sampling strategy<br>NA
		(e) Describe any sensitivity analyses.<br>NA
**Results**		
Participants	13*	(a) Report numbers of individuals at each stage of study—eg numbers potentially eligible, examined for eligibility, confirmed eligible, included in the study, completing follow-up, and

For peer review only - http://bmjopen.bmj.com/site/about/guidelines.xhtml

		analysed<br>NA
		(b) Give reasons for non-participation at each stage<br>NA
		(c) Consider use of a flow diagram<br>NA
Descriptive data	14*	(a) Give characteristics of study participants (eg demographic, clinical, social) and information on exposures and potential confounders<br>NA
		(b) Indicate number of participants with missing data for each variable of interest<br>Done. SUPPLEMENTARY MATERIALS / APPENDIX 3. "In the data, for reasons of confidentiality, numbers from one to five inclusive were supressed. For each missing value we therefore imputed a random integer in this range. There were only three cases of missing data, across two years, early in the implementation of the CIN census: age stratified data were not available for Havering and Newham in 2012, or Norfolk in 2013. Given the low degree of missingness, we performed complete case analyses."
		(c) *Cohort study*—Summarise follow-up time (eg, average and total amount)<br>NA
Outcome data	15*	*Cohort study*—Report numbers of outcome events or summary measures over time<br>NA
		Case-control study—Report numbers in each exposure category, or summary measures of exposure<br>NA
		Cross-sectional study—Report numbers of outcome events or summary measures<br>NA
Main results	16	(*a*) Give unadjusted estimates and, if applicable, confounder-adjusted estimates and their precision (eg, 95% confidence interval). Make clear which confounders were adjusted for and why they were included.<br>Done. RESULTS: pages 5-8. We have provided data on unadjusted trends in descriptive plots. In the models we have adjusted for unemployment.
		(*b*) Report category boundaries when continuous variables were categorized<br>NA
		(*c*) If relevant, consider translating estimates of relative risk into absolute risk for a meaningful time period<br>Done. RESULTS: pages 5-8. Absolute change in rates reported.
Other analyses	17	Report other analyses done—eg analyses of subgroups and interactions, and sensitivity analyses.<br>Done. SUPPLEMENTARY MATERIALS / APPENDICES 6-7. Age stratified analyses of crude CLA, CPP and CIN rates by LA deprivation quintile; Crude CPP rates by deprivation quintile, stratified by category of abuse
**Discussion**		
Key results	18	Summarise key results with reference to study objectives.<br>Done. DISCUSSION: page 9
Limitations	19	Discuss limitations of the study, taking into account sources of potential bias or imprecision. Discuss both direction and magnitude of any potential bias<br>Done. DISCUSSION: page 9
Interpretation	20	Give a cautious overall interpretation of results considering objectives, limitations,

For peer review only - http://bmjopen.bmj.com/site/about/guidelines.xhtml

		multiplicity of analyses, results from similar studies, and other relevant evidence.<br>Done. DISCUSSION: pages 9-11
Generalisability	21	Discuss the generalisability (external validity) of the study results.<br>Done. DISCUSSION / STRENGTHS AND LIMITATIONS: page 10
**Other information**		
Funding	22	Give the source of funding and the role of the funders for the present study and, if applicable, for the original study on which the present article is based<br>Done. FUNDING SOURCES: page 12.

*Give information separately for cases and controls in case-control studies and, if applicable, for exposed and unexposed groups in cohort and cross-sectional studies.

Note: An Explanation and Elaboration article discusses each checklist item and gives methodological background and published examples of transparent reporting. The STROBE checklist is best used in conjunction with this article (freely available on the Web sites of PLoS Medicine at http://www.plosmedicine.org/, Annals of Internal Medicine at http://www.annals.org/, and Epidemiology at http://www.epidem.com/). Information on the STROBE Initiative is available at www.strobe-statement.org.

For peer review only

For peer review only - http://bmjopen.bmj.com/site/about/guidelines.xhtml

BMJ Open

Trends in inequalities in looked after children in England 2004-2019: a local area ecological analysis

Journal:	*BMJ Open*
Manuscript ID	bmjopen-2020-041774.R1
Article Type:	Original research
Date Submitted by the Author:	10-Aug-2020
Complete List of Authors:	Bennett, Davara; University of Liverpool Faculty of Health and Life Sciences, Department of Public Health and Policy<br>Mason, Kate; University of Liverpool Faculty of Health and Life Sciences, Department of Public Health and Policy<br>Schlüter, Daniela; University of Liverpool Faculty of Health and Life Sciences, Department of Public Health and Policy<br>Wickham, S; University of Liverpool Faculty of Health and Life Sciences, Department of Public Health and Policy<br>Lai, Eric; University of Liverpool Faculty of Health and Life Sciences, Department of Public Health and Policy<br>Alexiou, Alexandros; University of Liverpool Faculty of Health and Life Sciences, Department of Public Health and Policy<br>Barr, Ben ; University of Liverpool Faculty of Health and Life Sciences, Department of Public Health and Policy<br>Taylor-Robinson, David; University of Liverpool Faculty of Health and Life Sciences, Department of Public Health and Policy
**Primary Subject Heading**:	Public health
Secondary Subject Heading:	Epidemiology, Health policy
Keywords:	Child protection < PAEDIATRICS, PUBLIC HEALTH, Community child health < PAEDIATRICS

SCHOLARONE™
Manuscripts

I, the Submitting Author has the right to grant and does grant on behalf of all authors of the Work (as defined in the below author licence), an exclusive licence and/or a non-exclusive licence for contributions from authors who are: i) UK Crown employees; ii) where BMJ has agreed a CC-BY licence shall apply, and/or iii) in accordance with the terms applicable for US Federal Government officers or employees acting as part of their official duties; on a worldwide, perpetual, irrevocable, royalty-free basis to BMJ Publishing Group Ltd ("BMJ") its licensees and where the relevant Journal is co-owned by BMJ to the co-owners of the Journal, to publish the Work in this journal and any other BMJ products and to exploit all rights, as set out in our licence.

The Submitting Author accepts and understands that any supply made under these terms is made by BMJ to the Submitting Author unless you are acting as an employee on behalf of your employer or a postgraduate student of an affiliated institution which is paying any applicable article publishing charge ("APC") for Open Access articles. Where the Submitting Author wishes to make the Work available on an Open Access basis (and intends to pay the relevant APC), the terms of reuse of such Open Access shall be governed by a Creative Commons licence – details of these licences and which Creative Commons licence will apply to this Work are set out in our licence referred to above.

Other than as permitted in any relevant BMJ Author's Self Archiving Policies, I confirm this Work has not been accepted for publication elsewhere, is not being considered for publication elsewhere and does not duplicate material already published. I confirm all authors consent to publication of this Work and authorise the granting of this licence.

For peer review only

TITLE PAGE

Trends in inequalities in looked after children in England 2004-2019: a local area ecological analysis

Davara L Bennett[1] MSc, Kate E Mason[1] PhD, Daniela K Schlüter[1] PhD, Sophie Wickham[1] PhD, Eric TC Lai[1] PhD, Alexandros Alexiou[1] PhD, Ben Barr[1*] PhD, David Taylor-Robinson[1*] PhD

1. University of Liverpool, Faculty of Health and Life Sciences, Department of Public Health and Policy.

- Joint Senior Author

Correspondence to: Davara L Bennett, University of Liverpool, Waterhouse Building 2nd Floor Block F, L69 3GL, Liverpool, United Kingdom

E-mail: davara.bennett@liverpool.ac.uk

Word Count: 3,638

For peer review only

ABSTRACT

Objective: To determine whether there were inequalities in the precipitous rise in rates of children becoming looked after (CLA) in England over the last decade.

Setting: 150 English upper-tier local authorities.

Participants: Children under the age of 18.

Primary outcome measure: The annual age-standardised rate of children becoming looked after (CLA rate) across English local authorities, grouped into quintiles based on their level of income deprivation. Slope indices of inequality (SII) were estimated using longitudinal segmented mixed effects models, controlling for unemployment, a marker of the recession.

Results: Since 2008, CLA rates have risen dramatically, with a greater rise in the most disadvantaged quintiles. Between 2007 and 2019 the gap between most and least deprived areas increased by 15 children per 100,000 per year (95% CI 4-26) relative to the previous trend, and after controlling for local economic trends. The financial recession was independently associated with rising CLA rates. For each percentage point increase in the unemployment rate, an estimated additional 9 children per 100,000 (95% CI 6-11) per year became looked after the following year.

Conclusions: The dramatic rise in the numbers of CLA has been greater in poorer areas, and in areas more deeply affected by the recession. Policies should urgently address inequalities in the determinants of children becoming looked after at local area level.

For peer review only

STRENGTHS AND LIMITATIONS OF THE STUDY

- This study is the first to quantify inequalities in child welfare outcomes in England longitudinally, using segmented mixed effects models to show that the gap in rates of children entering care between most and least deprived areas is on the rise.
- The study uses routinely available data for the whole of England, and explores several child welfare outcomes in order to describe trends throughout the child welfare system.
- An important limitation is that, using an ecological area-level analysis, we cannot conclude that children entering care were directly affected by the exposures of interest.

For peer review only

INTRODUCTION

Improving the health outcomes and life chances of children looked after (CLA) is a matter of public health concern (1). In England, over the last decade, the prevalence of CLA increased dramatically, from 53 to 64 per 10,000 children, a rise of 20 percent. At last count, in March 2019, their number exceeded 78,000 (2). The health outcomes and life chances of these children, many of whom have experienced abuse, neglect and other forms of acute adversity, may differ markedly from those of their peers. On average, individuals who have been looked after face worse outcomes across a range of measures, throughout the life-course – physical and mental health, education, offending, employment, income – relative to those who have not come in contact with child welfare services. (3)

Reducing the economic burden associated with the consequences of CLA is of particular concern to policymakers: supporting CLA represents a major expenditure at local authority (LA) level. Across England, between 2011 and 2018, CLA spend increased by £1.9bn in real terms, to £4.6bn. Children's services have been described as approaching breaking point (4). Internationally, there have been increasing calls for a preventative approach to CLA that addresses upstream risk factors for child abuse and neglect (5).

A number of factors may have contributed to rising CLA rates in England over the last decade. High profile serious case reviews (6), shifting understanding of the impact of different forms of childhood adversity (7), and legal judgements clarifying LA statutory responsibilities (8), may all affect thresholds for child welfare intervention. Wider economic changes may also underlie trends in CLA rates. Growing up in adverse socioeconomic circumstances (SECs) is an important risk factor for child abuse and neglect and for children being taken into care (9), with poverty, unemployment and parental financial stress recognised as contributory causal factors (10,11). Several experimental and quasi-experimental studies from the US have shown that raising family income and reducing poverty leads to a reduction in rates of child abuse and neglect (10,12).

In 2008, the onset of the financial recession led to rising unemployment in England, and to fiscal policy with far-reaching social consequences. In 2010 the UK government began introducing a series of austerity measures with the stated intention of eliminating the budget deficit and reducing the national debt (13). The welfare system has been a principal focus of cuts and reforms (14). These have adversely affected, in particular, families with children and those at greatest risk of poverty, fuelling a rise in child poverty (15). At the same time,

For peer review only

regressive cuts to LA budgets have led to reduced spending on early childhood education and care, and other prevention services (16). Whilst increases in unemployment during the recession were dispersed across all parts of the country, changes in welfare provision and cuts to prevention have disproportionately affected deprived areas (17). If these changes are leading to increased incidence of child abuse and neglect, we would expect CLA rates to rise more rapidly in more deprived areas.

There are stark differences in rates of CLA across LAs in England (1). Less clear is how these are changing over time. Our aim in this study is to determine whether the rate of children becoming looked after increased more in deprived areas of the country. We further quantify trends in inequalities in children experiencing other forms of child welfare intervention, in order to assess whether findings for CLA are consistent across child welfare outcomes.

METHODS

Data sources and measures

We undertook segmented longitudinal analysis of CLA rates at local authority level. We used routinely available data from 150 English upper-tier LAs between 2004 and 2019, based on 2010 boundaries (see appendix 1). Two LAs, the City of London and the Isles of Scilly, were excluded due to their small population size.

Our primary outcome of interest was the annual age-standardised rate of children becoming looked after by LAs in England (hereafter referred to as 'CLA rate'). Panel data for the number of CLA, by age group, were drawn from the 'children looked after data return', submitted by LAs to the Department for Education on 31st March annually (2). We refer to the financial year by the latter year throughout. Direct age standardisation was performed using the national population distribution of children.

Secondary outcomes captured the wider population of children known to children's social care. Figure 1 outlines the different child welfare outcomes. The system has been likened to a 'funnel', with a progressively smaller number of children experiencing increasingly acute interventions. We used the annual age standardised rate of children becoming the subject of a Child Protection Plan ('CPP rate'), and children beginning an 'episode of need' ('CIN rate'). Data for these outcomes between 2010 and 2019 were sourced from the Children in Need (CIN) Census records of children referred for social care support

For peer review only

in England (18). For children on a CPP, a breakdown of numbers by category of abuse were available. Disaggregation by age group was requested via a Freedom of Information request, and obtained for years 2012 to 2019.

As a measure of SEC, we used the income deprivation score of the 2010 Indices of Multiple Deprivation (19). This is a non-overlapping count of individuals who, as a result of low earnings, qualify for means-tested benefits, as a proportion of the total population (20). We used 2010 scores based on 2008 data, collected prior to the implementation of austerity policies, to avoid conflating the time-invariant measure of deprivation with unmeasured time-varying exposures that may be changing in response to austerity policies, and so contributing to changing inequalities. In descriptive analyses, we categorised the income deprivation score, assigning LAs to quintiles such that 20% of the 2008 child population was apportioned to each quintile. In regression models, we used a continuous measure of the income deprivation score, converted to a weighted rank by assigning a value from 0 to 1 based on the midpoint of the LA's range in the cumulative distribution. When using this value as a continuous exposure variable in the regression model, the estimated coefficient expresses the change in the Slope Index of Inequality (SII), a commonly used indicator of the association between health outcomes and socioeconomic deprivation (21). The same value can be used to derive the change in the Relative Index of Inequality (RII) when the outcome variable in the regression model is log-transformed and the estimated coefficient exponentiated. In our statistical analyses, the SII represents the absolute difference, and the RII the relative difference, in child welfare outcomes between the LA of lowest and highest level of income deprivation, taking into account the distribution of the child population across LAs (22).

Our analyses also included LA unemployment rates as a covariate in order to separate out the impact of the recession on child welfare outcomes, and so determine whether changes in inequalities were independent of the effects of unemployment. We used data on the number of people claiming Jobseeker's Allowance, plus those claiming Universal Credit who are out of work, as a proportion of residents aged 16-64, in the financial year(23). Although the measure does not capture all unemployment, it is precise and stable at local-area level, is highly correlated with survey-based measures of unemployment (24), and spans the time period of interest. Since the effects of unemployment on child welfare outcomes are unlikely to be immediate, we lagged the variable by one year.

Statistical analysis

For peer review only

First, we assessed descriptive trends for our outcome CLA rate, across LAs grouped into quintiles of income deprivation, between 2004 and 2019. Second, we estimated a segmented linear regression model, with: age-standardised CLA rate as the outcome; year, unemployment rate and income deprivation weighted rank as continuous independent variables; and random intercept and slope terms to account for the correlation between measurements within LAs. Based on our initial descriptive analysis, we included a linear spline for the effect of calendar year, with one knot indicating the timing of the change in trend. We used an iterative search procedure to confirm the knot position resulting in the model with the smallest Bayesian Information Criterion value (25,26). We included an interaction between the spline terms for the effect of year and deprivation to allow for potential differences in trend by SEC. Full details are provided in appendices 2-3.

We used this model to assess whether there was a significant change in the trend in CLA over this period, whether this differed by level of LA income deprivation, and the potential contribution of unemployment to trends in our outcome. We estimated all model parameters by maximum likelihood, using generalized likelihood ratio statistics to compare nested models, and Wald statistics to test hypotheses about model parameters. Similar models were fitted for each of our secondary outcomes, CPP and CIN rates, across years for which data were available, 2012-2019 – based on our descriptive analysis no linear splines were included in these models. Models were estimated using the lme4 package (27), in R version 3.5.1. We carried out supplementary analyses, assessing descriptive trends for all outcomes stratified by age, and, for CPP, by category of abuse (appendices 4-5), and making predictions based on the model (appendix 6). Finally, we fit a model with log-transformed values of the age standardised CLA rate as the outcome in order to derive the RII, and assess trends in relative, as well as absolute inequalities (appendix 7).

Patient and Public Involvement

The research question was informed by early conversations with policymakers and practitioners in the Merseyside area, and reflects the evidence needs identified by senior leaders within Children's Social Care in a priority-mapping exercise facilitated by the What Works Centre for Children's Social Care (28). Early plots were shared with local contacts, and the ensuing discussions informed our hypotheses about drivers of recent trends, in particular age-stratified trends. These hypotheses have informed our research agenda.

RESULTS

For peer review only

Trends in child welfare outcomes

Figure 2 shows CLA rate, by LA income deprivation quintile. Between 2004 and 2008, overall CLA rates dipped slightly: a small increase in the most affluent quintile was offset by decreases in more deprived areas. In 2008, the absolute difference in CLA rate between most and least deprived quintiles was 144 per 100,000 (95% CI 104-184). From around 2008, there was a change in trend and CLA rates rose. A social gradient in CLA is apparent throughout, with the absolute difference between most and least deprived quintiles rising to 174 per 100,000 (95% CI 127-221) in 2019, an increase of 21% from 2008.

Figure 3 shows the CPP and CIN rates. As with CLA rates, CPP rates have risen since 2012, and show a clear social gradient. However, the increase occurred relatively evenly across all groups of LAs, in all age groups. CIN rates also exhibit a social gradient, but trends appear to be relatively stable over time.

Supplementary analyses (appendix 4, appendix figures 1-3) show that the gap in CLA rates between most and least deprived quintiles differed by age. The gap is wide, but relatively stable over time in the youngest age group, children under 1. The gap is widening in the oldest age group, those aged 16-17. Finally, stratifying CPP rates by category of abuse complicates the overall picture of an even rise in rates across all LA income quintiles: we uncovered a widening gap between most and least deprived areas in rates of children becoming subject to a CPP due to concerns about emotional abuse (appendix 5, appendix figure 4).

Segmented linear regression models

Tables 1-2 summarise the results of the segmented regression analyses. For full model output and residual diagnostics, see appendices 7-8 (appendix figures 5-16). For CLA, a knot in 2007, ahead of the 2008 change in trend identified in our descriptive analysis, resulted in the best model fit, indicating a change in trend at this point (appendix 3, appendix figure 17). In our model, rising unemployment in the wake of the financial recession was independently associated with rising CLA rates: for each percentage point increase in the unemployment rate, an estimated additional 9 children per 100,000 (95% CI 6-11) per year entered care the following year. There were no associations between CPP and CIN rates and unemployment rates.

For peer review only

Table 1: Association between outcomes and unemployment rate

Outcome and time period	Annual change (in children per 100,000) for a 1% increase in the unemployment rate the previous year [95% CI]
**CLA rate,** 2004-19	9.0 [6.5, 11.4]
**CPP rate,** 2012-19	-10.4 [-22.2, 1.4]
**CIN rate,** 2012-19	68.5 [-3.1, 140.1]

But unemployment rates do not account for differences in trends between more and less deprived LAs. In 2004, after controlling for LA unemployment, the SII was 193. This captures the absolute inequalities gap across the distribution of LAs on the basis of area deprivation, indicating that there were 193 more CLA per 100,000 in the most deprived LA, compared to the least deprived (95% CI 140-246). Between 2004 and 2007, this gap declined by 11 children per 100,000 per year (95% CI 0-22) (table 2). From 2007 there was a significant change in the trend in inequalities: the gap increased by 15 children per 100,000 per year (95% CI 4-26) relative to the previous trend. Relative inequalities follow the same trend (appendix 7). Altogether, based on our model, we estimate that an additional 18,567 (95% CI 3,553 - 33,394) children were taken into care between 2007 and 2019, than would have been expected had the rise from 2007 occurred in more deprived LAs as it did in the median LA (appendix 6, appendix figure 18).

Table 2: Trends in the Slope Index of Inequality across child welfare outcomes

Outcome and time period	Annual change (in children per 100,000) in the Slope Index of Inequality [95% CI]
**CLA**	
2004-07	-11.4 [-22.3, -0.5]
2007-19, relative to previous trend	14.9 [3.6, 26.2]
**CPP**	
2012-19	4.4 [-11.2, 20.0]
**CIN**	
2012-19	47.1 [-62.7, 156.9]

DISCUSSION

Main findings

For peer review only

The dramatic rise in CLA in England since 2008 has been greater in poorer areas of the country, increasing inequalities. Overall an additional 18,567 (95% CI 3,553 - 33,394) children were taken into LA care between 2007 and 2019 than would be expected if the rise from 2007 had occurred more evenly across LAs. These findings cannot be explained by local economic trends, and are consistent with our hypothesis that austerity measures may have contributed to rising rates of child welfare outcomes. Our analysis also shows that the rise in CLA was associated with rising unemployment at LA level, a marker of the recession.

Trends in inequalities in CLA are not simply mirroring broader trends throughout the 'funnel' of children's social care. Whilst CPP rates are also rising, and show a clear social gradient, we did not find a greater increase in more deprived compared to less deprived areas for children becoming the subject of a CPP and beginning an episode of need.

Several studies have described trends in child welfare outcomes or child maltreatment in the UK. These support our finding of a change in trend and rising rates from around 2007-08 (29) and add context, demonstrating that the turn has followed a thirty-year decline in overall rates – though the rise in CPPs due to neglect and emotional abuse have been rising since the 1990s (30). However, to our knowledge no studies have yet documented trends in inequalities. Paul Bywaters and colleagues at the Child Welfare Inequalities Project began producing evidence of persistent and systematic inequalities in child welfare outcomes in the UK beginning in 2015 (3). This longitudinal analysis of inequalities is indebted to their work.

Strengths and limitations

This study is the first to quantify inequalities in child welfare outcomes longitudinally. A strength is that it uses routinely available data for the whole of England, and explores several child welfare outcomes in order to describe trends throughout the child welfare system.

There are several important study limitations. Due to the lack of individual level data, we used an ecological area-level analysis, and cannot identify whether children entering care were directly affected by income deprivation and unemployment. Conceptually, our portrayal of children's social care as a funnel reflects a theoretical model of how a well-functioning system might operate (figure 1), and may not reflect the trajectory of many individual children and families experiencing child welfare intervention. The association between income deprivation and unemployment rates and child welfare outcomes in our analysis may be due to trends in unobserved time-varying confounding factors that varied between LAs.

For peer review only

Trends in the data reflect the interaction between underlying need and children's services response and we interpret our findings in this light, with caution. Previous analyses by Bywater and colleagues demonstrated the existence of an "inverse intervention law" in child welfare outcomes: a greater risk of intervention in affluent compared to deprived LAs for the same level of neighbourhood deprivation (31), despite lower overall intervention rates. Our models at the level of LAs do not account for the inverse intervention law or rising thresholds reported in more deprived areas. However, this must add weight to our findings: insofar as they reflect changing underlying need, our estimates of the SII are likely to be highly conservative.

Potential explanations of our findings

Changing thresholds

Several changes during this time period may have influenced thresholds for intervention. Firstly, the death by violence of baby Peter Connelly occurred in 2007, when we see a change in the trend of CLA in our data (32). Media and political narratives that emerged in the aftermath of his death centred on the failure of children's services to intervene (33), and ensuing reports by The Children and Family Court Advisory and Support Service note a 'Baby P effect', a marked, short-term rise in applications for care orders in a risk-averse environment (6). This likely accounts for some of the change in trend and initial rise in CLA rates from 2007. Others have argued that a greater policy focus on early intervention and adoption in order to improve outcomes for children experiencing adversity has led to a more interventionist, less family-oriented approach (34). Secondly, in 2009 the Southwark Judgement clarified and reinforced LAs' statutory duties in relation to 16-17 year olds presenting to the LA as homeless (8). This, together with a general shift in practice towards regarding adolescents as vulnerable children rather than nascent adults (35), and greater awareness of extra-familial forms of abuse and principles of contextual safeguarding (36), may be contributing to the rising rates of 16-17 year olds across all outcomes. However, these events are unlikely to fully explain the long-term rise in CLA rates disproportionately affecting more deprived areas.

Economic trends

We found evidence of a positive association between unemployment and CLA rates. Though evidence from the UK is scarce, this aligns with Gillham et al.'s finding of a correlation between male unemployment and child physical abuse in Scotland in the early 1990s (37) and

For peer review only

more recent and extensive evidence from the US demonstrating an association between the recession and increased risk of abuse (38–40). The family stress model posits that heightened stress due to adverse SECs may erode mental health and strain domestic relationships, leading to negative parenting behaviours and increased risk of child abuse and neglect. Barr et al.'s study of the mental health impact of the recession lends credence to this theorised mechanism, demonstrating an association between unemployment and mental health problems in the UK over the same period(17). Yet unemployment did not fully explain changes in CLA rates in our analysis, and unemployment rates have fallen rapidly since 2012: unemployment cannot explain the continued increase in CLA after 2012, nor does it explain rising inequalities. Austerity policies subsequent to the initial recession "shock" may have compounded poor outcomes, affecting inequalities in CLA in several ways.

Changes to welfare provision and prevention

Regressive cuts to English LA budgets, with deeper cuts in more deprived areas, have precipitated a shift in expenditure away from prevention towards acute services(16). Between 2011 and 2018, spending on CLA increased by 68% in real terms, whereas spending on early years preventative services (including Sure Start) and non-statutory young people's services fell about 21%. Reports of rising thresholds for early help in more resource constrained settings, have raised concerns that we are 'storing up trouble' for the future(41). A surge in children entering care who might have benefited from early support could explain the greater rise in more deprived LAs. Adolescents may be particularly susceptible to the consequences of austerity, exposed as they are on multiple fronts, not just in the household and schools, but increasingly in the wider community. Combined cuts to welfare benefits, youth services(42), children's mental health services (43), and community policing(44), might disproportionately affect adolescents in more deprived areas, contributing to widening inequalities in this age group.

Changes to welfare benefits have led to rising child poverty, a contributory causal factor in child abuse and neglect (10,15). Averages losses in earning were particularly high in the more deprived West Midlands and the North West (15). The most vulnerable children on the edge of care, living in families already struggling to cope, may be particularly sensitive to changes in welfare benefit provision. In particular, the phased introduction of Universal Credit from 2013, with its monthly payments in arrears, enhanced conditionality and punitive sanctions, may have compounded financial stress (15) and parental mental health (45). This

For peer review only

would increasingly lead to more children entering care in deprived areas, contributing to trends in inequalities uncovered in our study. Further research is needed to investigate the impact of changing LA prevention spend and child poverty on child welfare outcomes.

Policy and practice implications

We demonstrate that the increase in CLA rates from 2007 has been greater in more deprived LAs. Although it is not possible to say what constitutes an appropriate CLA rate (46), a differential rise by LA deprivation that cannot be explained by the recession is consistent with an increase in underlying need fuelled by welfare changes and cuts to prevention services. While anti-poverty social work practice has a crucial role to play in safely reducing CLA rates and inequalities (47), this must be supported by wider policies to address the social conditions of children's lives. At the national level, this must begin with a renewed commitment to ending child poverty. Tightened social security for families with children, and increased funding for LA Children's Services, are safeguarding priorities. At the local level, holding the line on prevention services, amidst statutory pressures, may yield long-term social and economic benefits. Investment in children is key.

For peer review only

Figure legends

Manuscript figures

Figure 1: Description of the Children's Social Care System in England

Figure 2: CLA rates by LA income deprivation quintile, 2004-2019, with 95% CIs

Figure 3: CPP and CIN rates by LA income deprivation quintile, 2012-19, with 95% CIs

Appendix figures

Appendix figure 1: CLA rates by LA income deprivation quintile, stratified by age

Appendix figure 2: CPP rates by LA income deprivation quintile, stratified by age

Appendix figure 3: CIN rates by LA income deprivation quintile, stratified by age

Appendix figure 4: CPP rates by LA income deprivation quintile, stratified by category of abuse

Appendix figure 5: CLA model - absolute inequalities - histogram of standardised residuals

Appendix figure 6: CLA model - absolute inequalities - quantile quantile plot

Appendix figure 7: CLA model - absolute inequalities - comparing observed and predicted rates

Appendix figure 8: CLA model - relative inequalities - histogram of standardised residuals

Appendix figure 9: CLA model - relative inequalities - quantile quantile plot

Appendix figure 10: CLA model - relative inequalities - comparing observed and predicted rates

Appendix figure 11: CPP model - histogram of standardised residuals

Appendix figure 12: CPP model - quantile quantile plot

Appendix figure 13: CPP model - comparing observed and predicted rates

Appendix figure 14: CIN model - histogram of standardised residuals

Appendix figure 15: CIN model - quantile quantile plot

Appendix figure 16: CIN model - comparing observed and predicted rates

For peer review only

Appendix figure 17: CLA model - breakpoint analysis

Appendix figure 18: CLA model - predictions based on the model

For peer review only

Funding and declarations of interest

DB, DT-R, BB, KM and AA are funded by the National Institute for Health Research School for Public Health Research. SW is funded by a Wellcome Trust Society and Ethics Research Fellowship (200335/Z/15/Z). DT-R and ETCL are funded by the Medical Research Council (MRC) on a Clinician Scientist Fellowship (MR/P008577/1). BB is also supported by the National Institute for Health Research Applied Research Collaboration North West Coast (ARC NWC). The views expressed in this publication are those of the authors and not necessarily those of the National Institute for Health Research or the Department of Health and Social Care, MRC or Wellcome Trust. The funders of the study had no role in study design, data collection, data analysis, data interpretation, or the writing of the report. The corresponding author had access to all of the data in the study and had final responsibility for the decision to submit for publication. We declare no competing interests.

Data sharing and ethics

The publicly available, aggregate data can be accessed from the Place-based Longitudinal Data Resource (PLDR) (48), and the code is available upon reasonable request. Research Ethics committee approval was not required for secondary data analysis.

Contributorship statement

DB is lead author and guarantor. DT-R and BB are joint senior author. DB and DT-R planned the study. DB, BB and DT-R analysed the data, supported by KM, DS, SW, ET and AA. DB, DT-R and BB led the drafting and revision of the manuscript. All authors contributed to the interpretation of the data and revision of the manuscript; all authors approved the submitted version of the manuscript. We confirm that authors have no conflicts of interest to disclose.

License statement

I, the Submitting Author has the right to grant and does grant on behalf of all authors of the Work (as defined in the below author licence), an exclusive licence and/or a non-exclusive licence for contributions from authors who are: i) UK Crown employees; ii) where BMJ has agreed a CC-BY licence shall apply, and/or iii) in accordance with the terms applicable for US Federal Government officers or employees acting as part of their official duties; on a worldwide, perpetual, irrevocable, royalty-free basis to BMJ Publishing Group Ltd ("BMJ") its licensees and where the relevant Journal is co-owned by BMJ to the co-owners of the

For peer review only

Journal, to publish the Work in Journal of Epidemiology & Community Health and any other BMJ products and to exploit all rights, as set out in our licence.

The Submitting Author accepts and understands that any supply made under these terms is made by BMJ to the Submitting Author unless you are acting as an employee on behalf of your employer or a postgraduate student of an affiliated institution which is paying any applicable article publishing charge ("APC") for Open Access articles. Where the Submitting Author wishes to make the Work available on an Open Access basis (and intends to pay the relevant APC), the terms of reuse of such Open Access shall be governed by a Creative Commons licence – details of these licences and which Creative Commons licence will apply to this Work are set out in our licence referred to above.

For peer review only

REFERENCES

1. Bywaters P. Inequalities in child welfare: Towards a new policy, research and action agenda. British Journal of Social Work. 2015;45(1):6–23.
2. Department for Education. Statistics: looked-after children (Collection) [Internet]. 2013 [cited 2020 Jun 16]. Available from: https://www.gov.uk/government/collections/statistics-looked-after-children
3. Simkiss D. Outcomes for looked after children and young people. Paediatrics and Child Health (United Kingdom). 2012;22(9):388–92.
4. Local Government Association. LGA Budget Submission: Autumn 2017. 2017.
5. O'Donnell M, Scott D, Stanley F. Child abuse and neglect - Is it time for public health approach? Australian and New Zealand Journal of Public Health. 2008;32(4):325–30.
6. CAFCASS. Annual report and accounts 2009-10. Crown; 2010.
7. Featherstone B, Gupta A, Morris K, Warner J. Let's stop feeding the risk monster: Towards a social model of 'child protection.' Families, Relationships and Societies. 2018;7(1):7–22.
8. R (on the application of G) v Southwark London Borough Council 2009.
9. Walsh D, McCartney G, Smith M, Armour G. Relationship between childhood socioeconomic position and adverse childhood experiences (ACEs): A systematic review. Vol. 73, Journal of Epidemiology and Community Health. BMJ Publishing Group; 2019. p. 1087–93.
10. Bywaters P, Bunting L, Davidson G, Hanratty J, Mason W, McCartan C, et al. The relationship between poverty, child abuse and neglect: an evidence review. 2016.
11. Hillson JMC, Kuiper NA. A stress and coping model of child maltreatment. Clinical Psychology Review. 1994;14(4):261–85.
12. Cancian M, Yang MY, Slack KS. The effect of additional child support income on the risk of child maltreatment. Social Service Review. 2013;87(3):417–37.
13. HM Treasury. Budget 2010. London; 2010.
14. Department for Work & Pensions. 2010 to 2015 government policy: welfare reform - GOV.UK [Internet]. 2010 [cited 2020 Jun 16]. Available from: https://www.gov.uk/government/publications/2010-to-2015-government-policy-welfare-reform/2010-to-2015-government-policy-welfare-reform
15. Tucker J. The Austerity Generation: the impact of a decade of cuts on family incomes and child poverty [Internet]. Child Poverty Action Group. 2017. Available from: http://www.cpag.org.uk/sites/default/files/Austerity Generation FINAL.pdf
16. Webb CJR, Bywaters P. Austerity, rationing and inequity: trends in children's and young peoples' services expenditure in England between 2010 and 2015. Local Government Studies. 2018 May 4;44(3):391–415.
17. Barr B, Kinderman P, Whitehead M. Trends in mental health inequalities in England during a period of recession, austerity and welfare reform 2004 to 2013. Social Science and Medicine. 2015;147:324–31.
18. Emmott EH, Jay MA, Woodman J. Cohort profile: Children in Need Census (CIN) records of children referred for social care support in England. BMJ Open. 2019;9(2).

For peer review only

19. Ministry of Housing Communities & Local Government. English Indices of Deprivation 2010 (Official Statistics).

20. Department for Communities and Local Government. The English Indices of Deprivation 2015: Technical report. 2015.

21. Straatmann VS, Lai E, Lange T, Campbell MC, Wickham S, Andersen AMN, et al. How do early-life factors explain social inequalities in adolescent mental health? Findings from the UK Millennium Cohort Study. Journal of Epidemiology and Community Health. 2019;73(11):1049–60.

22. Regidor E. Measures of health inequalities: Part 2. Journal of Epidemiology and Community Health. 2004;58(11):900–3.

23. ONS. NOMIS - official labour market statistics 2019.

24. Barr B, Taylor-Robinson D, Scott-Samuel A, McKee M, Stuckler D. Suicides associated with the 2008-10 economic recession in England: Time trend analysis. BMJ (Online) [Internet]. 2012 Sep 8 [cited 2020 Jul 30];345(7873). Available from: https://www.bmj.com/content/345/bmj.e5142

25. Taylor-Robinson D, Lai ETC, Wickham S, Rose T, Norman P, Bambra C, et al. Assessing the impact of rising child poverty on the unprecedented rise in infant mortality in England, 2000-2017: time trend analysis. BMJ Open. 2019;9(10).

26. Barr B, Higgerson J, Whitehead M. Investigating the impact of the English health inequalities strategy: Time trend analysis. BMJ (Online). 2017;358.

27. Bates D, Mächler M, Bolker BM, Walker SC. Fitting linear mixed-effects models using lme4. Journal of Statistical Software. 2015;67(1).

28. Jones L, Scholten C, Whelan E, Clayton V, Sanders M. What Matters For What Works?: Setting research priorities for what works for Children's Social care. 2020.

29. Chandan JS, Gokhale KM, Bradbury-Jones C, Nirantharakumar K, Bandyopadhyay S, Taylor J. Exploration of trends in the incidence and prevalence of childhood maltreatment and domestic abuse recording in UK primary care: a retrospective cohort study using "the health improvement network" database. BMJ open. 2020;

30. Degli Esposti M, Humphreys DK, Jenkins BM, Gasparrini A, Pooley S, Eisner M, et al. Long-term trends in child maltreatment in England and Wales, 1858–2016: an observational, time-series analysis. The Lancet Public Health. 2019 Mar 1;4(3):e148–58.

31. Bywaters P, Brady G, Sparks T, Bos E, Bunting L, Daniel B, et al. Exploring inequities in child welfare and child protection services: Explaining the "inverse intervention law." Children and Youth Services Review. 2015;57:98–105.

32. Haringey Local Safeguarding Children Board. Serious Case Review "Child A." 2009.

33. Warner J. "Heads Must Roll"? Emotional Politics, the Press and the Death of Baby P. British Journal of Social Work. 2014;44(6):1637–53.

34. Featherstone B, Morris K, White S. A marriage made in hell: Early intervention meets child protection. British Journal of Social Work [Internet]. 2014 [cited 2020 Jun 19];44(7):1735–49. Available from: https://academic.oup.com/bjsw/article-abstract/44/7/1735/1718126

35. NSPCC. Teenagers: learning from case reviews. 2014.

For peer review only

36. Lloyd J, Firmin C. No Further Action: Contextualising Social Care Decisions for Children Victimised in Extra-Familial Settings. Youth Justice. 2020;20(1–2):79–92.

37. Gillham B, Tanner G, Cheyne B, Freeman I, Rooney M, Lambie A. Unemployment rates, single parent density, and indices of child poverty: Their relationship to different categories of child abuse and neglect. Child Abuse and Neglect. 1998;22(2):79–90.

38. Brooks-Gunn J, Schneider W, Waldfogel J. The great recession and the risk for child maltreatment. Child Abuse and Neglect. 2013;37(10):721–9.

39. Cherry R, Wang C. The link between male employment and child maltreatment in the U.S., 2000-2012. Children and Youth Services Review. 2016;66:117–22.

40. Millett L, Lanier P, Drake B. Are economic trends associated with child maltreatment? Preliminary results from the recent recession using state level data. Children and Youth Services Review. 2011 Jul 1;33(7):1280–7.

41. All Party Parliamentary Group for Children. Storing Up Trouble: a postcode lottery of children's social care. 2018.

42. Kelly E, Lee T, Sibieta L, Waters T. Public Spending on Children: 2000 to 2020. 2018.

43. Children's Commissioner. Briefing: Children's Mental Healthcare in England. 2017.

44. Allen G, Audickas L. Briefing paper: Police Service Strength [Internet]. 2020. Available from: http://researchbriefings.files.parliament.uk/documents/SN00634/SN00634.pdf

45. Wickham S, Bentley L, Rose T, Whitehead M, Taylor-Robinson D, Barr B. Effects on mental health of a UK welfare reform, Universal Credit: a longitudinal controlled study. The Lancet Public Health. 2020;5(3):e157–64.

46. Bywaters P, Scourfield J, Jones C, Sparks T, Elliott M, Hooper J, et al. Child welfare inequalities in the four nations of the UK. Journal of Social Work. 2020;20(2):193–215.

47. Morris K, Mason W, Bywaters P, Featherstone B, Daniel B, Brady G, et al. Social work, poverty, and child welfare interventions. Child and Family Social Work. 2018;23(3):364–72.

48. Place-based Longitudinal Data Resource [Internet]. [cited 2020 Jun 16]. Available from: https://pldr.org/

For peer review only

The children's social care system has been described as series of 'filters and funnels'[∞]. Using this analogy, through the funnel, successive phases of risk assessment and service response determine a child's incident status:

1. **Referrals**. At the wide end of the funnel are all referrals.
2. **Children in Need**. A child is 'in need' (CIN), if deemed to require additional support in order to achieve a reasonable standard of health and development. An episode of need relates to an open case following a LA's acceptance of a referral to children's social care.
3. **Child Protection Plan**. A Child Protection Plan (CPP) may be drawn up where, following an investigation, concerns persist as to whether a child is suffering, or likely to suffer, significant harm.
4. **Children Looked After** (CLA) are at the narrow end of the funnel, enduring adversity sufficiently severe for the State to intervene in their upbringing[£].

This figure represents the 'funnel' of children's social care, and shows the overlap between incident child welfare status. Size and overlaps are not to scale. Child population estimates are taken from most recent data returns, for the period 2018-19. In rare cases, due to residual safeguarding concerns, a child in care may also be subject to a CPP.

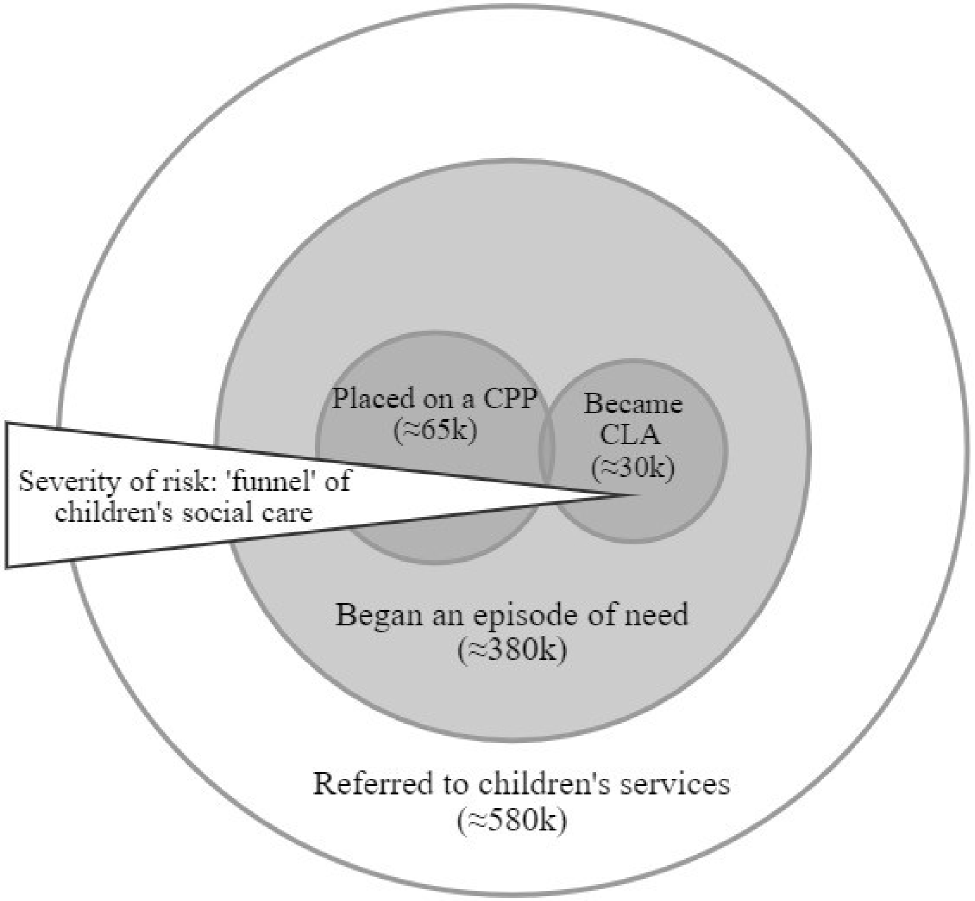

∞ Gibbons J, Bell C, Conroy S. Operating the child protection system: a study of child protection practices in English local authorities. London: HMSO; 1995.
£ Emmott EH, Jay MA, Woodman J. Cohort profile: Children in Need Census (CIN) records of children referred for social care support in England. BMJ Open. 2019;9(2):e023771.

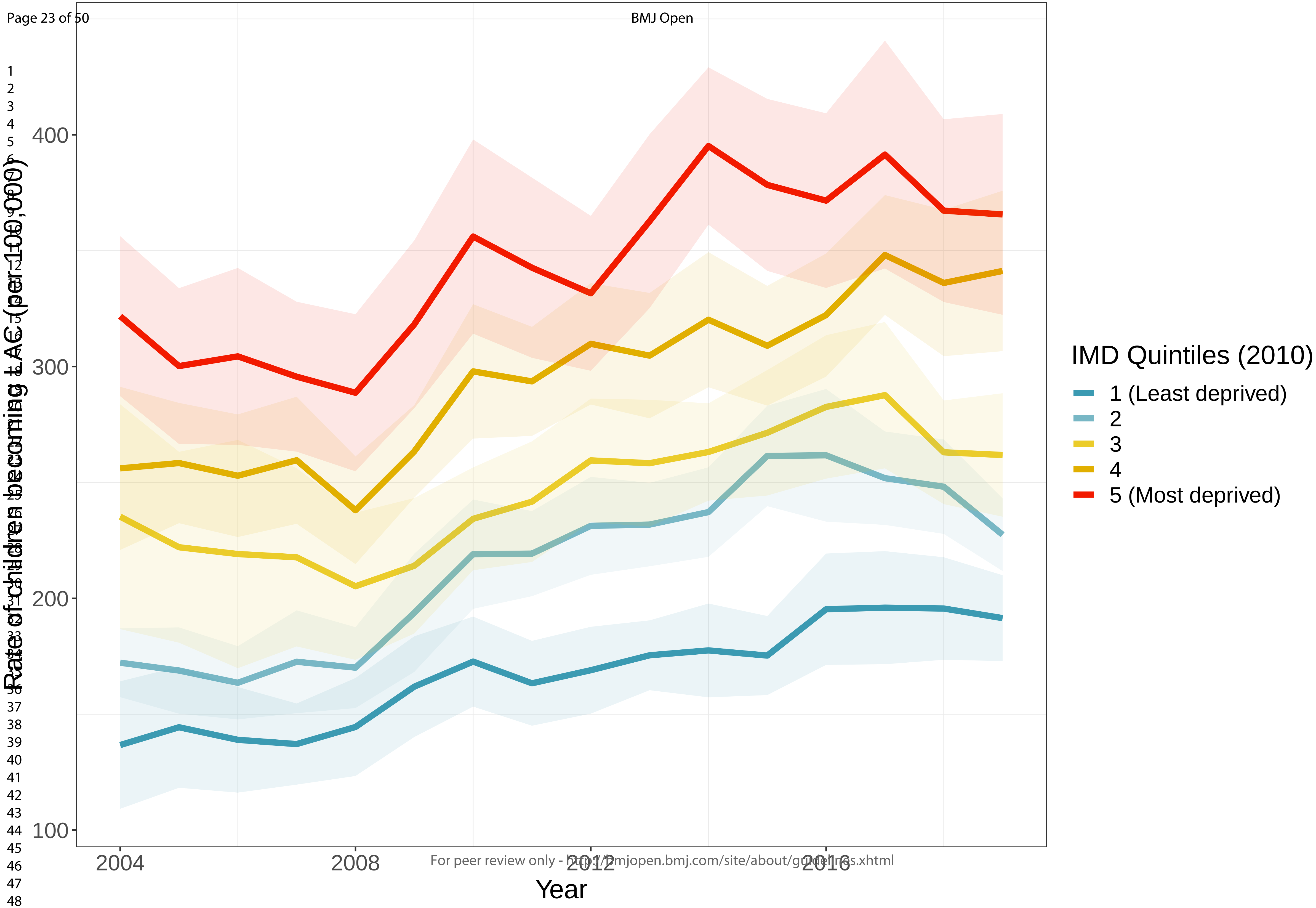
Rate of children becoming LAC (per 100,000)
400
300
200
100
2004
2008
2012
2016
Year
IMD Quintiles (2010)
1 (Least deprived)
2
3
4
5 (Most deprived)

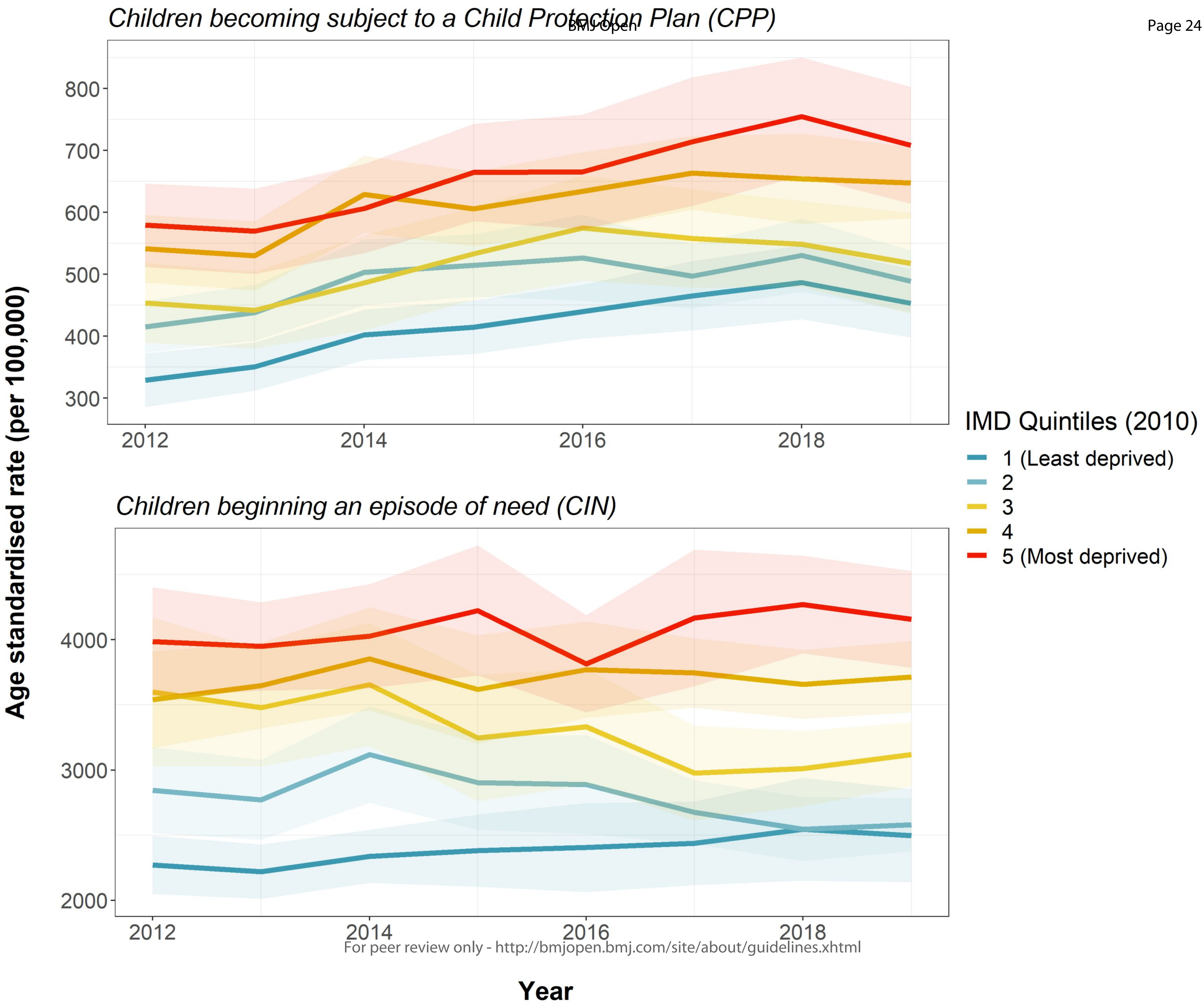
Children becoming subject to a Child Protection Plan (CPP)
Children beginning an episode of need (CIN)
Age standardised rate (per 100,000)
Year
800
700
600
500
400
300
4000
3000
2000
2012
2014
2016
2018
IMD Quintiles (2010)
1 (Least deprived)
2
3
4
5 (Most deprived)

SUPPLEMENTARY MATERIALS

Appendix 1: Harmonising data

Where changes to LA boundaries in 2009 led to the formation of two upper tier unitary authorities from a single county, CLA numbers for preceding years were split between these LAs based on their 2009 child population ratio. In the publicly available data, for reasons of confidentiality, numbers from one to five inclusive were supressed. For each missing value we therefore imputed a random integer in this range. There were only three cases of missing data, across two years, early in the implementation of the CIN census: age stratified data were not available for Havering and Newham in 2012, or Norfolk in 2013. Given the low degree of missingness, we performed complete case analyses.

Appendix 2: Model formulae

Segmented linear regression model for age standardised CLA rate, including linear spline:

$$y_{ij} = \beta_0 + \beta_1 x_{1ij} + \beta_2 x_{2i} + \beta_3 x_{3j} + \beta_4 x_{4j} + \beta_5 x_{3j} x_{2i} + \beta_5 x_{4j} x_{2i} + U_i + V_i x_{3j} + \varepsilon_{ij}$$

Let:

- y_{ij} denote the rate of children taken into care in LA i in year j
- x_{1ij} denote covariate lagged unemployment rate, coded as a continuous variable and dependent on LA i and on year j
- x_{2i} denote the weighted rank of deprivation dependent on LA i, a continuous variable ranging from 0 to 1
- x_{3j} denote the first spline term, which is year j coded as continuous variable and centered at 2004
- x_{4j} denote the second spline term, a continuous variable that takes the value of 0 for year j≤2007, and j−2007 for year j>2007. This defines a segmented regression with knot in 2007.
- $(U_{i,}\ V_i) \sim BVN(0, S_0)$ denote random intercept and slope for LA i
- $\varepsilon_{ij} \sim N\ (0, S_1)$ denote the random error for LA i in year j

Linear regression model for age standardised CPP and CIN rates:

$$y_{ij} = \beta_0 + \beta_1 x_{1ij} + \beta_2 x_{2i} + \beta_3 x_{3j} + \beta_4 x_{3j} x_{2i} + U_i + V_i x_{3j} + \varepsilon_{ij}$$

Let:

- y_{ij} denote the rate of children taken into care in LA i in year j
- x_{1ij} denote covariate lagged unemployment rate, coded as a continuous variable and dependent on LA i and on year j

For peer review only

- x_{2i} denote the weighted rank of deprivation dependent on LA i, a continuous variable ranging from 0 to 1
- x_{3j} denote year j, coded as continuous variable and centered at 2004
- $(U_{i,}\ V_i) \sim BVN(0, S_0)$ denote random intercept and slope for LA i
- $\varepsilon_{ij} \sim N\ (0, S_1)$ denote the random error for LA i in year j

Appendix 3: Breakpoint analysis

In our model for age standardised CLA rates, we used an iterative search procedure in order to identify which breakpoint offered the best fit. Appendix figure 17 shows the BIC value for each successive breakpoint used in the model. This led us to fit a knot in 2007.

Appendix 4: Age stratified analyses of crude CLA, CPP and CIN rates by LA deprivation quintile

For the age stratified analyses, we calculated rates for each of our outcomes using child population data, broken down by the same age bands available in the routine and FoI data, sourced from the Office for National Statistics (ONS) mid-year population estimates, accessed via Stat-Xplore[1]. These formed our denominator. We plotted rates for all age-stratified outcomes, across years for which data were available, enabling a comparison, by age group, across outcomes. Appendix figure 1 shows that the rise in CLA rates was mainly due to children under the age of 1 and children aged 16-17 entering care. Though wide, the gap in rates between most and least deprived LAs for the youngest age group does not appear to be widening. In the oldest age group however, there is a pronounced increase in the gap from 2010.

We sought to determine whether trends in CLA were reflected through the funnel of children's social care (appendix figures 2-3). The funnel remains widest in children under the age of 1. However, the gap between most and least deprived areas is relatively stable over time regardless of the stage. In children aged 16-17, the funnel narrows considerably from CIN to CPP, then widens once more at the level of CLA. The discontinuity is unique to this age group and may relate to the CPP's focus on risks within the family home. Acute risks to older children are often in the community, from peer groups and criminal networks. This may lead children to be placed directly on a CLA when need becomes acute. The gap in rates

[1] Office for National Statistics. Population Estimates for UK, England and Wales, Scotland and Northern Ireland: Stat-Xplore; 2019 [Available from: https://stat-xplore.dwp.gov.uk].

For peer review only

between most and least deprived areas appears to be widening in both CIN and CLA for this older age group: trends in CLA may well be reflecting, and concentrating, trends in CIN.

Appendix 5: Crude CPP rates by deprivation quintile, stratified by category of abuse

We plotted CPP rates for all categories of abuse, enabling a comparison across categories (appendix figure 4). Neglect, then emotional abuse, are the most commonly recorded primary categories of abuse. Rates for these categories are rising. Where neglect is recorded, the gap in rates between most and least deprived areas appears to have declined slightly from 2014. In contrast, where emotional abuse is recorded, the gap increased dramatically from 2014. Further research is needed to understand how recording practices, child welfare systems, social care practices, and underlying need, may differ by area level income deprivation.

Appendix 6: predictions based on the model

We predict expected CLA rates if the rise in rates from 2007 had occurred in more deprived LAs as it did in the median LA (such that 50% of the 2008 child population live in more deprived areas):

$$y_{ij} = \beta_0 + \beta_1 x_{1ij} + \beta_2 x_{2i} + \beta_3 x_{3j} + \beta_4 x_{4j} + \beta_5 x_{3j} x_{2i} + \beta_5 x_{4j} \boldsymbol{x_5} + U_i + V_i x_{3j} + \varepsilon_{ij}$$

Where $\boldsymbol{x_5}$ denotes the weighted rank of deprivation in the median LA in the cumulative distribution. This scenario preserves the change in trend from 2007 and unemployment rates, but posits that, after controlling for unemployment rates, the change in trend should not disproportionately affect areas based on their levels of income deprivation. Appendix figure 18, showing LAs grouped by quintiles, illustrates predicted rates according to this scenario.

Appendix 7: Full model output[2]

The following tables summarise the full output for each of the models in turn:

- Age standardised CLA rates
- Age standardised CLA rates, log-transformed (results exponentiated)
- Age standardised CPP rates
- Age standardised CIN rates

[2] Hlavac M. stargazer: Well-Formatted Regression and Summary Statistics Tables. R package version 5.2.2.; 2018.

For peer review only

Model	**CLA, 2004-2019**	**CLA, 2004-2019**	**CPP, 2012-2019**	**CIN, 2012-2019**
Dependent variable	CLA rate (per 100,000)	Log-transformed CLA rate (per 100,000)	CPP rate (per 100,000)	CIN rate (per 100,000)
Intercept	104.31** (71.56, 137.05)	114.41 ** (101.41, 129.07)	365.74** (306.30, 425.19)	2,190.79** (1,798.70, 2,582.88)
Unemployment rate (lagged)	8.95** (6.48, 11.43)	1.04** (1.03, 1.05)	-10.41 (-22.17, 1.36)	68.52 (-3.06, 140.10)
Spline 1	3.43 (-3.41, 10.27)	1.03** (1.01, 1.06)	12.69** (3.09, 22.29)	-6.76 (-74.71, 61.19)
Deprivation	192.93** (140.01, 245.86)	2.51** (2.07, 3.05)	304.12** (198.42, 409.81)	1,637.02** (949.98, 2,324.07)
Spline 2	1.89 (-5.21, 8.99)	1.00 (0.98, 1.03)	4.38 (-11.20, 19.95)	47.08 (-62.71, 156.88)
Spline 1: deprivation	-11.38* (-22.27, -0.49)	-0.94** (-0.90, -0.98)	-	-
Spline 2: deprivation	14.86* (3.55, 26.16)	1.06** (1.01, 1.10)	-	-
Observations	2,400	2,400	1,197	1,195
Log Likelihood	-13,279.74	211.43	-7,599.82	-9,727.10
Akaike Inf. Crit.	26,581.49	-400.87	15,217.63	19,472.20
Bayesian Inf. Crit.	26,645.10	-337.25	15,263.42	19,517.98
Note:	* p < 0.05, ** p < 0.01	* p < 0.05, ** p < 0.01	* p < 0.05, ** p < 0.01	* p < 0.05, ** p < 0.01
		All coefficients are exponentiated		

For peer review only

Appendix 8: residual diagnostics

The residuals from our model are normally distributed. Plotting standard normal quantiles against the data results in a relatively linear pattern. When grouped by quintile, predicted and observed values of CLA rates appear relatively consistent:

a. CLA model (absolute inequalities): see appendix figures 5-7
b. CLA model (relative inequalities): see appendix figures 8-10
c. CPP model: see appendix figures 11-13
d. CIN model: see appendix figures 14-16

For peer review only

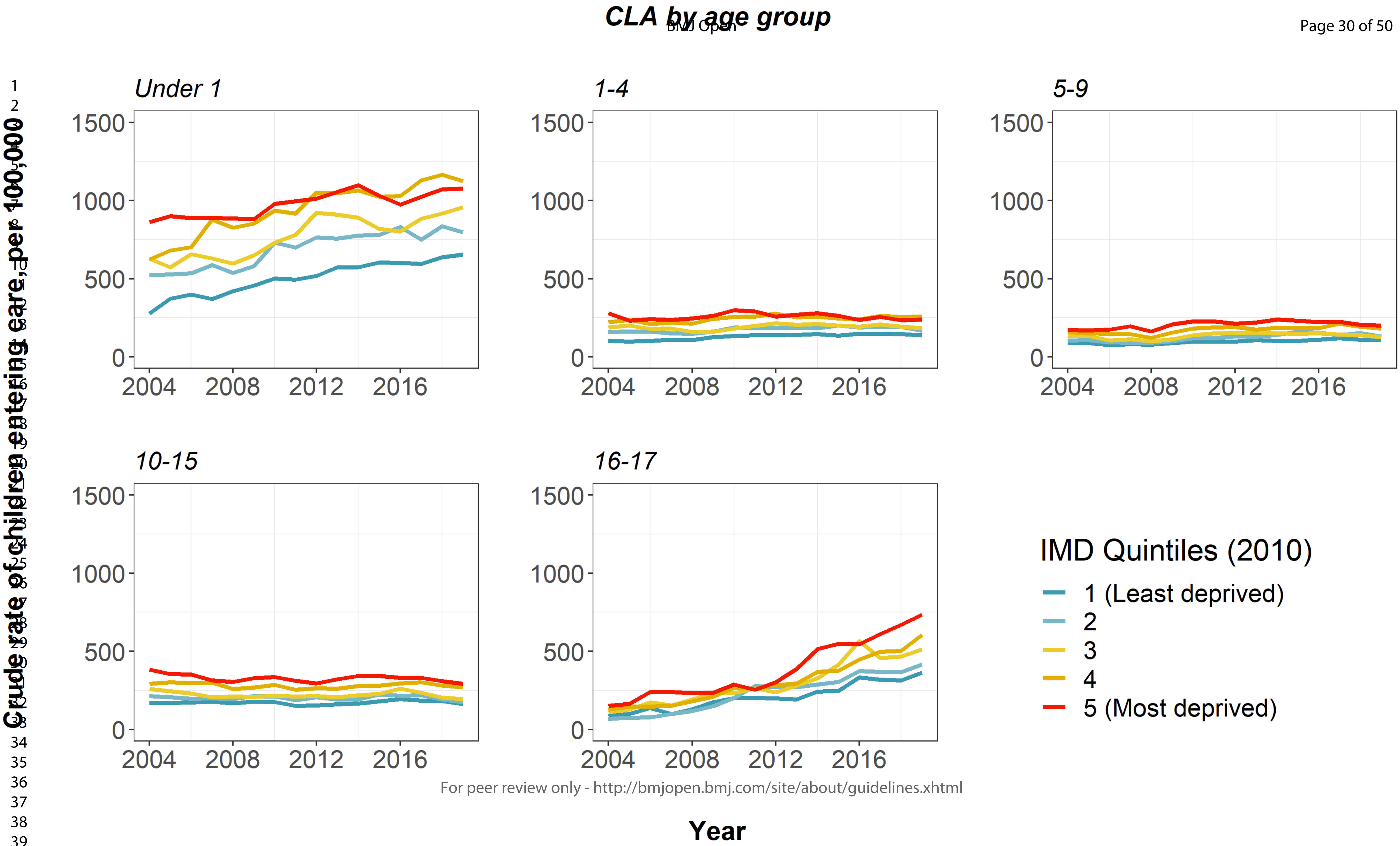
CLA by age group
Under 1
1-4
5-9
10-15
16-17
1500
1000
500
0
2004
2008
2012
2016
IMD Quintiles (2010)
1 (Least deprived)
2
3
4
5 (Most deprived)
Crude rate of children entering care, per 100,000
Year

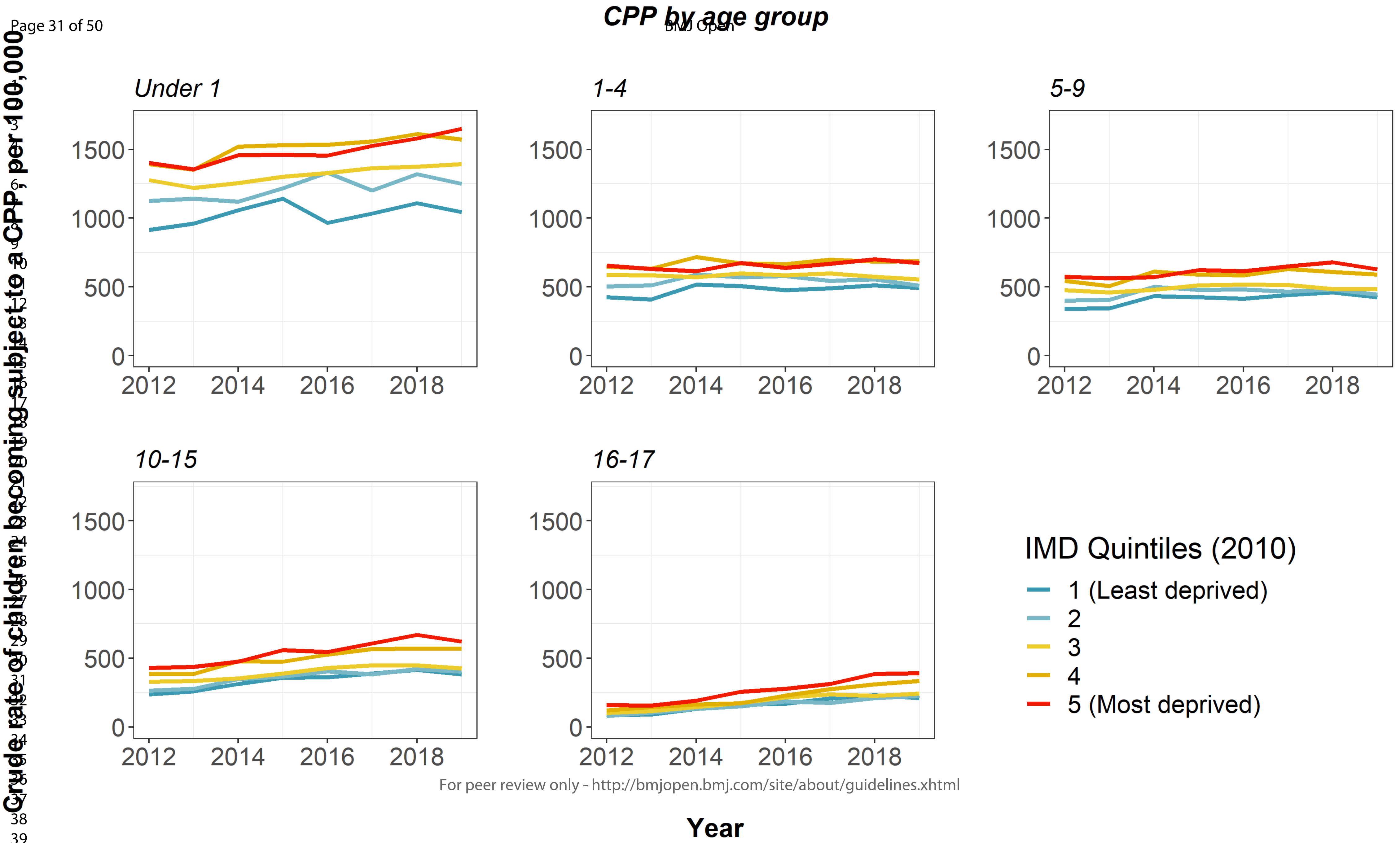
CPP by age group
Crude rate of children becoming subject to a CPP, per 100,000
Under 1
1-4
5-9
10-15
16-17
0
500
1000
1500
2012
2014
2016
2018
Year
IMD Quintiles (2010)
1 (Least deprived)
2
3
4
5 (Most deprived)

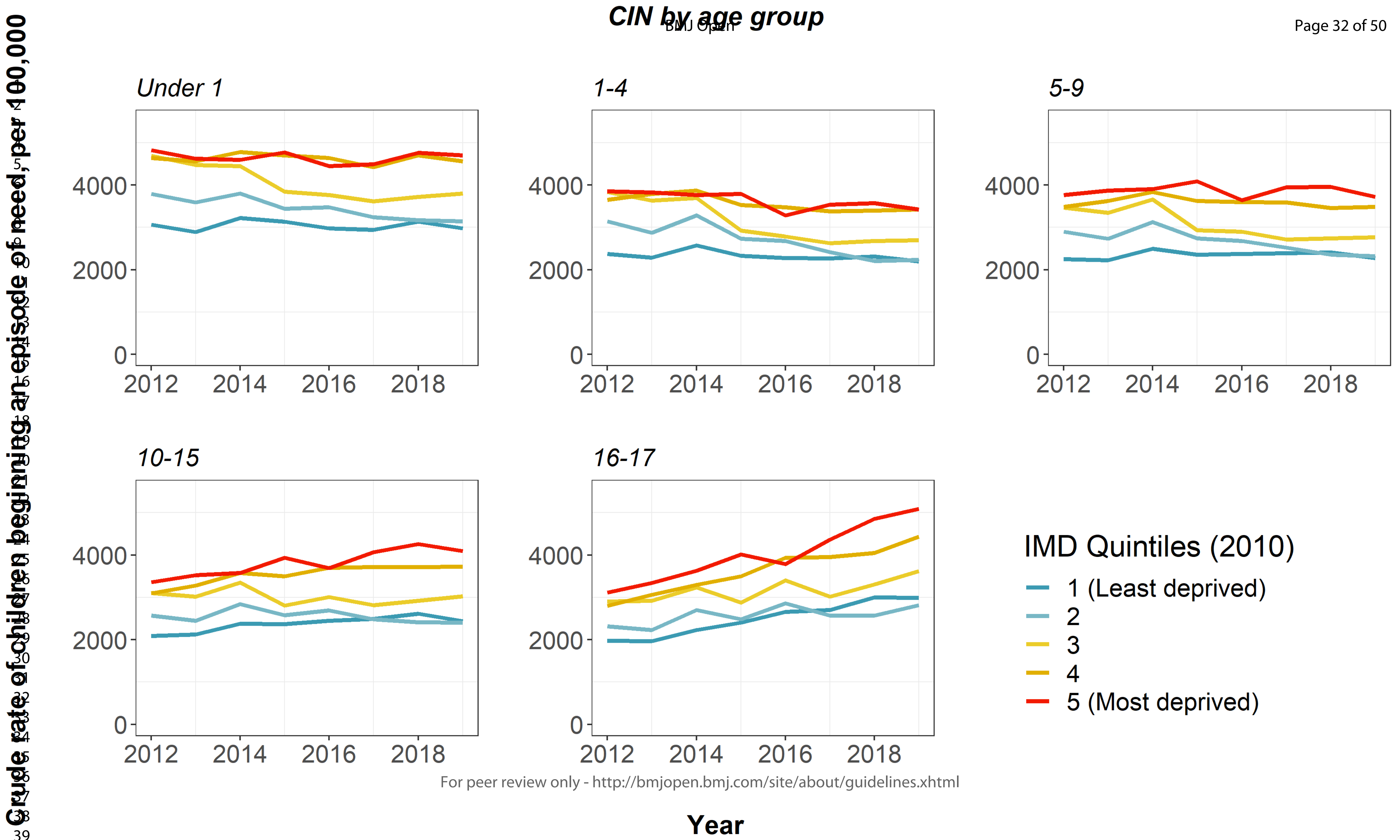
CIN by age group
Under 1
1-4
5-9
10-15
16-17
4000
2000
0
2012
2014
2016
2018
Crude rate of children beginning an episode of need, per 100,000
Year
IMD Quintiles (2010)
1 (Least deprived)
2
3
4
5 (Most deprived)

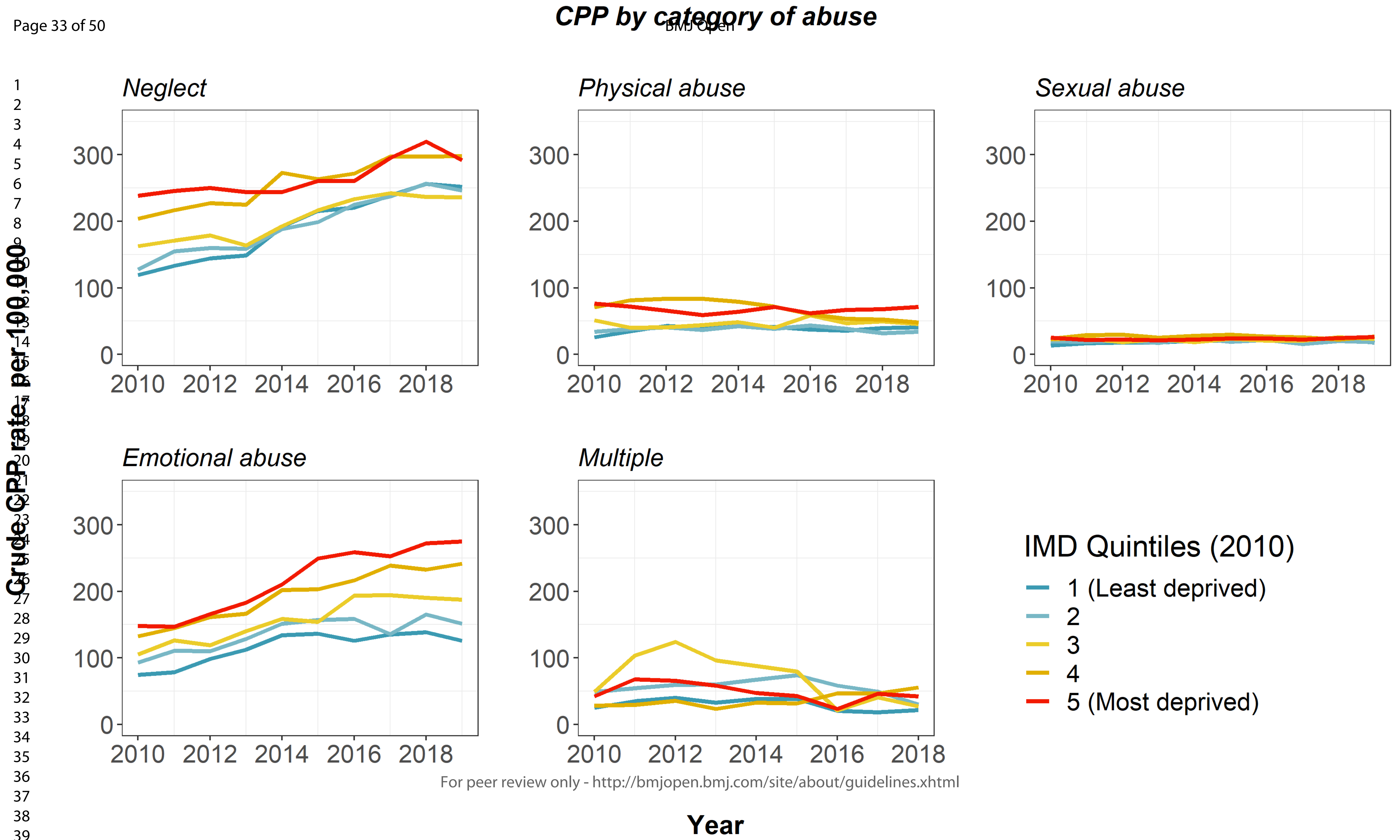
CPP by category of abuse
Neglect
Physical abuse
Sexual abuse
Emotional abuse
Multiple
Crude CPP rate, per 100,000
Year
0
100
200
300
2010
2012
2014
2016
2018
IMD Quintiles (2010)
1 (Least deprived)
2
3
4
5 (Most deprived)

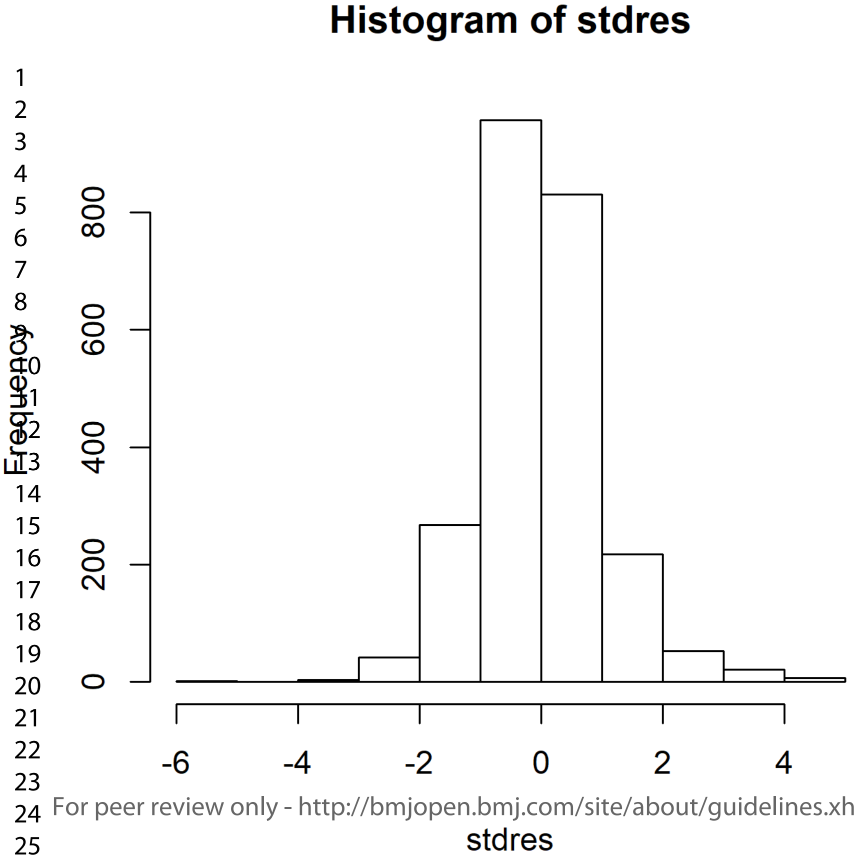

For peer review only - http://bmjopen.bmj.com/site/about/guidelines.xhtml

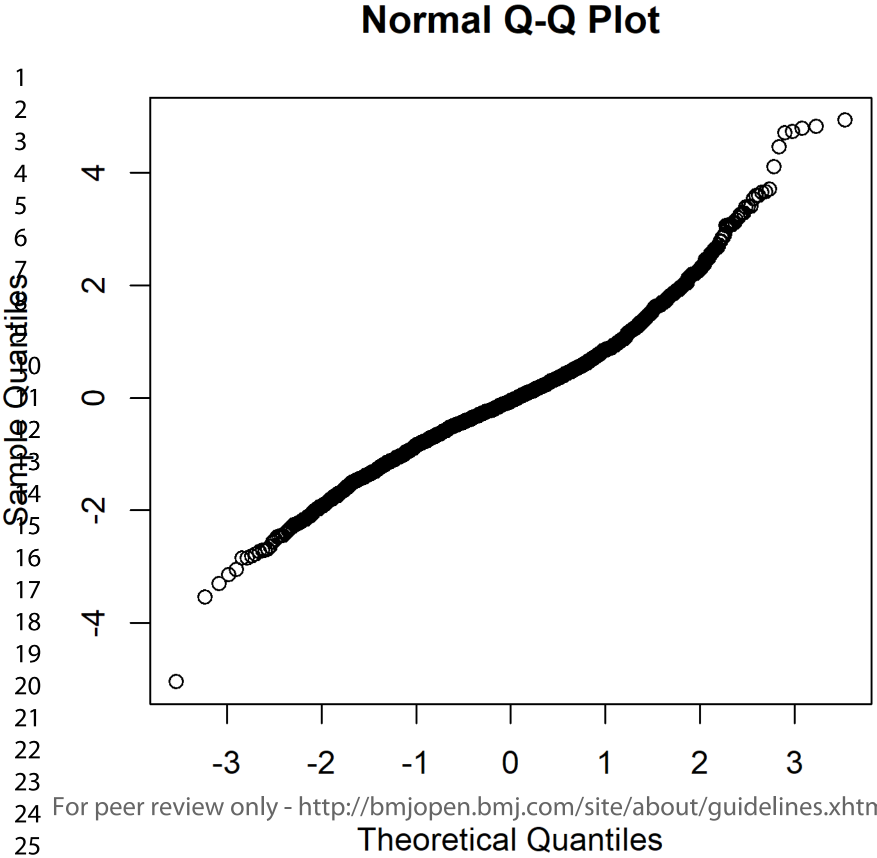

For peer review only - http://bmjopen.bmj.com/site/about/guidelines.xhtml

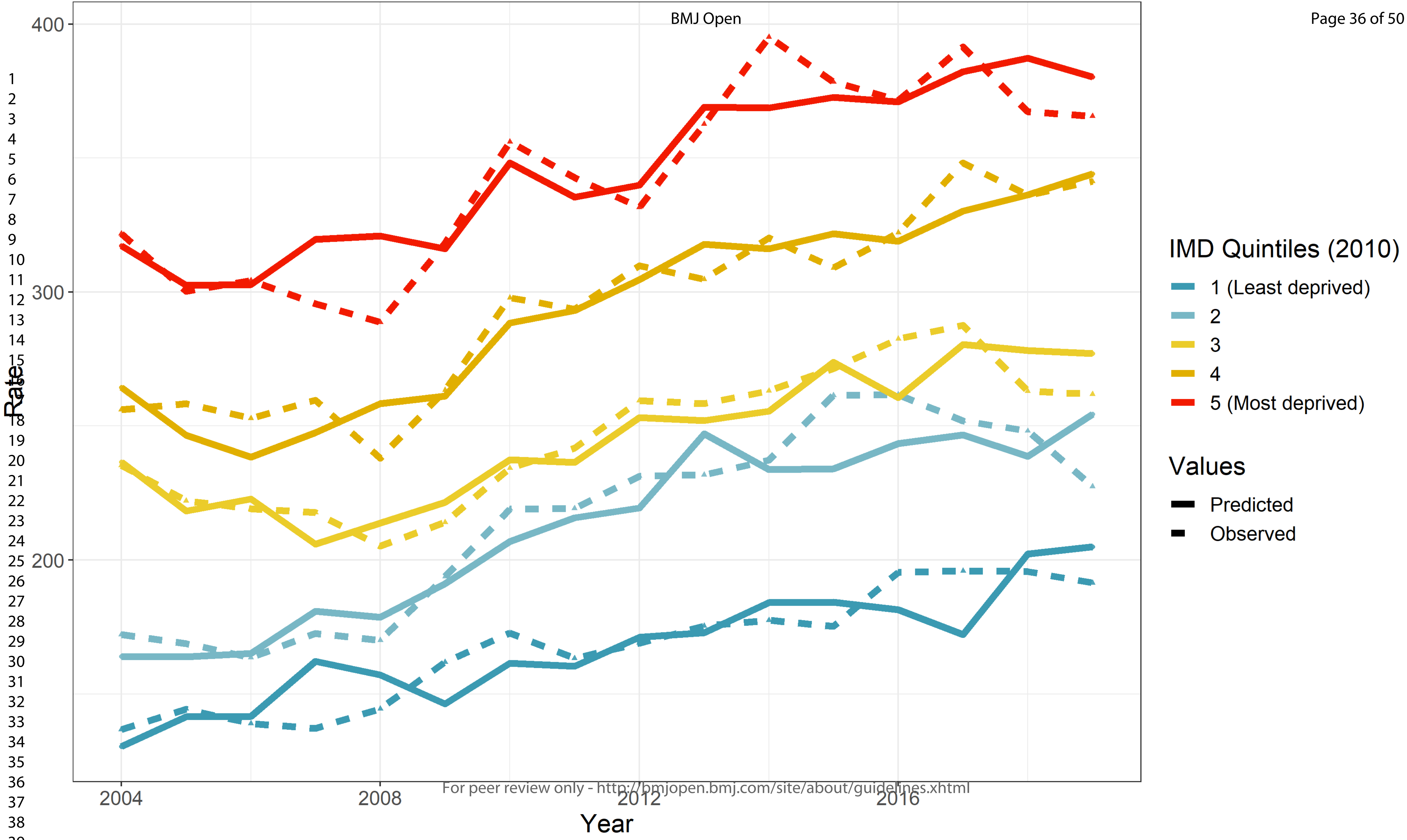
400
300
200
Rate
2004
2008
2012
2016
Year
IMD Quintiles (2010)
1 (Least deprived)
2
3
4
5 (Most deprived)
Values
Predicted
Observed

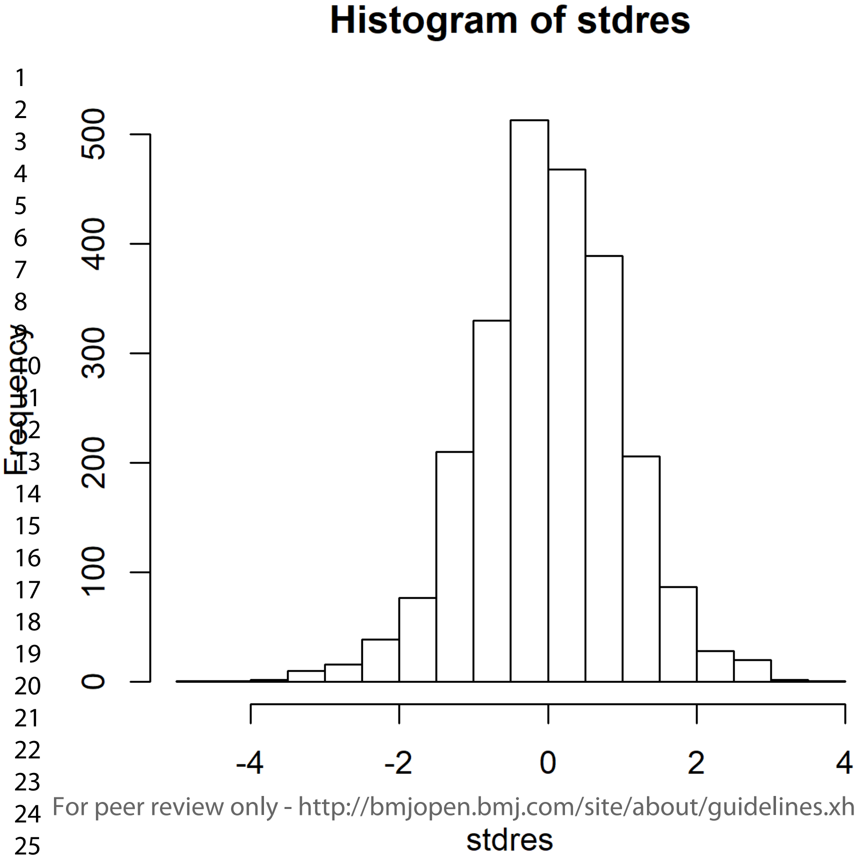
Histogram of stdres
Frequency
0
100
200
300
400
500
-4
-2
0
2
4
stdres

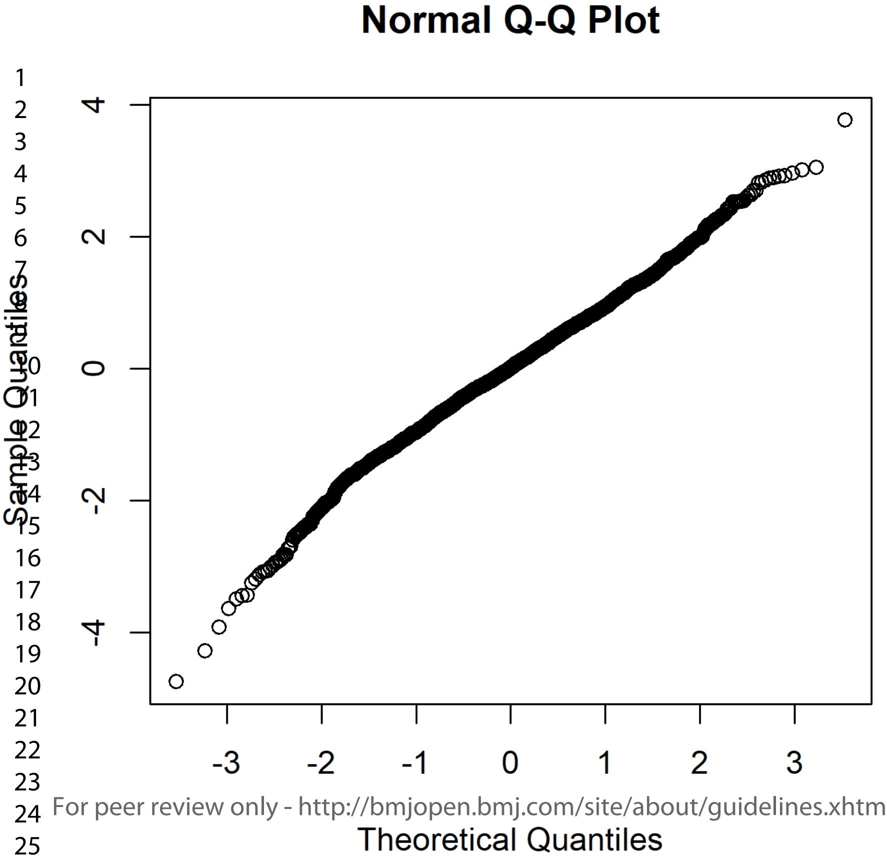

For peer review only - http://bmjopen.bmj.com/site/about/guidelines.xhtml

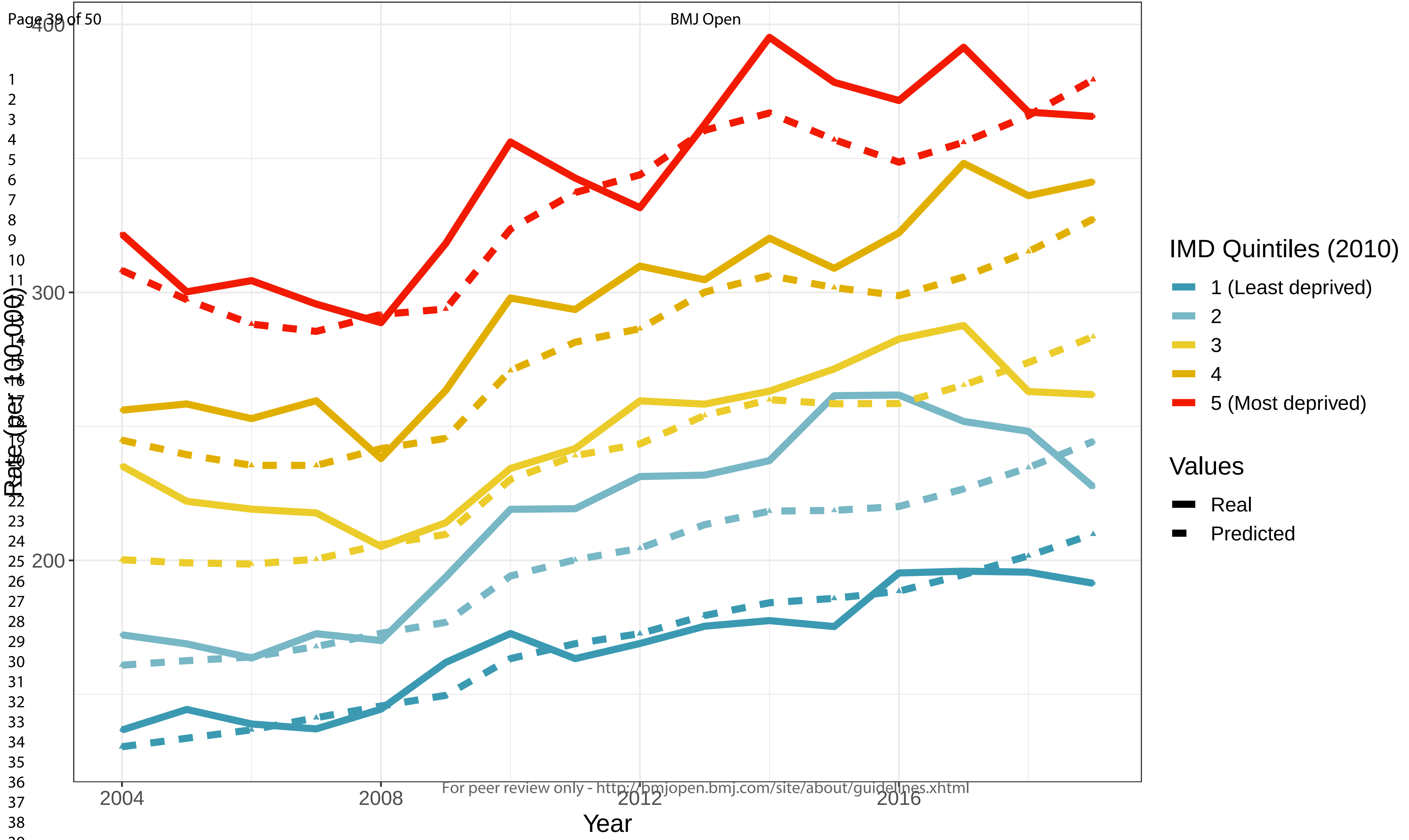

For peer review only - http://bmjopen.bmj.com/site/about/guidelines.xhtml

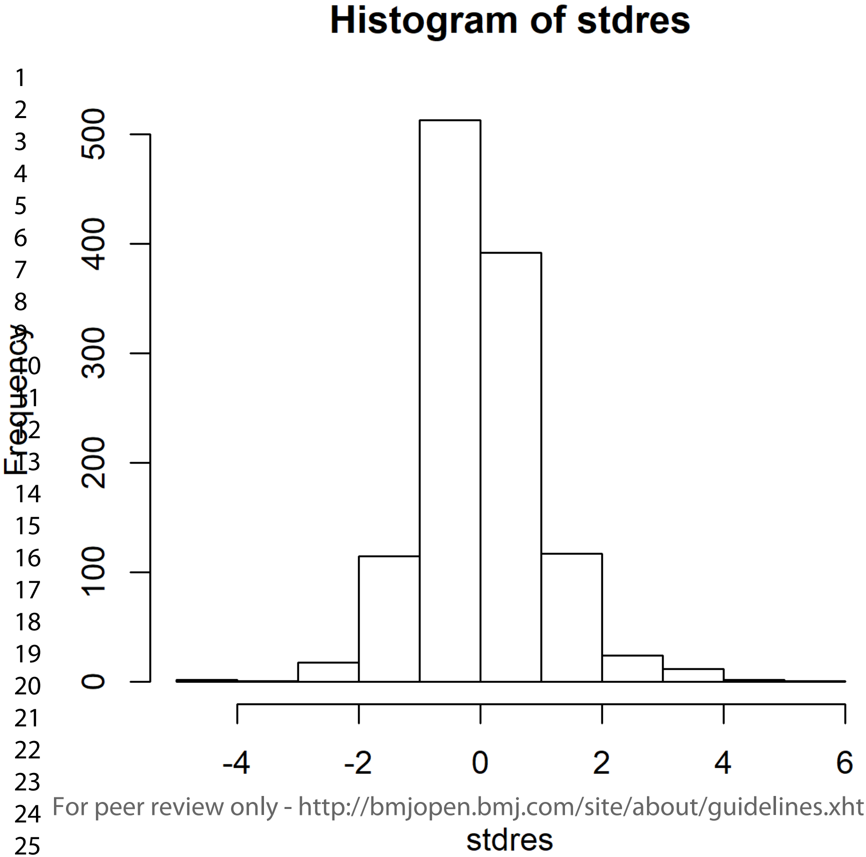
Histogram of stdres
Frequency
0
100
200
300
400
500
-4
-2
0
2
4
6
stdres

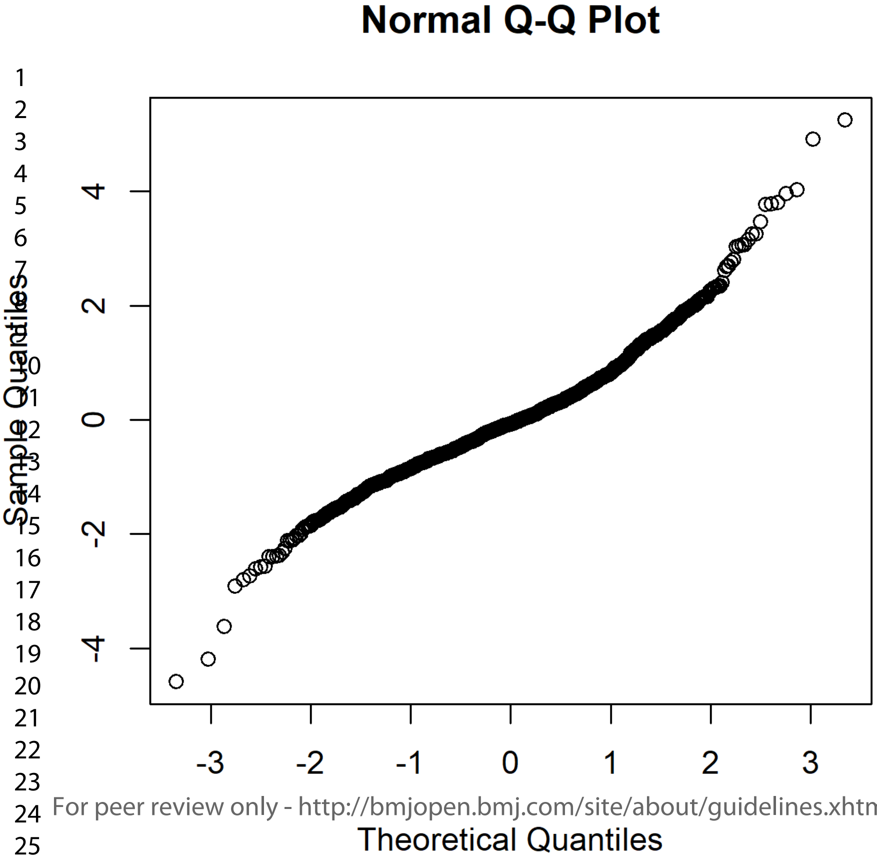

For peer review only - http://bmjopen.bmj.com/site/about/guidelines.xhtml

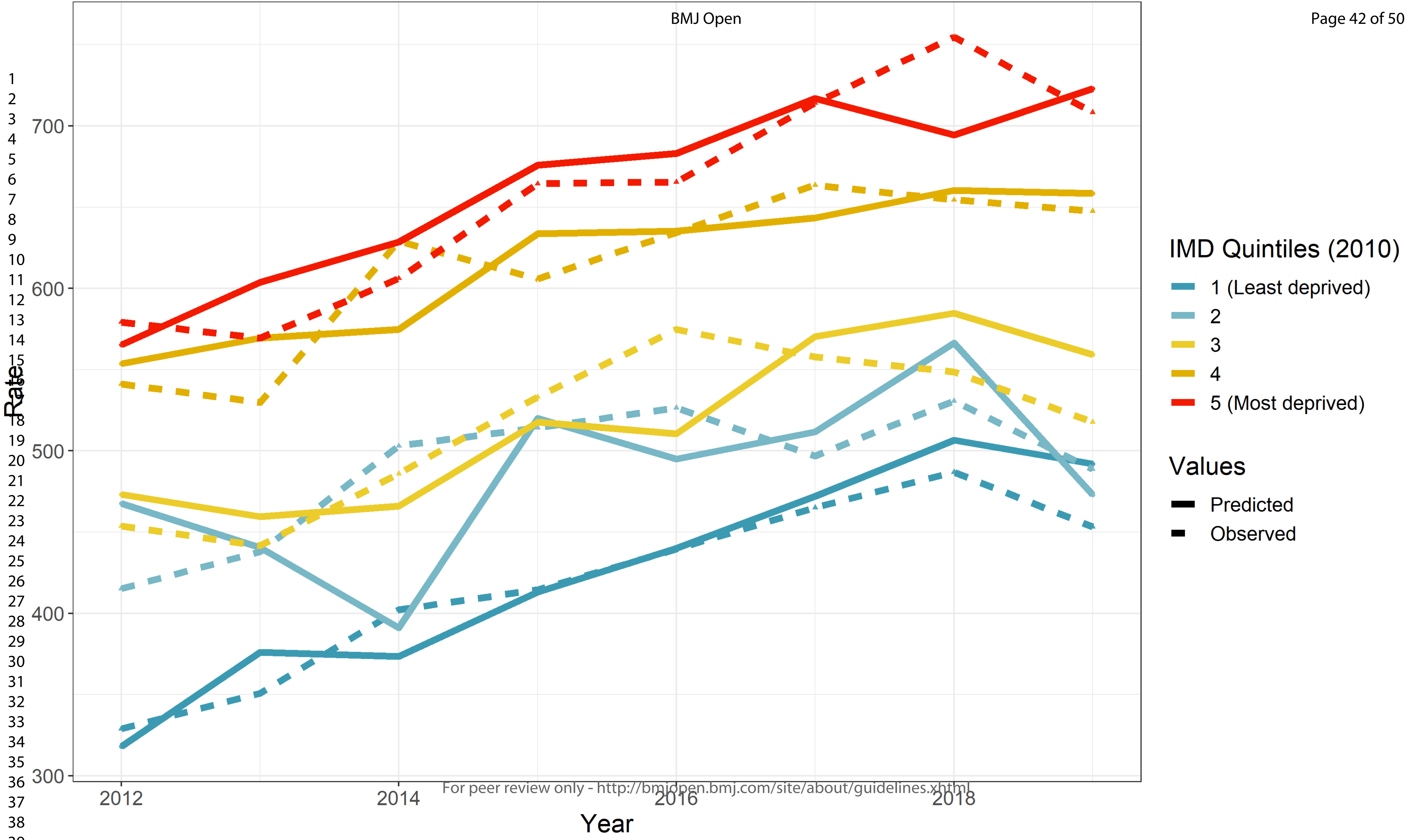
Rate
700
600
500
400
300
2012
2014
2016
2018
Year
IMD Quintiles (2010)
1 (Least deprived)
2
3
4
5 (Most deprived)
Values
Predicted
Observed

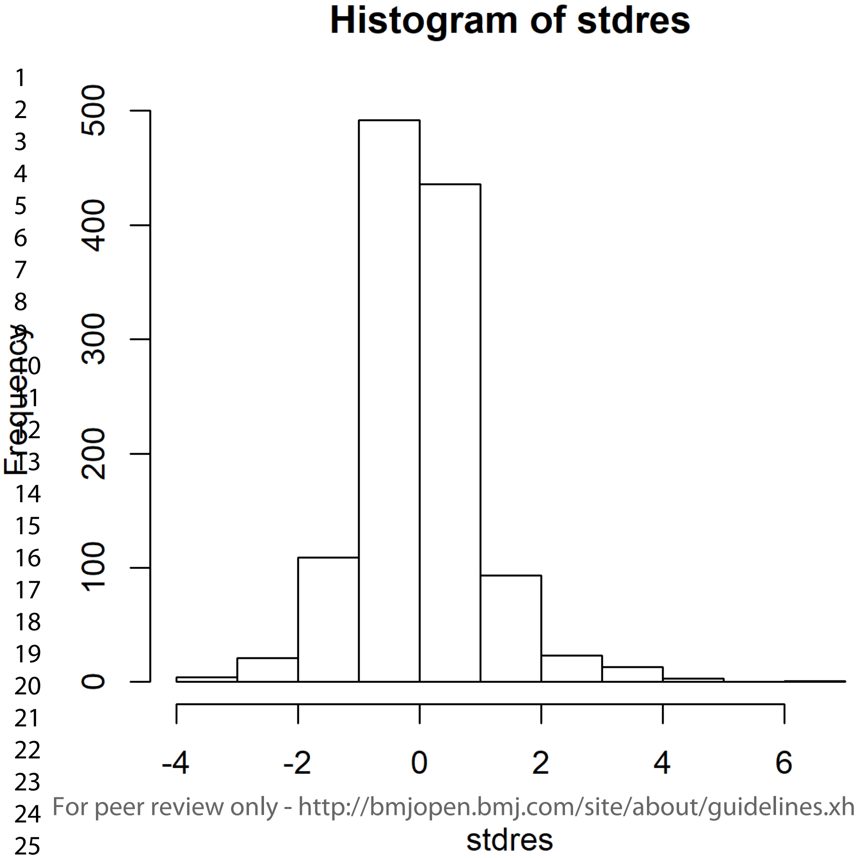
Histogram of stdres
Frequency
0
100
200
300
400
500
-4
-2
0
2
4
6
stdres

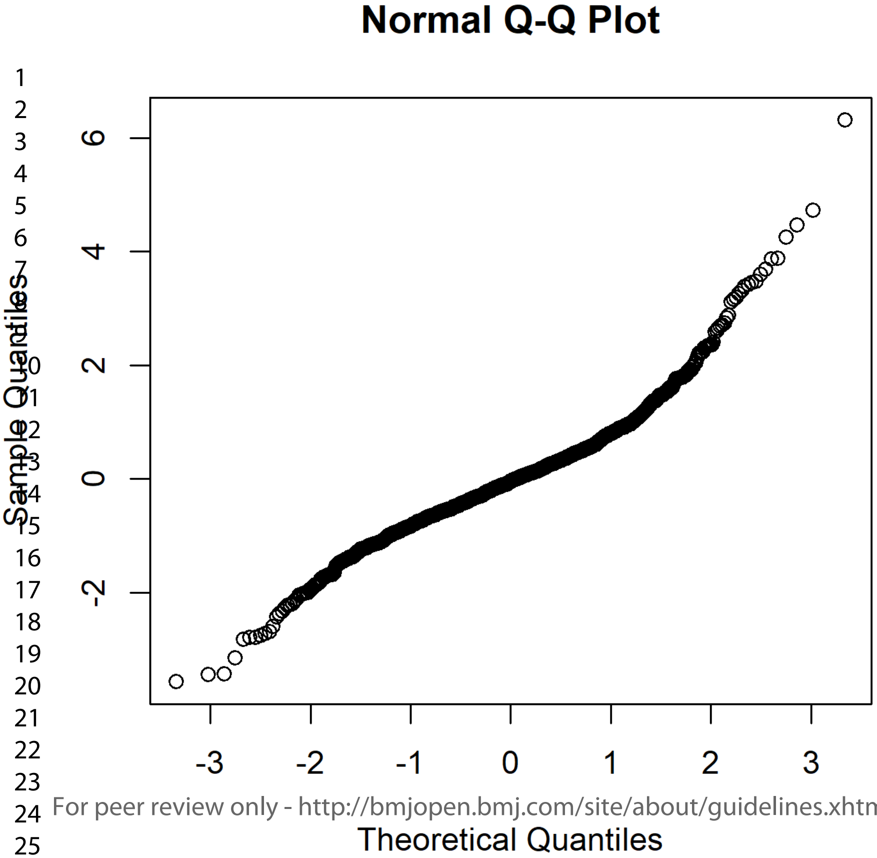

For peer review only - http://bmjopen.bmj.com/site/about/guidelines.xhtml

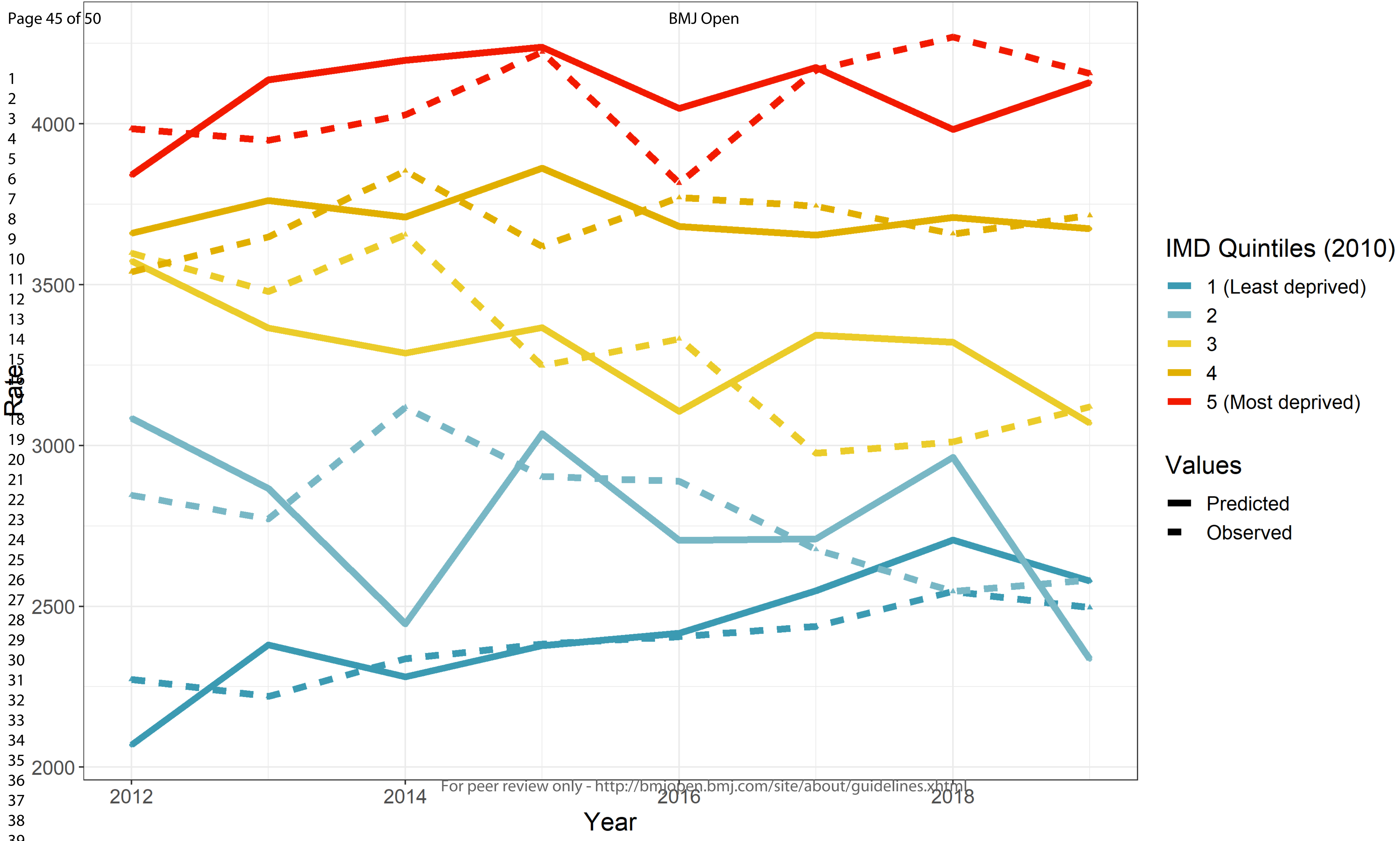
Rate
4000
3500
3000
2500
2000
2012
2014
2016
2018
Year
IMD Quintiles (2010)
1 (Least deprived)
2
3
4
5 (Most deprived)
Values
Predicted
Observed

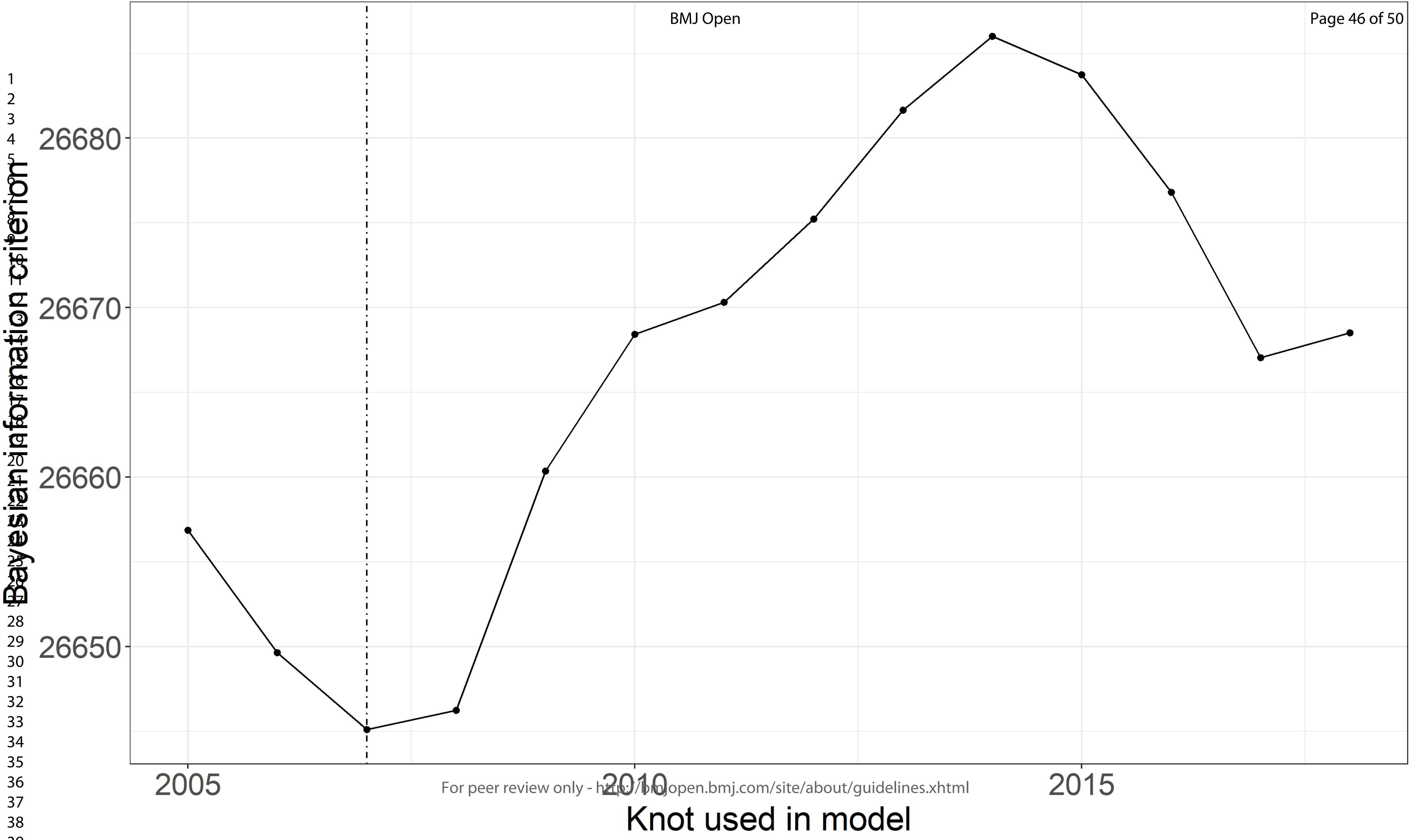

For peer review only - http://bmjopen.bmj.com/site/about/guidelines.xhtml

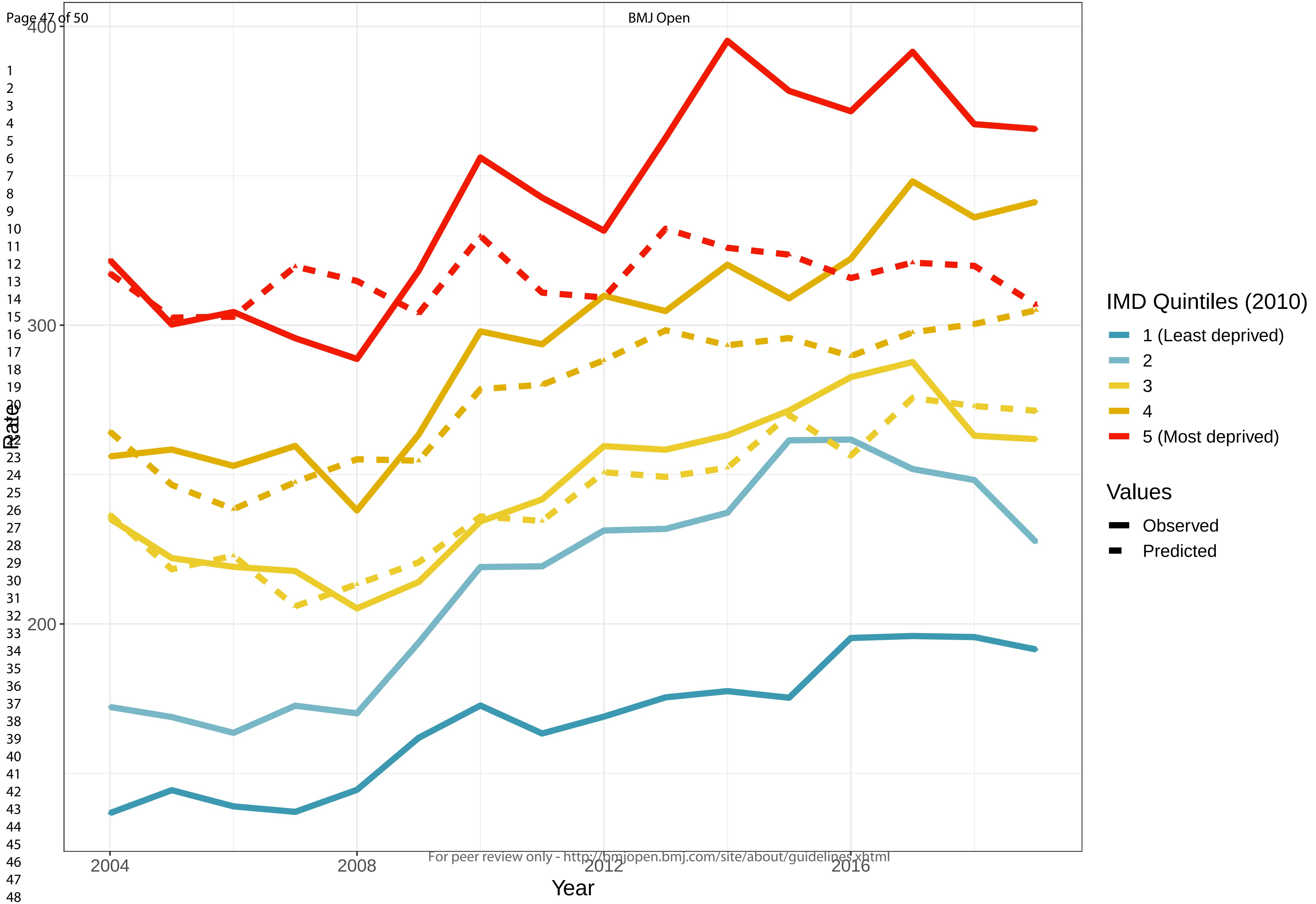
400
300
200
Rate
2004
2008
2012
2016
Year
IMD Quintiles (2010)
1 (Least deprived)
2
3
4
5 (Most deprived)
Values
Observed
Predicted

STROBE Statement—checklist of items that should be included in reports of observational studies

	Item No	Recommendation
**Title and abstract**	1	(*a*) Indicate the study's design with a commonly used term in the title or the abstract<br>Done. TITLE: "Trends in inequalities in looked after children in England 2004-2019: a local area ecological analysis"
		(*b*) Provide in the abstract an informative and balanced summary of what was done and what was found<br>Done. ABSTRACT: page 2
**Introduction**		
Background/rationale	2	Explain the scientific background and rationale for the investigation being reported.<br>Done. BACKGROUND: page 3
Objectives	3	State specific objectives, including any prespecified hypotheses<br>Done. BACKGROUND: page 2. "We investigated whether the rate of children becoming looked after between 2004 and 2019 increased more in deprived areas of the country – areas that, relative to more affluent areas, have higher levels of poverty – and the contribution of unemployment to these trends. We further quantify trends in inequalities in children experiencing other forms of child welfare intervention, in order to assess whether findings for CLA are consistent across child welfare outcomes."
**Methods**		
Study design	4	Present key elements of study design early in the paper<br>Done. METHODS: page 4
Setting	5	Describe the setting, locations, and relevant dates, including periods of recruitment, exposure, follow-up, and data collection.<br>Done. METHODS / DATA SOURCES AND MEASURES: pages 4-5
Participants	6	(*a*) *Cohort study*—Give the eligibility criteria, and the sources and methods of selection of participants. Describe methods of follow-up<br>NA: participants are the entire child population<br>*Case-control study*—Give the eligibility criteria, and the sources and methods of case ascertainment and control selection. Give the rationale for the choice of cases and controls<br>NA<br>*Cross-sectional study*—Give the eligibility criteria, and the sources and methods of selection of participants<br>NA
		(*b*) *Cohort study*—For matched studies, give matching criteria and number of exposed and unexposed<br>NA<br>*Case-control study*—For matched studies, give matching criteria and the number of controls per case<br>NA
Variables	7	Clearly define all outcomes, exposures, predictors, potential confounders, and effect modifiers. Give diagnostic criteria, if applicable.<br>Done. METHODS / DATA SOURCES AND MEASURES: pages 4-5

For peer review only - http://bmjopen.bmj.com/site/about/guidelines.xhtml

Data sources/ measurement	8*	For each variable of interest, give sources of data and details of methods of assessment (measurement). Describe comparability of assessment methods if there is more than one group.<br>Done. METHODS / DATA SOURCES AND MEASURES: pages 4-5
Bias	9	Describe any efforts to address potential sources of bias<br>Done. METHODS / DATA SOURCES AND MEASURES: pages 4-5. Use of routinely available data 2004-19 to assess descriptive trends.
Study size	10	Explain how the study size was arrived at.<br>NA: the study size is the entire child population of England, analysed as aggregate data for 150 local authorities
Quantitative variables	11	Explain how quantitative variables were handled in the analyses. If applicable, describe which groupings were chosen and why.<br>Done. METHODS/ / DATA SOURCES AND MEASURES: pages 4-5. "For our categorical measure of deprivation, LAs were assigned to quintiles according to their income deprivation score such that 20% of the 2008 child population was apportioned to each quintile."; "We also used a continuous measure of the income deprivation score, converted to a weighted rank by assigning a value from 0 to 1 based on the midpoint of the LA's range in the cumulative distribution. When using this value as a continuous exposure variable in the regression model, the estimated coefficient expresses the Slope Index of Inequality (SII)"
Statistical methods	12	(a) Describe all statistical methods, including those used to control for confounding.<br>Done. METHODS / STATISTICAL ANALYSIS: page 7
		(*b*) Describe any methods used to examine subgroups and interactions<br>Done. METHODS / STATISTICAL ANALYSIS: page 7 . "We included an interaction between the spline terms for the effect of year and deprivation to allow for potential differences in trend by SEC."
		(*c*) Explain how missing data were addressed<br>Done. SUPPLEMENTARY MATERIALS / APPENDIX 3. "In the data, for reasons of confidentiality, numbers from one to five inclusive were supressed. For each missing value we therefore imputed a random integer in this range. There were only three cases of missing data, across two years, early in the implementation of the CIN census: age stratified data were not available for Havering and Newham in 2012, or Norfolk in 2013. Given the low degree of missingness, we performed complete case analyses."
		(*d*) *Cohort study*—If applicable, explain how loss to follow-up was addressed<br>NA<br>*Case-control study*—If applicable, explain how matching of cases and controls was addressed<br>NA<br>*Cross-sectional study*—If applicable, describe analytical methods taking account of sampling strategy<br>NA
		(e) Describe any sensitivity analyses.<br>NA
**Results**		
Participants	13*	(a) Report numbers of individuals at each stage of study—eg numbers potentially eligible, examined for eligibility, confirmed eligible, included in the study, completing follow-up, and

For peer review only - http://bmjopen.bmj.com/site/about/guidelines.xhtml

		analysed<br>NA
		(b) Give reasons for non-participation at each stage<br>NA
		(c) Consider use of a flow diagram<br>NA
Descriptive data	14*	(a) Give characteristics of study participants (eg demographic, clinical, social) and information on exposures and potential confounders<br>NA
		(b) Indicate number of participants with missing data for each variable of interest<br>Done. SUPPLEMENTARY MATERIALS / APPENDIX 3. "In the data, for reasons of confidentiality, numbers from one to five inclusive were supressed. For each missing value we therefore imputed a random integer in this range. There were only three cases of missing data, across two years, early in the implementation of the CIN census: age stratified data were not available for Havering and Newham in 2012, or Norfolk in 2013. Given the low degree of missingness, we performed complete case analyses."
		(c) *Cohort study*—Summarise follow-up time (eg, average and total amount)<br>NA
Outcome data	15*	*Cohort study*—Report numbers of outcome events or summary measures over time<br>NA
		Case-control study—Report numbers in each exposure category, or summary measures of exposure<br>NA
		Cross-sectional study—Report numbers of outcome events or summary measures<br>NA
Main results	16	(*a*) Give unadjusted estimates and, if applicable, confounder-adjusted estimates and their precision (eg, 95% confidence interval). Make clear which confounders were adjusted for and why they were included.<br>Done. RESULTS: pages 5-8. We have provided data on unadjusted trends in descriptive plots. In the models we have adjusted for unemployment.
		(*b*) Report category boundaries when continuous variables were categorized<br>NA
		(*c*) If relevant, consider translating estimates of relative risk into absolute risk for a meaningful time period<br>Done. RESULTS: pages 5-8. Absolute change in rates reported.
Other analyses	17	Report other analyses done—eg analyses of subgroups and interactions, and sensitivity analyses.<br>Done. SUPPLEMENTARY MATERIALS / APPENDICES 6-7. Age stratified analyses of crude CLA, CPP and CIN rates by LA deprivation quintile; Crude CPP rates by deprivation quintile, stratified by category of abuse
**Discussion**		
Key results	18	Summarise key results with reference to study objectives.<br>Done. DISCUSSION: page 9
Limitations	19	Discuss limitations of the study, taking into account sources of potential bias or imprecision. Discuss both direction and magnitude of any potential bias<br>Done. DISCUSSION: page 9
Interpretation	20	Give a cautious overall interpretation of results considering objectives, limitations,

For peer review only - http://bmjopen.bmj.com/site/about/guidelines.xhtml

		multiplicity of analyses, results from similar studies, and other relevant evidence. Done. DISCUSSION: pages 9-11
Generalisability	21	Discuss the generalisability (external validity) of the study results. Done. DISCUSSION / STRENGTHS AND LIMITATIONS: page 10
**Other information**		
Funding	22	Give the source of funding and the role of the funders for the present study and, if applicable, for the original study on which the present article is based Done. FUNDING SOURCES: page 12.

*Give information separately for cases and controls in case-control studies and, if applicable, for exposed and unexposed groups in cohort and cross-sectional studies.

Note: An Explanation and Elaboration article discusses each checklist item and gives methodological background and published examples of transparent reporting. The STROBE checklist is best used in conjunction with this article (freely available on the Web sites of PLoS Medicine at http://www.plosmedicine.org/, Annals of Internal Medicine at http://www.annals.org/, and Epidemiology at http://www.epidem.com/). Information on the STROBE Initiative is available at www.strobe-statement.org.

For peer review only

For peer review only - http://bmjopen.bmj.com/site/about/guidelines.xhtml

BMJ Open

Trends in inequalities in looked after children in England 2004-2019: a local area ecological analysis

Journal:	*BMJ Open*
Manuscript ID	bmjopen-2020-041774.R2
Article Type:	Original research
Date Submitted by the Author:	30-Sep-2020
Complete List of Authors:	Bennett, Davara; University of Liverpool Faculty of Health and Life Sciences, Department of Public Health and Policy<br>Mason, Kate; University of Liverpool Faculty of Health and Life Sciences, Department of Public Health and Policy<br>Schlüter, Daniela; University of Liverpool Faculty of Health and Life Sciences, Department of Public Health and Policy<br>Wickham, S; University of Liverpool Faculty of Health and Life Sciences, Department of Public Health and Policy<br>Lai, Eric; University of Liverpool Faculty of Health and Life Sciences, Department of Public Health and Policy<br>Alexiou, Alexandros; University of Liverpool Faculty of Health and Life Sciences, Department of Public Health and Policy<br>Barr, Ben ; University of Liverpool Faculty of Health and Life Sciences, Department of Public Health and Policy<br>Taylor-Robinson, David; University of Liverpool Faculty of Health and Life Sciences, Department of Public Health and Policy
**Primary Subject Heading**:	Public health
Secondary Subject Heading:	Epidemiology, Health policy
Keywords:	Child protection < PAEDIATRICS, PUBLIC HEALTH, Community child health < PAEDIATRICS

SCHOLARONE™
Manuscripts

I, the Submitting Author has the right to grant and does grant on behalf of all authors of the Work (as defined in the below author licence), an exclusive licence and/or a non-exclusive licence for contributions from authors who are: i) UK Crown employees; ii) where BMJ has agreed a CC-BY licence shall apply, and/or iii) in accordance with the terms applicable for US Federal Government officers or employees acting as part of their official duties; on a worldwide, perpetual, irrevocable, royalty-free basis to BMJ Publishing Group Ltd ("BMJ") its licensees and where the relevant Journal is co-owned by BMJ to the co-owners of the Journal, to publish the Work in this journal and any other BMJ products and to exploit all rights, as set out in our licence.

The Submitting Author accepts and understands that any supply made under these terms is made by BMJ to the Submitting Author unless you are acting as an employee on behalf of your employer or a postgraduate student of an affiliated institution which is paying any applicable article publishing charge ("APC") for Open Access articles. Where the Submitting Author wishes to make the Work available on an Open Access basis (and intends to pay the relevant APC), the terms of reuse of such Open Access shall be governed by a Creative Commons licence – details of these licences and which Creative Commons licence will apply to this Work are set out in our licence referred to above.

Other than as permitted in any relevant BMJ Author's Self Archiving Policies, I confirm this Work has not been accepted for publication elsewhere, is not being considered for publication elsewhere and does not duplicate material already published. I confirm all authors consent to publication of this Work and authorise the granting of this licence.

For peer review only

TITLE PAGE

Trends in inequalities in looked after children in England 2004-2019: a local area ecological analysis

Davara L Bennett[1] MSc, Kate E Mason[1] PhD, Daniela K Schlüter[1] PhD, Sophie Wickham[1] PhD, Eric TC Lai[1] PhD, Alexandros Alexiou[1] PhD, Ben Barr[1*] PhD, David Taylor-Robinson[1*] PhD

1. University of Liverpool, Faculty of Health and Life Sciences, Department of Public Health and Policy.

- Joint Senior Author

Correspondence to: Davara L Bennett, University of Liverpool, Waterhouse Building 2nd Floor Block F, L69 3GL, Liverpool, United Kingdom

E-mail: davara.bennett@liverpool.ac.uk

Word Count: 3,661

For peer review only

ABSTRACT

Objective: To assess trends in inequalities in children becoming looked after (CLA) in England between 2004 and 2019, after controlling for unemployment, a marker of the recession and risk factor for child maltreatment.

Design: longitudinal local area ecological analysis

Setting: 150 English upper-tier local authorities.

Participants: Children under the age of 18.

Primary outcome measure: The annual age-standardised rate of children becoming looked after (CLA rate) across English local authorities, grouped into quintiles based on their level of income deprivation. Slope indices of inequality (SII) were estimated using longitudinal segmented mixed effects models, controlling for unemployment.

Results: Since 2008, there has been a precipitous rise in CLA rates, and a marked widening of inequalities. Unemployment was associated with rising CLA rates: for each percentage point increase in the unemployment rate, an estimated additional 9 children per 100,000 (95% CI 6-11) per year became looked after the following year. However, inequalities increased independently of the effect of unemployment. Between 2007 and 2019, after controlling for unemployment, the gap between most and least deprived areas increased by 15 children per 100,000 per year (95% CI 4-26) relative to the 2004-2006 trend.

Conclusions: The dramatic increase in the number of CLA has been greater in poorer areas, and in areas more deeply affected by the recession. But trends in unemployment do not explain the decade-long rise in inequalities, suggesting that other socioeconomic factors, including rising child poverty and reduced spending on Children's Services, may be fuelling inequalities. Policies to safely reduce the rate of children becoming looked after should urgently address the social determinants of child health and wellbeing.

For peer review only

STRENGTHS AND LIMITATIONS OF THE STUDY

- This study is the first to quantify inequalities in child welfare outcomes in England longitudinally, using segmented mixed effects models to show that the gap in rates of children entering care between most and least deprived areas is on the rise after controlling for unemployment.
- The study uses routinely available data for the whole of England, and explores several child welfare outcomes in order to describe trends throughout the child welfare system.
- An important limitation is that, using an ecological area-level analysis, we cannot conclude that children entering care were directly affected by the exposures of interest.

For peer review only

INTRODUCTION

Improving the health outcomes and life chances of children looked after (CLA) is a matter of public health concern (1). In England, over the last decade, the prevalence of CLA increased dramatically, from 53 to 64 per 10,000 children, a rise of 20 percent. At last count, in March 2019, their number exceeded 78,000 (2). The health outcomes and life chances of these children, many of whom have experienced abuse, neglect and other forms of acute adversity, may differ markedly from those of their peers. On average, individuals who have been looked after face worse outcomes across a range of measures, throughout the life-course – physical and mental health, education, offending, employment, income – relative to those who have not come in contact with child welfare services. (3)

Reducing the economic burden associated with the consequences of CLA is of particular concern to policymakers: supporting CLA represents a major expenditure at local authority (LA) level. Across England, between 2011 and 2018, CLA spend increased by £1.9bn in real terms, to £4.6bn. Children's services have been described as approaching breaking point (4). Internationally, there have been increasing calls for a preventative approach to CLA that addresses upstream risk factors for child abuse and neglect (5).

A number of factors may have contributed to rising CLA rates in England over the last decade. High profile serious case reviews (6), shifting understanding of the impact of different forms of childhood adversity (7), and legal judgements clarifying LA statutory responsibilities (8), may all affect thresholds for child welfare intervention. Wider economic changes may also underlie trends in CLA rates. Growing up in adverse socioeconomic circumstances (SECs) is an important risk factor for child abuse and neglect and for children being taken into care (9), with poverty, unemployment and parental financial stress recognised as contributory causal factors (10,11). Several experimental and quasi-experimental studies from the US have shown that raising family income and reducing poverty leads to a reduction in rates of child abuse and neglect (10,12).

In 2008, the onset of the financial recession led to rising unemployment in England, and to fiscal policy with far-reaching social consequences. In 2010 the UK government began introducing a series of austerity measures with the stated intention of eliminating the budget deficit and reducing the national debt(13). The welfare system has been a principal focus of cuts and reforms (14). These have adversely affected, in particular, families with children and those at greatest risk of poverty, fuelling a rise in child poverty (15). At the same time,

For peer review only

regressive cuts to LA budgets have led to reduced spending on early childhood education and care, and other prevention services(16). Whilst increases in unemployment during the recession were dispersed across all parts of the country, changes in welfare provision and cuts to prevention have disproportionately affected deprived areas (17). If these changes are leading to increased incidence of child abuse and neglect, we would expect CLA rates to rise more rapidly in more deprived areas.

There are stark differences in rates of CLA across LAs in England (1). Less clear is how these are changing over time. Our aim in this study is to determine whether the rate of children becoming looked after increased more in deprived areas of the country, after controlling for unemployment – so parcelling out the effects of the recession itself from the effects of other possible drivers of changing inequalities. We further quantify trends in inequalities in children experiencing other forms of child welfare intervention, in order to assess whether findings for CLA are consistent across child welfare outcomes.

METHODS

Data sources and measures

We undertook a longitudinal, local area ecological analysis of CLA rates in England. We used routinely available data from 150 upper-tier LAs between 2004 and 2019, based on 2010 boundaries (see appendix 1). Two LAs, the City of London and the Isles of Scilly, were excluded due to their small population size.

Our primary outcome of interest was the annual age-standardised rate of children becoming looked after by LAs in England (hereafter referred to as ‘CLA rate’). Panel data for the number of CLA, by age group, were drawn from the ‘children looked after data return’, submitted by LAs to the Department for Education on 31st March annually (2). We refer to the financial year by the latter year throughout. Direct age standardisation was performed using the national population distribution of children.

Secondary outcomes captured the wider population of children known to children’s social care. Figure 1 outlines the different child welfare outcomes. The system has been likened to a ‘funnel’, with a progressively smaller number of children experiencing increasingly acute interventions. We used the annual age standardised rate of children becoming the subject of a Child Protection Plan (‘CPP rate’), and children beginning an ‘episode of need’ (‘CIN rate’). Data for these outcomes between 2010 and 2019 were sourced

For peer review only

from the Children in Need (CIN) Census records of children referred for social care support in England (18). For children on a CPP, a breakdown of numbers by category of abuse were available. Disaggregation by age group was requested via a Freedom of Information request, and obtained for years 2012 to 2019.

As a measure of SEC, we used the income deprivation score of the 2010 Indices of Multiple Deprivation (19). This is a non-overlapping count of individuals who, as a result of low earnings, qualify for means-tested benefits, as a proportion of the total population (20). We used 2010 scores based on 2008 data, collected prior to the implementation of austerity policies, to avoid conflating the time-invariant measure of deprivation with unmeasured time-varying exposures that may be changing in response to austerity policies, and so contributing to changing inequalities. In descriptive analyses, we categorised the income deprivation score, assigning LAs to quintiles such that 20% of the 2008 child population was apportioned to each quintile. In regression models, we used a continuous measure of the income deprivation score, converted to a weighted rank by assigning a value from 0 to 1 based on the midpoint of the LA's range in the cumulative distribution. When using this value as a continuous exposure variable in the regression model, the estimated coefficient expresses the change in the Slope Index of Inequality (SII), a commonly used indicator of the association between health outcomes and socioeconomic deprivation (21). The same value can be used to derive the change in the Relative Index of Inequality (RII) when the outcome variable in the regression model is log-transformed and the estimated coefficient exponentiated. In our statistical analyses, the SII represents the absolute difference, and the RII the relative difference, in child welfare outcomes between the LA of lowest and highest level of income deprivation, taking into account the distribution of the child population across LAs (22).

Our analyses also included LA unemployment rates as a covariate in order to separate out the impact of the recession on child welfare outcomes, and so determine whether changes in inequalities were independent of the effects of unemployment. We used data on the number of people claiming Jobseeker's Allowance, plus those claiming Universal Credit who are out of work, as a proportion of residents aged 16-64, in the financial year(23). Although the measure does not capture all unemployment, it is precise and stable at local-area level, is highly correlated with survey-based measures of unemployment (24), and spans the time period of interest. Since the effects of unemployment on child welfare outcomes are unlikely to be immediate, we lagged the variable by one year.

For peer review only

Statistical analysis

First, we assessed descriptive trends for our outcome CLA rate, across LAs grouped into quintiles of income deprivation, between 2004 and 2019. Second, we estimated a segmented linear regression model, with: age-standardised CLA rate as the outcome; year, unemployment rate and income deprivation weighted rank as continuous independent variables; and random intercept and slope terms to account for the correlation between measurements within LAs. Based on our initial descriptive analysis, we included a linear spline for the effect of calendar year, with one knot indicating the timing of the change in trend. We used an iterative search procedure to confirm the knot position resulting in the model with the smallest Bayesian Information Criterion value (25,26). We included an interaction between the spline terms for the effect of year and deprivation to allow for potential differences in trend by SEC. Full details are provided in appendices 2-3.

We used this model to assess whether there was a significant change in the trend in CLA over this period, whether this differed by level of LA income deprivation, and the potential contribution of unemployment to trends in our outcome. We estimated all model parameters by maximum likelihood, using generalized likelihood ratio statistics to compare nested models, and Wald statistics to test hypotheses about model parameters. Similar models were fitted for each of our secondary outcomes, CPP and CIN rates, across years for which data were available, 2012-2019 – based on our descriptive analysis no linear splines were included in these models. Models were estimated using the lme4 package (27), in R version 3.5.1. We carried out supplementary analyses, assessing descriptive trends for all outcomes stratified by age, and, for CPP, by category of abuse (appendices 4-5), and making predictions based on the model (appendix 6). Finally, we fit a model with log-transformed values of the age standardised CLA rate as the outcome in order to derive the RII, and assess trends in relative, as well as absolute inequalities (appendix 7).

Patient and Public Involvement

The research question was informed by early conversations with policymakers and practitioners in the Merseyside area, and reflects the evidence needs identified by senior leaders within Children's Social Care in a priority-mapping exercise facilitated by the What Works Centre for Children's Social Care (28). Early plots were shared with local contacts, and the ensuing discussions informed our hypotheses about drivers of recent trends, in particular age-stratified trends. These hypotheses have informed our research agenda.

For peer review only

RESULTS

Trends in child welfare outcomes

Figure 2 shows CLA rate, by LA income deprivation quintile. Between 2004 and 2008, overall CLA rates dipped slightly: a small increase in the most affluent quintile was offset by decreases in more deprived areas. In 2008, the absolute difference in CLA rate between most and least deprived quintiles was 144 per 100,000 (95% CI 104-184). From around 2008, there was a change in trend and CLA rates rose. A social gradient in CLA is apparent throughout, with the absolute difference between most and least deprived quintiles rising to 174 per 100,000 (95% CI 127-221) in 2019, an increase of 21% from 2008.

Figure 3 shows the CPP and CIN rates. As with CLA rates, CPP rates have risen since 2012, and show a clear social gradient. However, the increase occurred relatively evenly across all groups of LAs, in all age groups. CIN rates also exhibit a social gradient, but trends appear to be relatively stable over time.

Supplementary analyses (appendix 4, appendix figures 1-3) show that the gap in CLA rates between most and least deprived quintiles differed by age. The gap is wide, but relatively stable over time in the youngest age group, children under 1. The gap is widening in the oldest age group, those aged 16-17. Finally, stratifying CPP rates by category of abuse complicates the overall picture of an even rise in rates across all LA income quintiles: we uncovered a widening gap between most and least deprived areas in rates of children becoming subject to a CPP due to concerns about emotional abuse (appendix 5, appendix figure 4).

Segmented linear regression models

Tables 1-2 summarise the results of the segmented regression analyses. For full model output and residual diagnostics, see appendices 7-8 (appendix figures 5-16). For CLA, a knot in 2007, ahead of the 2008 change in trend identified in our descriptive analysis, resulted in the best model fit, indicating a change in trend at this point (appendix 3, appendix figure 17). In our model, rising unemployment in the wake of the financial recession was independently associated with rising CLA rates: for each percentage point increase in the unemployment rate, an estimated additional 9 children per 100,000 (95% CI 6-11) per year entered care the following year. There were no associations between CPP and CIN rates and unemployment rates.

For peer review only

Table 1: Association between outcomes and unemployment rate

Outcome and time period	Annual change (in children per 100,000) for a 1% increase in the unemployment rate the previous year [95% CI]
**CLA rate,** 2004-19	9.0 [6.5, 11.4]
**CPP rate,** 2012-19	-10.4 [-22.2, 1.4]
**CIN rate,** 2012-19	68.5 [-3.1, 140.1]

But unemployment rates do not account for differences in trends between more and less deprived LAs. In 2004, after controlling for LA unemployment, the SII was 193. This captures the absolute inequalities gap across the distribution of LAs on the basis of area deprivation, indicating that there were 193 more CLA per 100,000 in the most deprived LA, compared to the least deprived (95% CI 140-246). Between 2004 and 2007, this gap declined by 11 children per 100,000 per year (95% CI 0-22) (table 2). From 2007 there was a significant change in the trend in inequalities: the gap increased by 15 children per 100,000 per year (95% CI 4-26) relative to the previous trend. Relative inequalities follow the same trend (appendix 7). Altogether, based on our model, we estimate that an additional 18,567 (95% CI 3,553 - 33,394) children were taken into care between 2007 and 2019, than would have been expected had the rise from 2007 occurred in more deprived LAs as it did in the median LA (appendix 6, appendix figure 18).

Table 2: Trends in the Slope Index of Inequality across child welfare outcomes

Outcome and time period	Annual change (in children per 100,000) in the Slope Index of Inequality [95% CI]
**CLA**	
2004-07	-11.4 [-22.3, -0.5]
2007-19, relative to previous trend	14.9 [3.6, 26.2]
**CPP**	
2012-19	4.4 [-11.2, 20.0]
**CIN**	
2012-19	47.1 [-62.7, 156.9]

DISCUSSION

Main findings

For peer review only

The dramatic rise in CLA in England since 2008 has been greater in poorer areas of the country, increasing inequalities. Overall an additional 18,567 (95% CI 3,553 - 33,394) children were taken into LA care between 2007 and 2019 than would be expected if the rise from 2007 had occurred more evenly across LAs. These findings cannot be explained by local economic trends, and are consistent with our hypothesis that austerity measures may have contributed to rising rates of child welfare outcomes. Our analysis also shows that the rise in CLA was associated with rising unemployment at LA level, a marker of the recession.

Trends in inequalities in CLA are not simply mirroring broader trends throughout the 'funnel' of children's social care. Whilst CPP rates are also rising, and show a clear social gradient, we did not find a greater increase in more deprived compared to less deprived areas for children becoming the subject of a CPP and beginning an episode of need.

Several studies have described trends in child welfare outcomes or child maltreatment in the UK. These support our finding of a change in trend and rising rates from around 2007-08 (29) and add context, demonstrating that the turn has followed a thirty-year decline in overall rates – though the rise in CPPs due to neglect and emotional abuse have been rising since the 1990s (30). However, to our knowledge no studies have yet documented trends in inequalities. Paul Bywaters and colleagues at the Child Welfare Inequalities Project began producing evidence of persistent and systematic inequalities in child welfare outcomes in the UK beginning in 2015 (3). This longitudinal analysis of inequalities is indebted to their work.

Strengths and limitations

This study is the first to quantify inequalities in child welfare outcomes longitudinally. A strength is that it uses routinely available data for the whole of England, and explores several child welfare outcomes in order to describe trends throughout the child welfare system.

There are several important study limitations. Due to the lack of individual level data, we used an ecological area-level analysis, and cannot identify whether children entering care were directly affected by income deprivation and unemployment. Conceptually, our portrayal of children's social care as a funnel reflects a theoretical model of how a well-functioning system might operate (figure 1), and may not reflect the trajectory of many individual children and families experiencing child welfare intervention. The association between income deprivation and unemployment rates and child welfare outcomes in our analysis may be due to trends in unobserved time-varying confounding factors that varied between LAs.

For peer review only

Trends in the data reflect the interaction between underlying need and children's services response and we interpret our findings in this light, with caution. Previous analyses by Bywater and colleagues demonstrated the existence of an "inverse intervention law" in child welfare outcomes: a greater risk of intervention in affluent compared to deprived LAs for the same level of neighbourhood deprivation (31), despite lower overall intervention rates. Our models at the level of LAs do not account for the inverse intervention law or rising thresholds reported in more deprived areas. However, this must add weight to our findings: insofar as they reflect changing underlying need, our estimates of the SII are likely to be highly conservative.

Potential explanations of our findings

Changing thresholds

Several changes during this time period may have influenced thresholds for intervention. Firstly, the death by violence of baby Peter Connelly occurred in 2007, when we see a change in the trend of CLA in our data (32). Media and political narratives that emerged in the aftermath of his death centred on the failure of children's services to intervene (33), and ensuing reports by The Children and Family Court Advisory and Support Service note a 'Baby P effect', a marked, short-term rise in applications for care orders in a risk-averse environment (6). This likely accounts for some of the change in trend and initial rise in CLA rates from 2007. Others have argued that a greater policy focus on early intervention and adoption in order to improve outcomes for children experiencing adversity has led to a more interventionist, less family-oriented approach (34). Secondly, in 2009 the Southwark Judgement clarified and reinforced LAs' statutory duties in relation to 16-17 year olds presenting to the LA as homeless (8). This, together with a general shift in practice towards regarding adolescents as vulnerable children rather than nascent adults (35), and greater awareness of extra-familial forms of abuse and principles of contextual safeguarding (36), may be contributing to the rising rates of 16-17 year olds across all outcomes. However, these events are unlikely to fully explain the long-term rise in CLA rates disproportionately affecting more deprived areas.

Economic trends

We found evidence of a positive association between unemployment and CLA rates. Though evidence from the UK is scarce, this aligns with Gillham et al.'s finding of a correlation between male unemployment and child physical abuse in Scotland in the early 1990s (37) and

For peer review only

more recent and extensive evidence from the US demonstrating an association between the recession and increased risk of abuse (38–40). The family stress model posits that heightened stress due to adverse SECs may erode mental health and strain domestic relationships, leading to negative parenting behaviours and increased risk of child abuse and neglect. Barr et al.'s study of the mental health impact of the recession lends credence to this theorised mechanism, demonstrating an association between unemployment and mental health problems in the UK over the same period(17). Yet unemployment did not fully explain changes in CLA rates in our analysis, and unemployment rates have fallen rapidly since 2012: unemployment cannot explain the continued increase in CLA after 2012, nor does it explain rising inequalities. Austerity policies subsequent to the initial recession "shock" may have compounded poor outcomes, affecting inequalities in CLA in several ways.

Changes to welfare provision and prevention

Regressive cuts to English LA budgets, with deeper cuts in more deprived areas, have precipitated a shift in expenditure away from prevention towards acute services(16). Between 2011 and 2018, spending on CLA increased by 68% in real terms, whereas spending on early years preventative services (including Sure Start) and non-statutory young people's services fell about 21%. Reports of rising thresholds for early help in more resource constrained settings, have raised concerns that we are 'storing up trouble' for the future(41). A surge in children entering care who might have benefited from early support could explain the greater rise in more deprived LAs. Adolescents may be particularly susceptible to the consequences of austerity, exposed as they are on multiple fronts, not just in the household and schools, but increasingly in the wider community. Combined cuts to welfare benefits, youth services(42), children's mental health services (43), and community policing(44), might disproportionately affect adolescents in more deprived areas, contributing to widening inequalities in this age group.

Changes to welfare benefits have led to rising child poverty, a contributory causal factor in child abuse and neglect (10,15). Averages losses in earning were particularly high in the more deprived West Midlands and the North West (15). The most vulnerable children on the edge of care, living in families already struggling to cope, may be particularly sensitive to changes in welfare benefit provision. In particular, the phased introduction of Universal Credit from 2013, with its monthly payments in arrears, enhanced conditionality and punitive sanctions, may have compounded financial stress (15) and parental mental health (45). This

For peer review only

would increasingly lead to more children entering care in deprived areas, contributing to trends in inequalities uncovered in our study. Further research is needed to investigate the impact of changing LA prevention spend and child poverty on child welfare outcomes.

Policy and practice implications

We demonstrate that the increase in CLA rates from 2007 has been greater in more deprived LAs. Although it is not possible to say what constitutes an appropriate CLA rate (46), a differential rise by LA deprivation that cannot be explained by the recession is consistent with an increase in underlying need fuelled by welfare changes and cuts to prevention services. While anti-poverty social work practice has a crucial role to play in safely reducing CLA rates and inequalities (47), this must be supported by wider policies to address the social conditions of children's lives. At the national level, this must begin with a renewed commitment to ending child poverty. Tightened social security for families with children, and increased funding for LA Children's Services, are safeguarding priorities. At the local level, holding the line on prevention services, amidst statutory pressures, may yield long-term social and economic benefits. Investment in children is key.

For peer review only

Figure legends

Manuscript figures

Figure 1: Description of the Children's Social Care System in England

Figure 2: CLA rates by LA income deprivation quintile, 2004-2019, with 95% CIs

Figure 3: CPP and CIN rates by LA income deprivation quintile, 2012-19, with 95% CIs

Appendix figures

Appendix figure 1: CLA rates by LA income deprivation quintile, stratified by age

Appendix figure 2: CPP rates by LA income deprivation quintile, stratified by age

Appendix figure 3: CIN rates by LA income deprivation quintile, stratified by age

Appendix figure 4: CPP rates by LA income deprivation quintile, stratified by category of abuse

Appendix figure 5: CLA model - absolute inequalities - histogram of standardised residuals

Appendix figure 6: CLA model - absolute inequalities - quantile quantile plot

Appendix figure 7: CLA model - absolute inequalities - comparing observed and predicted rates

Appendix figure 8: CLA model - relative inequalities - histogram of standardised residuals

Appendix figure 9: CLA model - relative inequalities - quantile quantile plot

Appendix figure 10: CLA model - relative inequalities - comparing observed and predicted rates

Appendix figure 11: CPP model - histogram of standardised residuals

Appendix figure 12: CPP model - quantile quantile plot

Appendix figure 13: CPP model - comparing observed and predicted rates

Appendix figure 14: CIN model - histogram of standardised residuals

Appendix figure 15: CIN model - quantile quantile plot

Appendix figure 16: CIN model - comparing observed and predicted rates

For peer review only

Appendix figure 17: CLA model - breakpoint analysis

Appendix figure 18: CLA model - predictions based on the model

For peer review only

Funding

DB, DT-R, BB, KEM and AA are funded by the National Institute for Health Research School for Public Health Research (SPHR), Grant Reference Number PD-SPH-2015. SW is funded by a Wellcome Trust Society and Ethics Research Fellowship (200335/Z/15/Z). DT-R and ETCL are funded by the Medical Research Council (MRC) on a Clinician Scientist Fellowship (MR/P008577/1). BB is also supported by the National Institute for Health Research Applied Research Collaboration North West Coast (ARC NWC). The views expressed in this publication are those of the authors and not necessarily those of the National Institute for Health Research or the Department of Health and Social Care, MRC or Wellcome Trust. The funders of the study had no role in study design, data collection, data analysis, data interpretation, or the writing of the report. The corresponding author had access to all of the data in the study and had final responsibility for the decision to submit for publication.

Declarations of interest

We confirm that the authors have no competing interests to disclose.

Data sharing

The publicly available, aggregate data can be accessed from the Place-based Longitudinal Data Resource (PLDR, https://pldr.org/), and the code is available upon reasonable request.

Ethics

Research Ethics committee approval was not required for this secondary data analysis, which employed publicly available aggregate data.

Contributorship statement

DLB is lead author and guarantor. DT-R and BB are joint senior author. DLB and DT-R planned the study. DLB, BB and DT-R analysed the data, supported by KEM, DKS, SW, ETCL and AA. DLB, DT-R and BB led the drafting and revision of the manuscript. All authors contributed to the interpretation of the data and revision of the manuscript; all authors approved the submitted version of the manuscript.

License statement

For peer review only

I, the Submitting Author has the right to grant and does grant on behalf of all authors of the Work (as defined in the below author licence), an exclusive licence and/or a non-exclusive licence for contributions from authors who are: i) UK Crown employees; ii) where BMJ has agreed a CC-BY licence shall apply, and/or iii) in accordance with the terms applicable for US Federal Government officers or employees acting as part of their official duties; on a worldwide, perpetual, irrevocable, royalty-free basis to BMJ Publishing Group Ltd ("BMJ") its licensees and where the relevant Journal is co-owned by BMJ to the co-owners of the Journal, to publish the Work in Journal of Epidemiology & Community Health and any other BMJ products and to exploit all rights, as set out in our licence.

The Submitting Author accepts and understands that any supply made under these terms is made by BMJ to the Submitting Author unless you are acting as an employee on behalf of your employer or a postgraduate student of an affiliated institution which is paying any applicable article publishing charge ("APC") for Open Access articles. Where the Submitting Author wishes to make the Work available on an Open Access basis (and intends to pay the relevant APC), the terms of reuse of such Open Access shall be governed by a Creative Commons licence – details of these licences and which Creative Commons licence will apply to this Work are set out in our licence referred to above.

For peer review only

REFERENCES

1. Bywaters P. Inequalities in child welfare: Towards a new policy, research and action agenda. British Journal of Social Work. 2015;45(1):6–23.

2. Department for Education. Statistics: looked-after children (Collection) [Internet]. 2013 [cited 2020 Jun 16]. Available from: https://www.gov.uk/government/collections/statistics-looked-after-children

3. Simkiss D. Outcomes for looked after children and young people. Paediatrics and Child Health (United Kingdom). 2012;22(9):388–92.

4. Local Government Association. LGA Budget Submission: Autumn 2017. 2017.

5. O'Donnell M, Scott D, Stanley F. Child abuse and neglect - Is it time for public health approach? Australian and New Zealand Journal of Public Health. 2008;32(4):325–30.

6. CAFCASS. Annual report and accounts 2009-10. Crown; 2010.

7. Featherstone B, Gupta A, Morris K, Warner J. Let's stop feeding the risk monster: Towards a social model of 'child protection.' Families, Relationships and Societies. 2018;7(1):7–22.

8. R (on the application of G) v Southwark London Borough Council 2009.

9. Walsh D, McCartney G, Smith M, Armour G. Relationship between childhood socioeconomic position and adverse childhood experiences (ACEs): A systematic review. Vol. 73, Journal of Epidemiology and Community Health. BMJ Publishing Group; 2019. p. 1087–93.

10. Bywaters P, Bunting L, Davidson G, Hanratty J, Mason W, McCartan C, et al. The relationship between poverty, child abuse and neglect: an evidence review. 2016.

11. Hillson JMC, Kuiper NA. A stress and coping model of child maltreatment. Clinical Psychology Review. 1994;14(4):261–85.

12. Cancian M, Yang MY, Slack KS. The effect of additional child support income on the risk of child maltreatment. Social Service Review. 2013;87(3):417–37.

13. HM Treasury. Budget 2010. London; 2010.

14. Department for Work & Pensions. 2010 to 2015 government policy: welfare reform - GOV.UK [Internet]. 2010 [cited 2020 Jun 16]. Available from: https://www.gov.uk/government/publications/2010-to-2015-government-policy-welfare-reform/2010-to-2015-government-policy-welfare-reform

15. Tucker J. The Austerity Generation: the impact of a decade of cuts on family incomes and child poverty [Internet]. Child Poverty Action Group. 2017. Available from: http://www.cpag.org.uk/sites/default/files/Austerity Generation FINAL.pdf

16. Webb CJR, Bywaters P. Austerity, rationing and inequity: trends in children's and young peoples' services expenditure in England between 2010 and 2015. Local Government Studies. 2018 May 4;44(3):391–415.

17. Barr B, Kinderman P, Whitehead M. Trends in mental health inequalities in England during a period of recession, austerity and welfare reform 2004 to 2013. Social Science and Medicine. 2015;147:324–31.

18. Emmott EH, Jay MA, Woodman J. Cohort profile: Children in Need Census (CIN) records of children referred for social care support in England. BMJ Open. 2019;9(2).

For peer review only

19. Ministry of Housing Communities & Local Government. English Indices of Deprivation 2010 (Official Statistics).

20. Department for Communities and Local Government. The English Indices of Deprivation 2015: Technical report. 2015.

21. Straatmann VS, Lai E, Lange T, Campbell MC, Wickham S, Andersen AMN, et al. How do early-life factors explain social inequalities in adolescent mental health? Findings from the UK Millennium Cohort Study. Journal of Epidemiology and Community Health. 2019;73(11):1049–60.

22. Regidor E. Measures of health inequalities: Part 2. Journal of Epidemiology and Community Health. 2004;58(11):900–3.

23. ONS. NOMIS - official labour market statistics 2019.

24. Barr B, Taylor-Robinson D, Scott-Samuel A, McKee M, Stuckler D. Suicides associated with the 2008-10 economic recession in England: Time trend analysis. BMJ (Online) [Internet]. 2012 Sep 8 [cited 2020 Jul 30];345(7873). Available from: https://www.bmj.com/content/345/bmj.e5142

25. Taylor-Robinson D, Lai ETC, Wickham S, Rose T, Norman P, Bambra C, et al. Assessing the impact of rising child poverty on the unprecedented rise in infant mortality in England, 2000-2017: time trend analysis. BMJ Open. 2019;9(10).

26. Barr B, Higgerson J, Whitehead M. Investigating the impact of the English health inequalities strategy: Time trend analysis. BMJ (Online). 2017;358.

27. Bates D, Mächler M, Bolker BM, Walker SC. Fitting linear mixed-effects models using lme4. Journal of Statistical Software. 2015;67(1).

28. Jones L, Scholten C, Whelan E, Clayton V, Sanders M. What Matters For What Works?: Setting research priorities for what works for Children's Social care. 2020.

29. Chandan JS, Gokhale KM, Bradbury-Jones C, Nirantharakumar K, Bandyopadhyay S, Taylor J. Exploration of trends in the incidence and prevalence of childhood maltreatment and domestic abuse recording in UK primary care: a retrospective cohort study using "the health improvement network" database. BMJ open. 2020;

30. Degli Esposti M, Humphreys DK, Jenkins BM, Gasparrini A, Pooley S, Eisner M, et al. Long-term trends in child maltreatment in England and Wales, 1858–2016: an observational, time-series analysis. The Lancet Public Health. 2019 Mar 1;4(3):e148–58.

31. Bywaters P, Brady G, Sparks T, Bos E, Bunting L, Daniel B, et al. Exploring inequities in child welfare and child protection services: Explaining the "inverse intervention law." Children and Youth Services Review. 2015;57:98–105.

32. Haringey Local Safeguarding Children Board. Serious Case Review "Child A." 2009.

33. Warner J. "Heads Must Roll"? Emotional Politics, the Press and the Death of Baby P. British Journal of Social Work. 2014;44(6):1637–53.

34. Featherstone B, Morris K, White S. A marriage made in hell: Early intervention meets child protection. British Journal of Social Work [Internet]. 2014 [cited 2020 Jun 19];44(7):1735–49. Available from: https://academic.oup.com/bjsw/article-abstract/44/7/1735/1718126

35. NSPCC. Teenagers: learning from case reviews. 2014.

For peer review only

36. Lloyd J, Firmin C. No Further Action: Contextualising Social Care Decisions for Children Victimised in Extra-Familial Settings. Youth Justice. 2020;20(1–2):79–92.

37. Gillham B, Tanner G, Cheyne B, Freeman I, Rooney M, Lambie A. Unemployment rates, single parent density, and indices of child poverty: Their relationship to different categories of child abuse and neglect. Child Abuse and Neglect. 1998;22(2):79–90.

38. Brooks-Gunn J, Schneider W, Waldfogel J. The great recession and the risk for child maltreatment. Child Abuse and Neglect. 2013;37(10):721–9.

39. Cherry R, Wang C. The link between male employment and child maltreatment in the U.S., 2000-2012. Children and Youth Services Review. 2016;66:117–22.

40. Millett L, Lanier P, Drake B. Are economic trends associated with child maltreatment? Preliminary results from the recent recession using state level data. Children and Youth Services Review. 2011 Jul 1;33(7):1280–7.

41. All Party Parliamentary Group for Children. Storing Up Trouble: a postcode lottery of children's social care. 2018.

42. Kelly E, Lee T, Sibieta L, Waters T. Public Spending on Children: 2000 to 2020. 2018.

43. Children's Commissioner. Briefing: Children's Mental Healthcare in England. 2017.

44. Allen G, Audickas L. Briefing paper: Police Service Strength [Internet]. 2020. Available from: http://researchbriefings.files.parliament.uk/documents/SN00634/SN00634.pdf

45. Wickham S, Bentley L, Rose T, Whitehead M, Taylor-Robinson D, Barr B. Effects on mental health of a UK welfare reform, Universal Credit: a longitudinal controlled study. The Lancet Public Health. 2020;5(3):e157–64.

46. Bywaters P, Scourfield J, Jones C, Sparks T, Elliott M, Hooper J, et al. Child welfare inequalities in the four nations of the UK. Journal of Social Work. 2020;20(2):193–215.

47. Morris K, Mason W, Bywaters P, Featherstone B, Daniel B, Brady G, et al. Social work, poverty, and child welfare interventions. Child and Family Social Work. 2018;23(3):364–72.

For peer review only

The children's social care system has been described as series of 'filters and funnels'[∞]. Using this analogy, through the funnel, successive phases of risk assessment and service response determine a child's incident status:

1. **Referrals**. At the wide end of the funnel are all referrals.
2. **Children in Need**. A child is 'in need' (CIN), if deemed to require additional support in order to achieve a reasonable standard of health and development. An episode of need relates to an open case following a LA's acceptance of a referral to children's social care.
3. **Child Protection Plan**. A Child Protection Plan (CPP) may be drawn up where, following an investigation, concerns persist as to whether a child is suffering, or likely to suffer, significant harm.
4. **Children Looked After** (CLA) are at the narrow end of the funnel, enduring adversity sufficiently severe for the State to intervene in their upbringing[£].

This figure represents the 'funnel' of children's social care, and shows the overlap between incident child welfare status. Size and overlaps are not to scale. Child population estimates are taken from most recent data returns, for the period 2018-19. In rare cases, due to residual safeguarding concerns, a child in care may also be subject to a CPP.

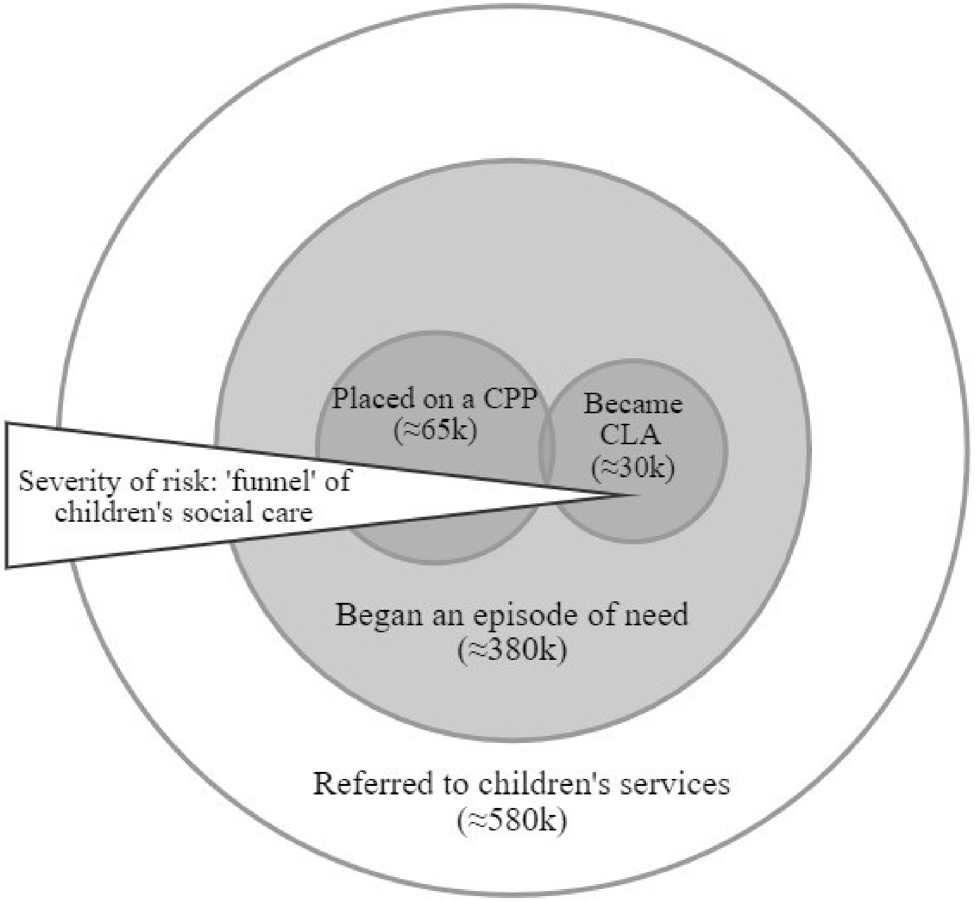

∞ Gibbons J, Bell C, Conroy S. Operating the child protection system: a study of child protection practices in English local authorities. London: HMSO; 1995.
£ Emmott EH, Jay MA, Woodman J. Cohort profile: Children in Need Census (CIN) records of children referred for social care support in England. BMJ Open. 2019;9(2):e023771.

For peer review only - http://bmjopen.bmj.com/site/about/guidelines.xhtml

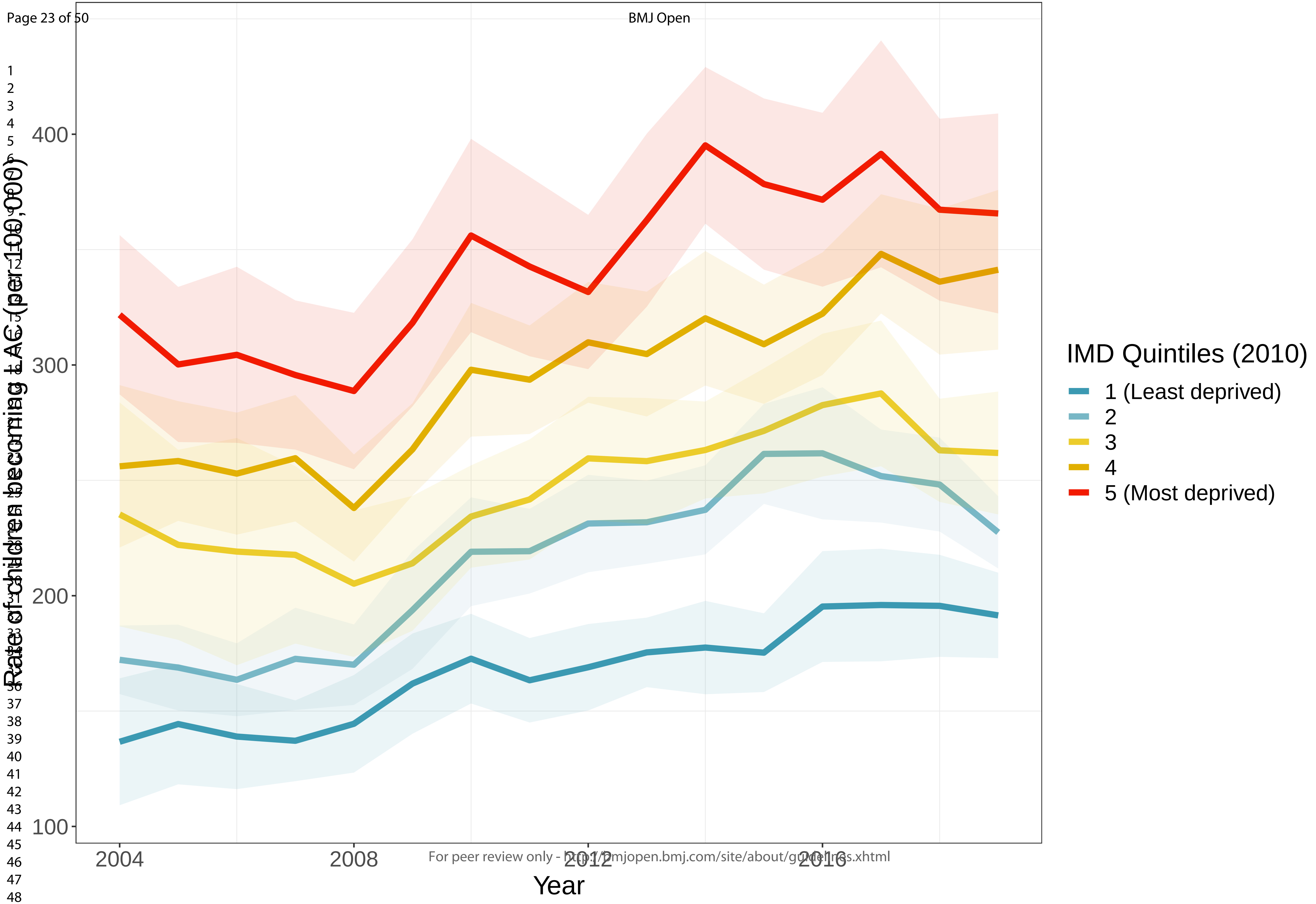
Rate of children becoming LAC (per 100,000)
400
300
200
100
2004
2008
2012
2016
Year
IMD Quintiles (2010)
1 (Least deprived)
2
3
4
5 (Most deprived)

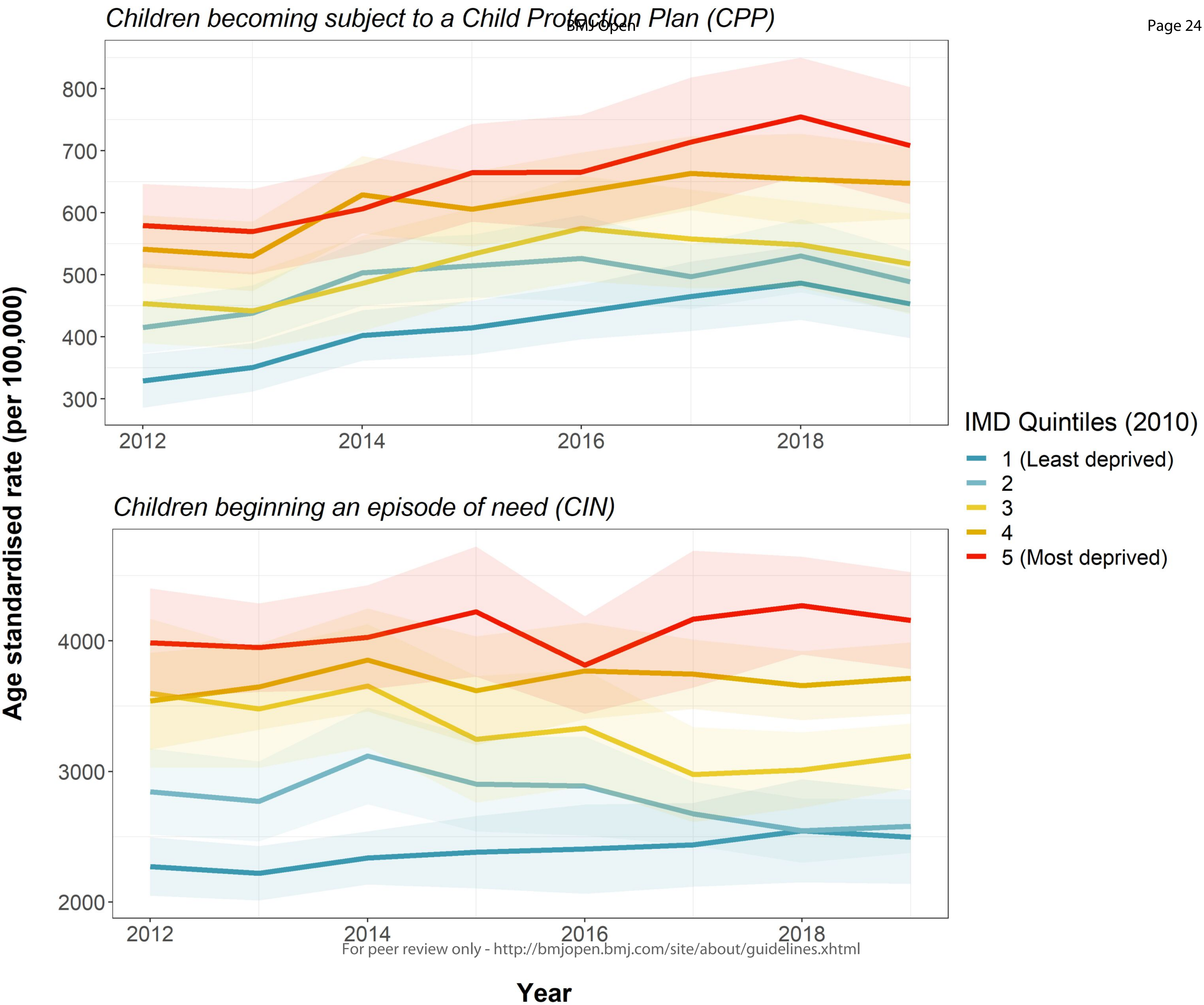
Children becoming subject to a Child Protection Plan (CPP)
800
700
600
500
400
300
2012
2014
2016
2018
Children beginning an episode of need (CIN)
4000
3000
2000
2012
2014
2016
2018
Age standardised rate (per 100,000)
Year
IMD Quintiles (2010)
1 (Least deprived)
2
3
4
5 (Most deprived)

SUPPLEMENTARY MATERIALS

Appendix 1: Harmonising data

Where changes to LA boundaries in 2009 led to the formation of two upper tier unitary authorities from a single county, CLA numbers for preceding years were split between these LAs based on their 2009 child population ratio. In the publicly available data, for reasons of confidentiality, numbers from one to five inclusive were supressed. For each missing value we therefore imputed a random integer in this range. There were only three cases of missing data, across two years, early in the implementation of the CIN census: age stratified data were not available for Havering and Newham in 2012, or Norfolk in 2013. Given the low degree of missingness, we performed complete case analyses.

Appendix 2: Model formulae

Segmented linear regression model for age standardised CLA rate, including linear spline:

$$y_{ij} = \beta_0 + \beta_1 x_{1ij} + \beta_2 x_{2i} + \beta_3 x_{3j} + \beta_4 x_{4j} + \beta_5 x_{3j} x_{2i} + \beta_5 x_{4j} x_{2i} + U_i + V_i x_{3j} + \varepsilon_{ij}$$

Let:

- y_{ij} denote the rate of children taken into care in LA i in year j
- x_{1ij} denote covariate lagged unemployment rate, coded as a continuous variable and dependent on LA i and on year j
- x_{2i} denote the weighted rank of deprivation dependent on LA i, a continuous variable ranging from 0 to 1
- x_{3j} denote the first spline term, which is year j coded as continuous variable and centered at 2004
- x_{4j} denote the second spline term, a continuous variable that takes the value of 0 for year j≤2007, and j−2007 for year j>2007. This defines a segmented regression with knot in 2007.
- $(U_{i,}\ V_i) \sim BVN(0, S_0)$ denote random intercept and slope for LA i
- $\varepsilon_{ij} \sim N\ (0, S_1)$ denote the random error for LA i in year j

Linear regression model for age standardised CPP and CIN rates:

$$y_{ij} = \beta_0 + \beta_1 x_{1ij} + \beta_2 x_{2i} + \beta_3 x_{3j} + \beta_4 x_{3j} x_{2i} + U_i + V_i x_{3j} + \varepsilon_{ij}$$

Let:

- y_{ij} denote the rate of children taken into care in LA i in year j
- x_{1ij} denote covariate lagged unemployment rate, coded as a continuous variable and dependent on LA i and on year j

For peer review only - http://bmjopen.bmj.com/site/about/guidelines.xhtml

- x_{2i} denote the weighted rank of deprivation dependent on LA i, a continuous variable ranging from 0 to 1
- x_{3j} denote year j, coded as continuous variable and centered at 2004
- $(U_{i,}\ V_i){\sim}BVN(0,S_0)$ denote random intercept and slope for LA i
- $\varepsilon_{ij}{\sim}N\ (0,S_1)$ denote the random error for LA i in year j

Appendix 3: Breakpoint analysis

In our model for age standardised CLA rates, we used an iterative search procedure in order to identify which breakpoint offered the best fit. Appendix figure 17 shows the BIC value for each successive breakpoint used in the model. This led us to fit a knot in 2007.

Appendix 4: Age stratified analyses of crude CLA, CPP and CIN rates by LA deprivation quintile

For the age stratified analyses, we calculated rates for each of our outcomes using child population data, broken down by the same age bands available in the routine and FoI data, sourced from the Office for National Statistics (ONS) mid-year population estimates, accessed via Stat-Xplore[1]. These formed our denominator. We plotted rates for all age-stratified outcomes, across years for which data were available, enabling a comparison, by age group, across outcomes. Appendix figure 1 shows that the rise in CLA rates was mainly due to children under the age of 1 and children aged 16-17 entering care. Though wide, the gap in rates between most and least deprived LAs for the youngest age group does not appear to be widening. In the oldest age group however, there is a pronounced increase in the gap from 2010.

We sought to determine whether trends in CLA were reflected through the funnel of children's social care (appendix figures 2-3). The funnel remains widest in children under the age of 1. However, the gap between most and least deprived areas is relatively stable over time regardless of the stage. In children aged 16-17, the funnel narrows considerably from CIN to CPP, then widens once more at the level of CLA. The discontinuity is unique to this age group and may relate to the CPP's focus on risks within the family home. Acute risks to older children are often in the community, from peer groups and criminal networks. This may lead children to be placed directly on a CLA when need becomes acute. The gap in rates

[1] Office for National Statistics. Population Estimates for UK, England and Wales, Scotland and Northern Ireland: Stat-Xplore; 2019 [Available from: https://stat-xplore.dwp.gov.uk].

For peer review only

between most and least deprived areas appears to be widening in both CIN and CLA for this older age group: trends in CLA may well be reflecting, and concentrating, trends in CIN.

Appendix 5: Crude CPP rates by deprivation quintile, stratified by category of abuse

We plotted CPP rates for all categories of abuse, enabling a comparison across categories (appendix figure 4). Neglect, then emotional abuse, are the most commonly recorded primary categories of abuse. Rates for these categories are rising. Where neglect is recorded, the gap in rates between most and least deprived areas appears to have declined slightly from 2014. In contrast, where emotional abuse is recorded, the gap increased dramatically from 2014. Further research is needed to understand how recording practices, child welfare systems, social care practices, and underlying need, may differ by area level income deprivation.

Appendix 6: predictions based on the model

We predict expected CLA rates if the rise in rates from 2007 had occurred in more deprived LAs as it did in the median LA (such that 50% of the 2008 child population live in more deprived areas):

$$y_{ij} = \beta_0 + \beta_1 x_{1ij} + \beta_2 x_{2i} + \beta_3 x_{3j} + \beta_4 x_{4j} + \beta_5 x_{3j} x_{2i} + \beta_5 x_{4j} \boldsymbol{x_5} + U_i + V_i x_{3j} + \varepsilon_{ij}$$

Where $\boldsymbol{x_5}$ denotes the weighted rank of deprivation in the median LA in the cumulative distribution. This scenario preserves the change in trend from 2007 and unemployment rates, but posits that, after controlling for unemployment rates, the change in trend should not disproportionately affect areas based on their levels of income deprivation. Appendix figure 18, showing LAs grouped by quintiles, illustrates predicted rates according to this scenario.

Appendix 7: Full model output[2]

The following tables summarise the full output for each of the models in turn:

- Age standardised CLA rates
- Age standardised CLA rates, log-transformed (results exponentiated)
- Age standardised CPP rates
- Age standardised CIN rates

[2] Hlavac M. stargazer: Well-Formatted Regression and Summary Statistics Tables. R package version 5.2.2.; 2018.

For peer review only - http://bmjopen.bmj.com/site/about/guidelines.xhtml

Model	**CLA, 2004-2019**	**CLA, 2004-2019**	**CPP, 2012-2019**	**CIN, 2012-2019**
Dependent variable	CLA rate (per 100,000)	Log-transformed CLA rate (per 100,000)	CPP rate (per 100,000)	CIN rate (per 100,000)
Intercept	104.31** (71.56, 137.05)	114.41 ** (101.41, 129.07)	365.74** (306.30, 425.19)	2,190.79** (1,798.70, 2,582.88)
Unemployment rate (lagged)	8.95** (6.48, 11.43)	1.04** (1.03, 1.05)	-10.41 (-22.17, 1.36)	68.52 (-3.06, 140.10)
Spline 1	3.43 (-3.41, 10.27)	1.03** (1.01, 1.06)	12.69** (3.09, 22.29)	-6.76 (-74.71, 61.19)
Deprivation	192.93** (140.01, 245.86)	2.51** (2.07, 3.05)	304.12** (198.42, 409.81)	1,637.02** (949.98, 2,324.07)
Spline 2	1.89 (-5.21, 8.99)	1.00 (0.98, 1.03)	4.38 (-11.20, 19.95)	47.08 (-62.71, 156.88)
Spline 1: deprivation	-11.38* (-22.27, -0.49)	-0.94** (-0.90, -0.98)	-	-
Spline 2: deprivation	14.86* (3.55, 26.16)	1.06** (1.01, 1.10)	-	-
Observations	2,400	2,400	1,197	1,195
Log Likelihood	-13,279.74	211.43	-7,599.82	-9,727.10
Akaike Inf. Crit.	26,581.49	-400.87	15,217.63	19,472.20
Bayesian Inf. Crit.	26,645.10	-337.25	15,263.42	19,517.98
Note:	* p < 0.05, ** p < 0.01	* p < 0.05, ** p < 0.01	* p < 0.05, ** p < 0.01	* p < 0.05, ** p < 0.01
		All coefficients are exponentiated		

For peer review only

Appendix 8: residual diagnostics

The residuals from our model are normally distributed. Plotting standard normal quantiles against the data results in a relatively linear pattern. When grouped by quintile, predicted and observed values of CLA rates appear relatively consistent:

a. CLA model (absolute inequalities): see appendix figures 5-7
b. CLA model (relative inequalities): see appendix figures 8-10
c. CPP model: see appendix figures 11-13
d. CIN model: see appendix figures 14-16

For peer review only

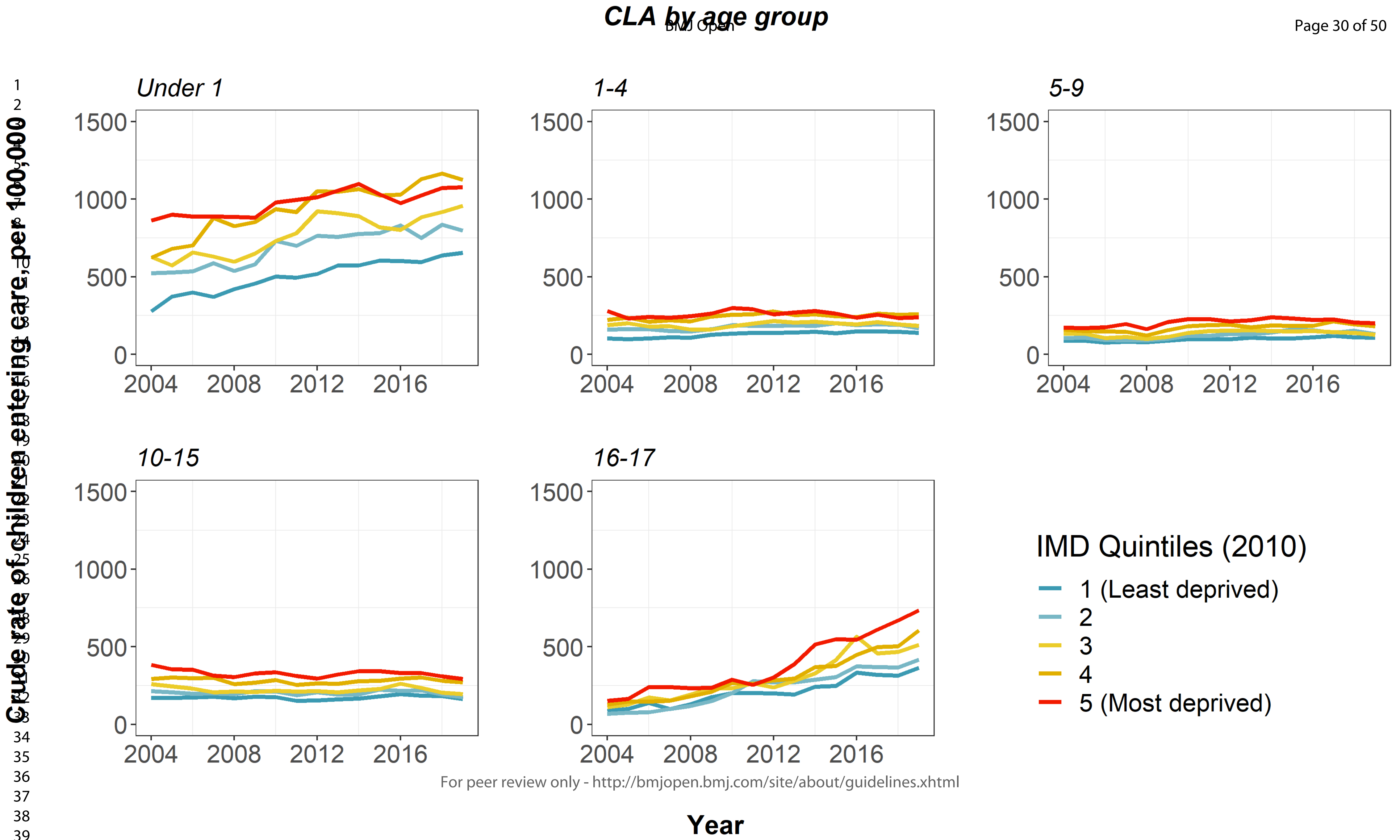
CLA by age group
Under 1
1-4
5-9
10-15
16-17
1500
1000
500
0
2004
2008
2012
2016
Crude rate of children entering care, per 100,000
Year
IMD Quintiles (2010)
1 (Least deprived)
2
3
4
5 (Most deprived)

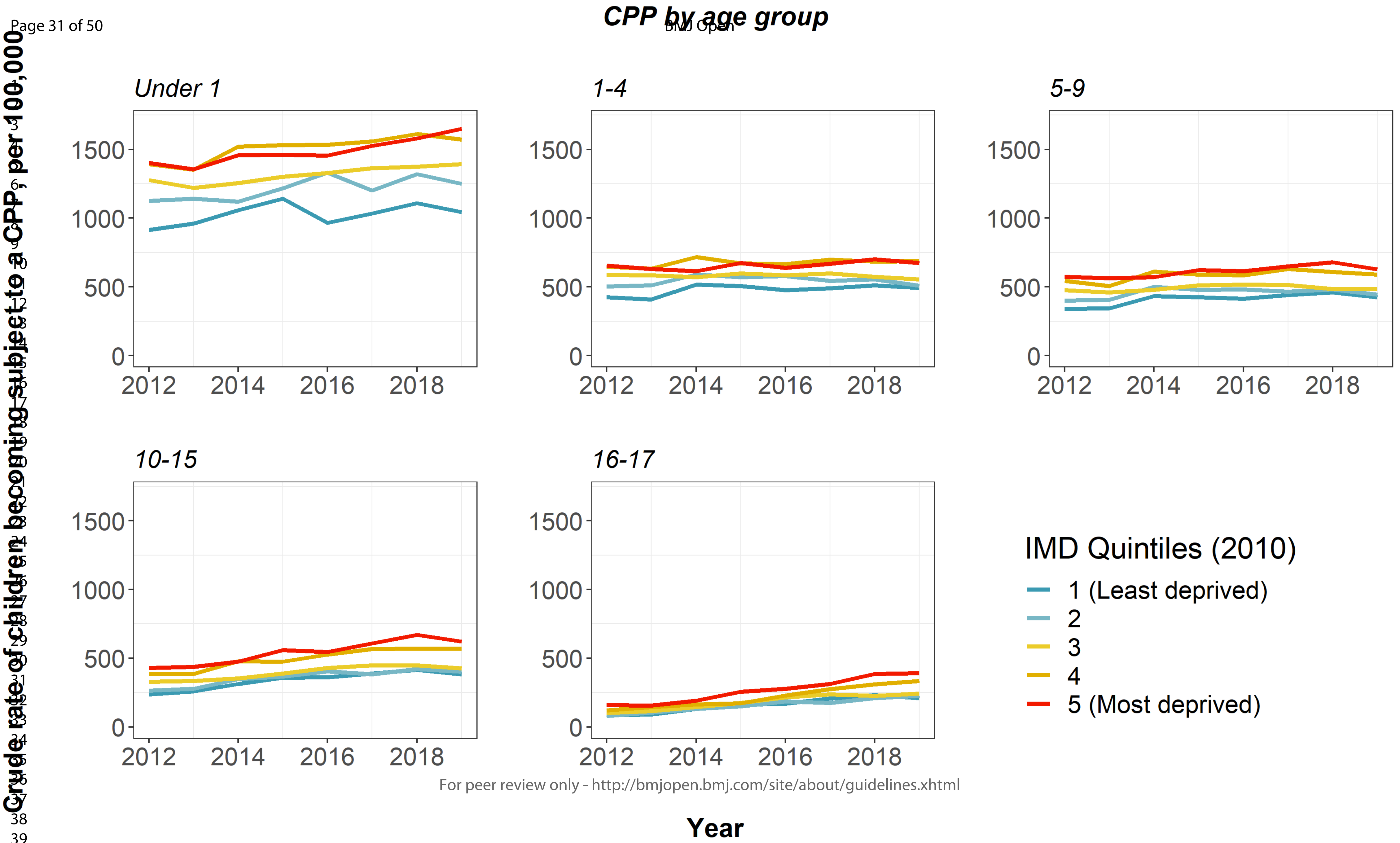
CPP by age group
Under 1
1-4
5-9
10-15
16-17
0
500
1000
1500
2012
2014
2016
2018
Crude rate of children becoming subject to a CPP, per 100,000
Year
IMD Quintiles (2010)
1 (Least deprived)
2
3
4
5 (Most deprived)

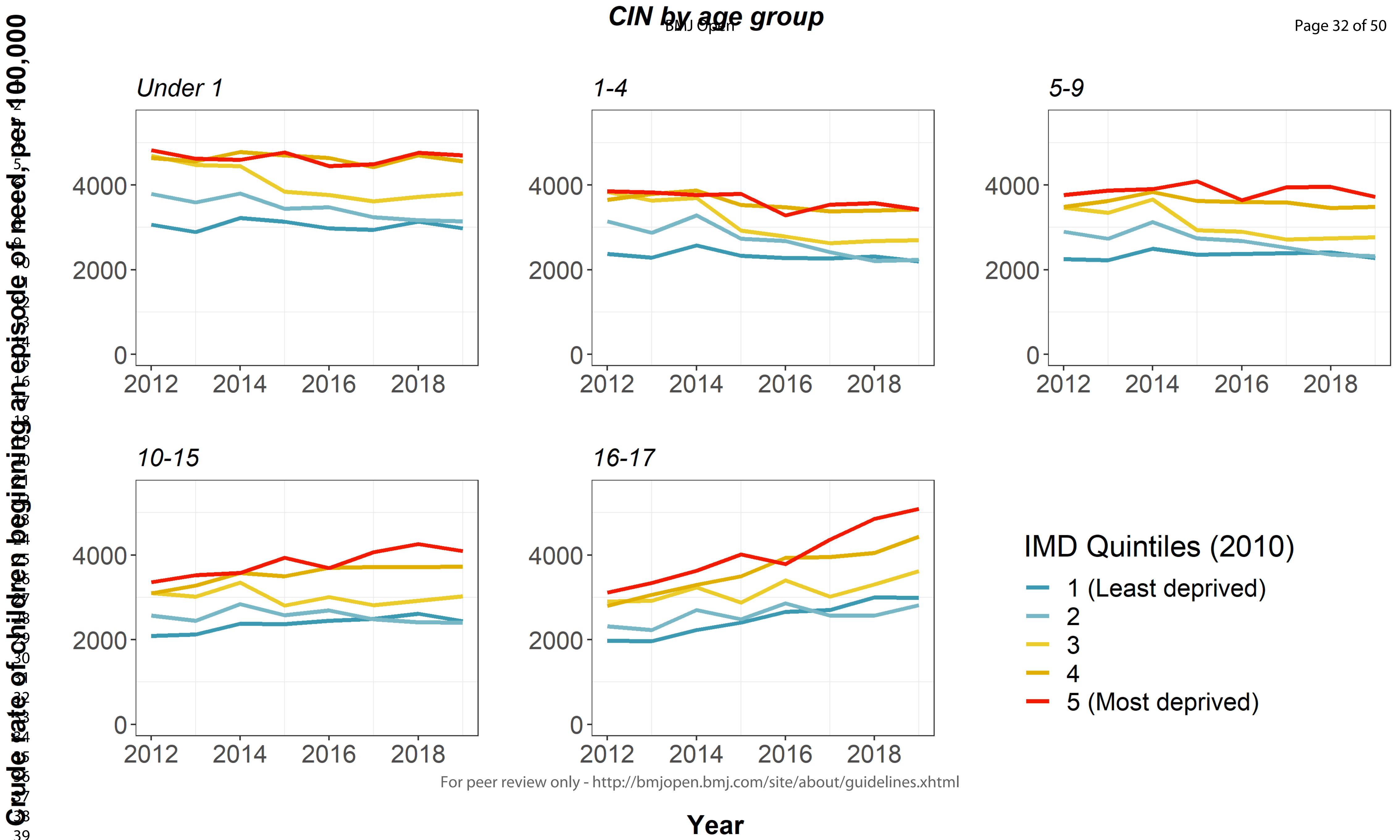
CIN by age group
Crude rate of children beginning an episode of need, per 100,000
Under 1
1-4
5-9
10-15
16-17
0
2000
4000
2012
2014
2016
2018
Year
IMD Quintiles (2010)
1 (Least deprived)
2
3
4
5 (Most deprived)

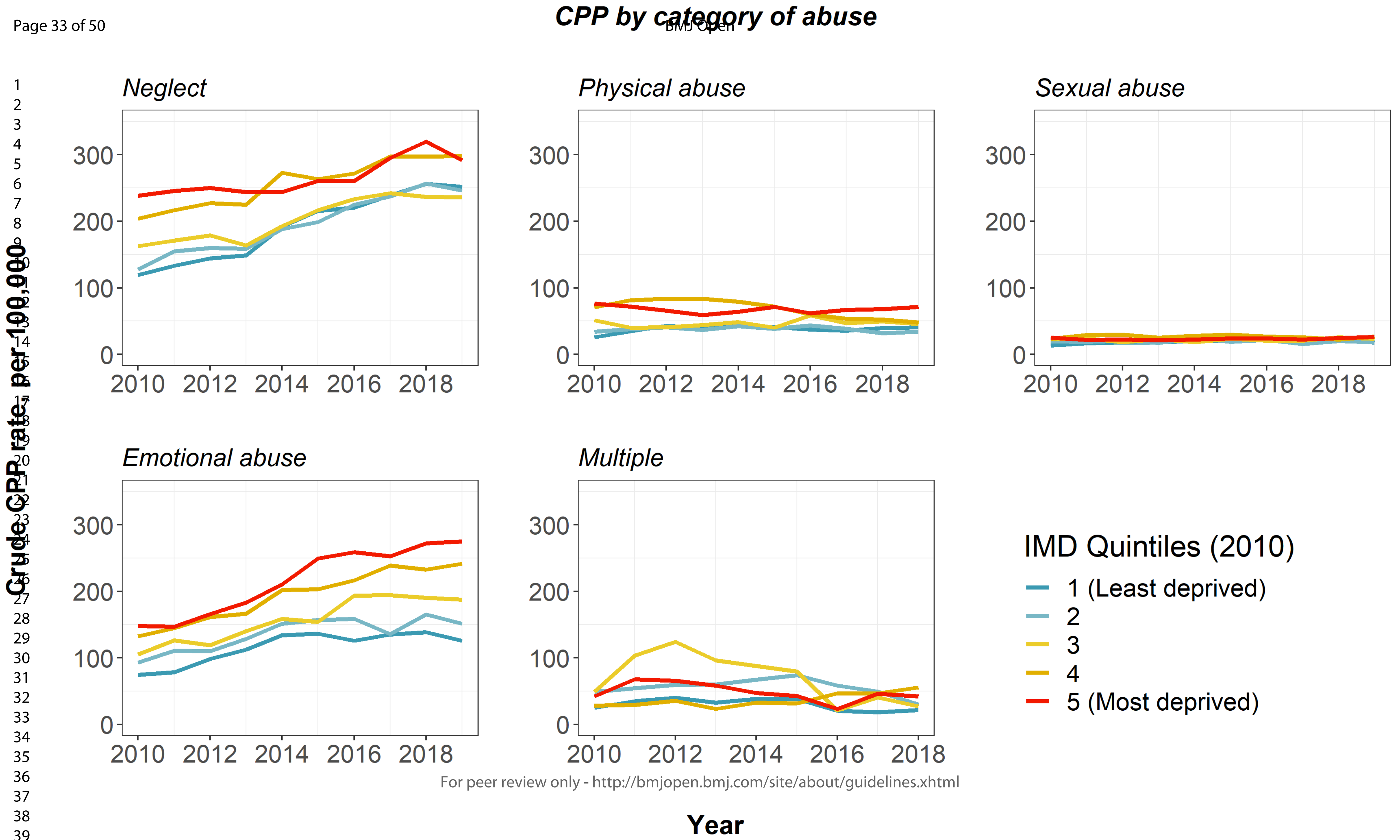
CPP by category of abuse
Neglect
Physical abuse
Sexual abuse
Emotional abuse
Multiple
Crude CPP rate, per 100,000
Year
0
100
200
300
2010
2012
2014
2016
2018
IMD Quintiles (2010)
1 (Least deprived)
2
3
4
5 (Most deprived)

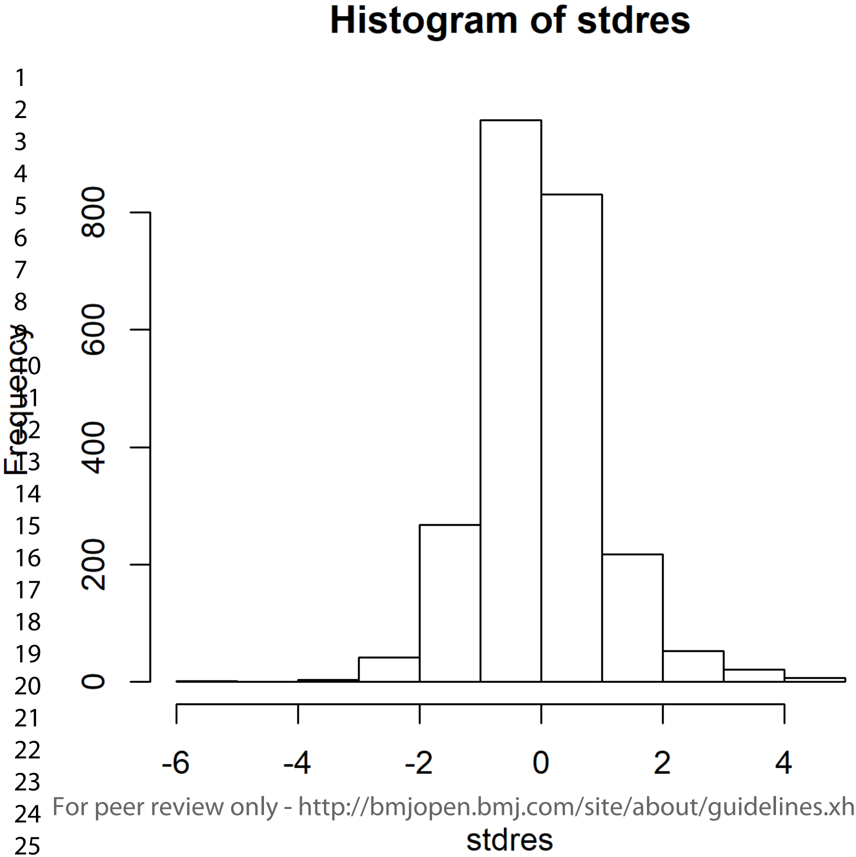
Histogram of stdres
Frequency
0
200
400
600
800
-6
-4
-2
0
2
4
stdres

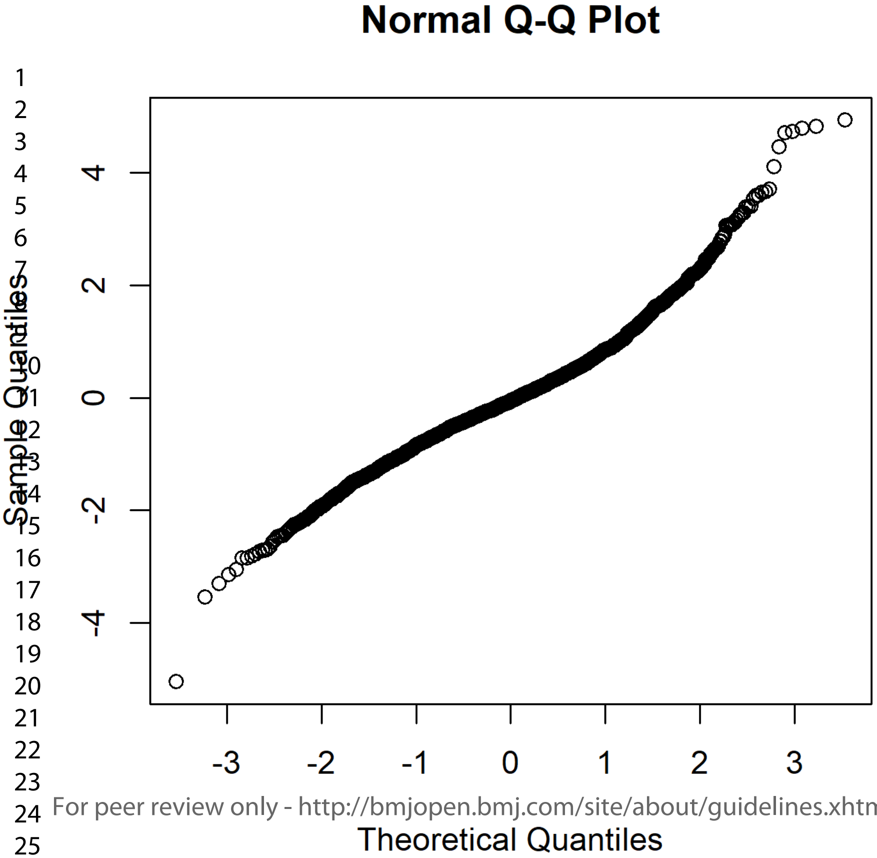

For peer review only - http://bmjopen.bmj.com/site/about/guidelines.xhtml

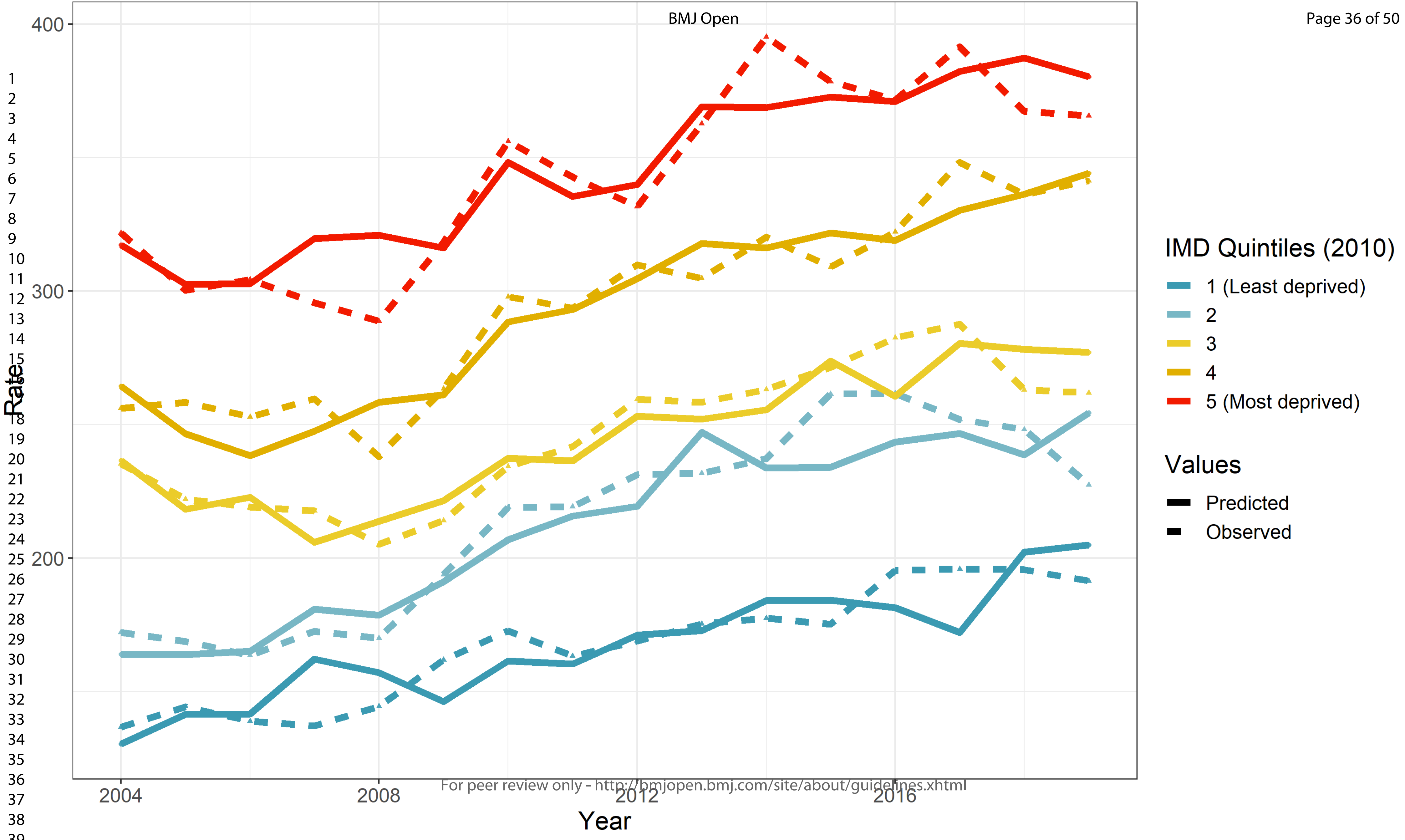
400
300
200
Rate
2004
2008
2012
2016
Year
IMD Quintiles (2010)
1 (Least deprived)
2
3
4
5 (Most deprived)
Values
Predicted
Observed

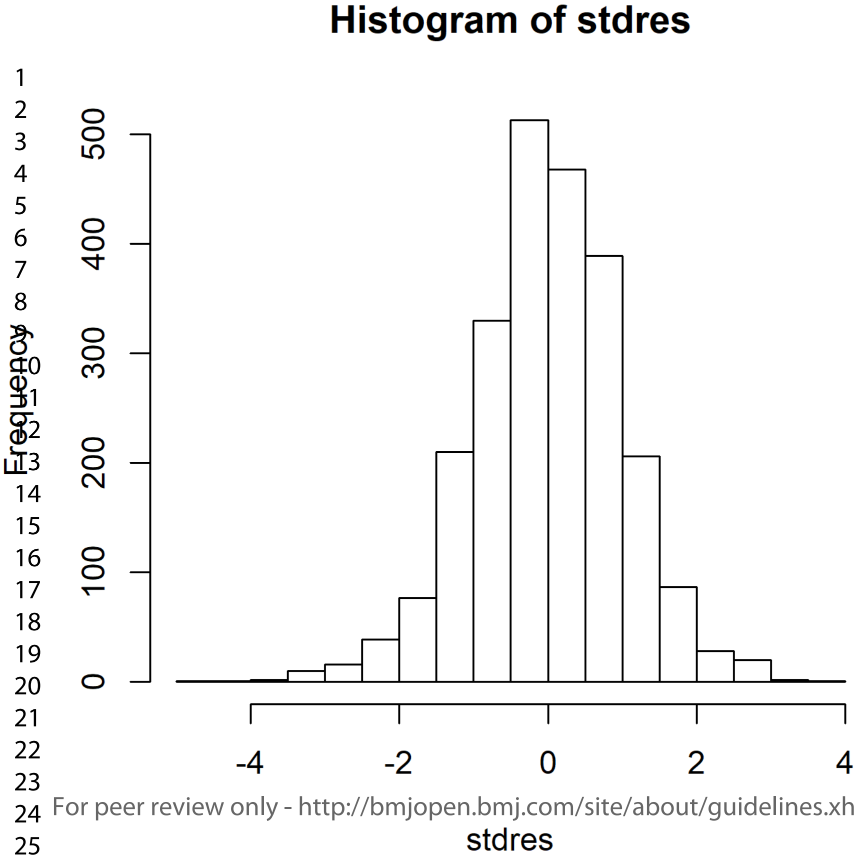

For peer review only - http://bmjopen.bmj.com/site/about/guidelines.xhtml

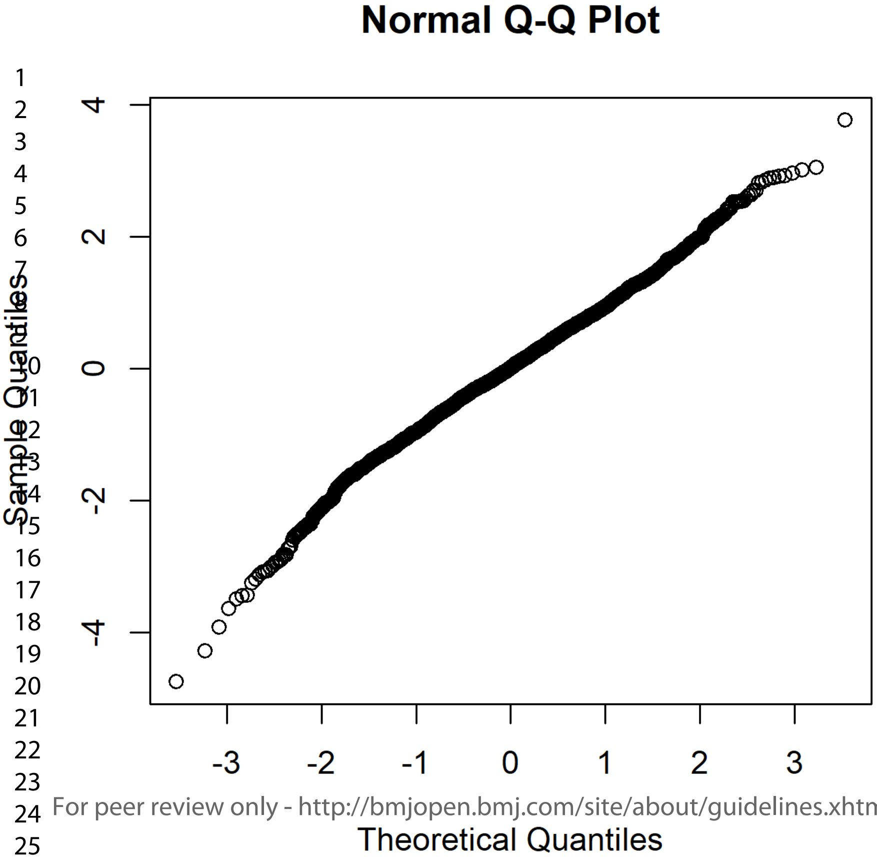

For peer review only - http://bmjopen.bmj.com/site/about/guidelines.xhtml

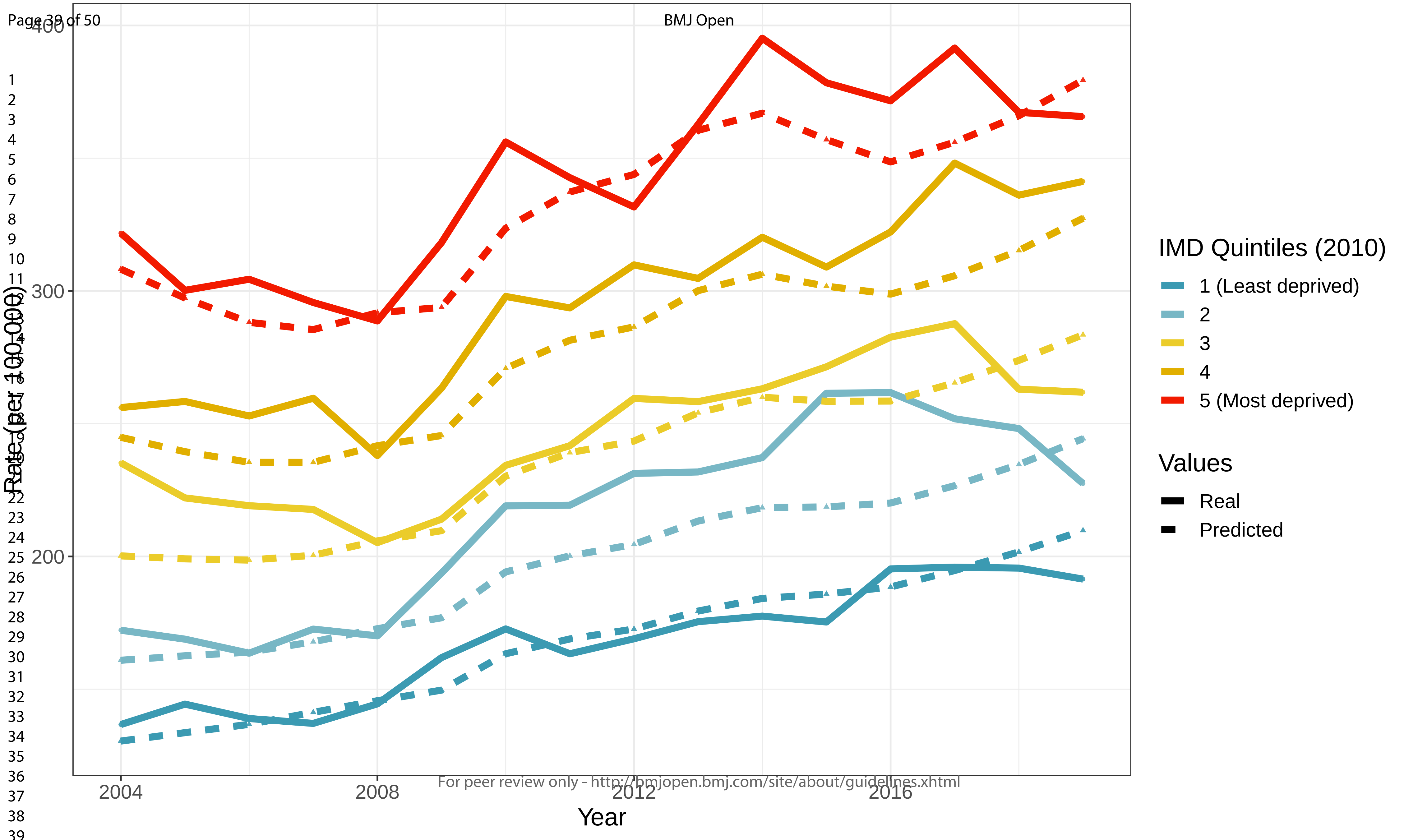
400
300
200
Rate (per 100 000)
2004
2008
2012
2016
Year
IMD Quintiles (2010)
1 (Least deprived)
2
3
4
5 (Most deprived)
Values
Real
Predicted

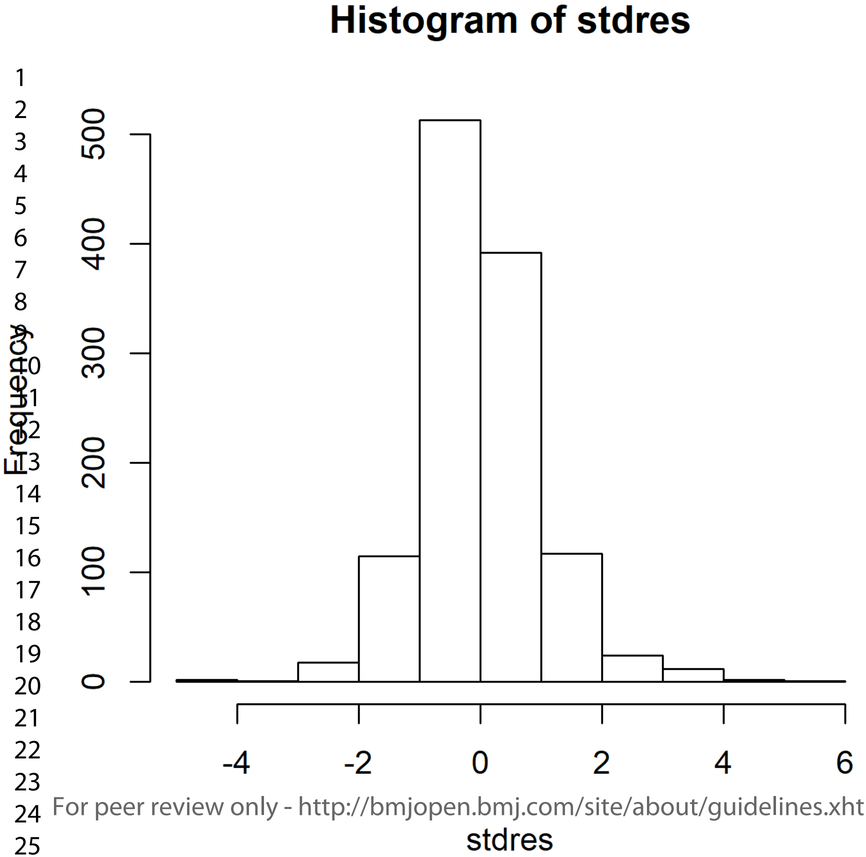

For peer review only - http://bmjopen.bmj.com/site/about/guidelines.xhtml

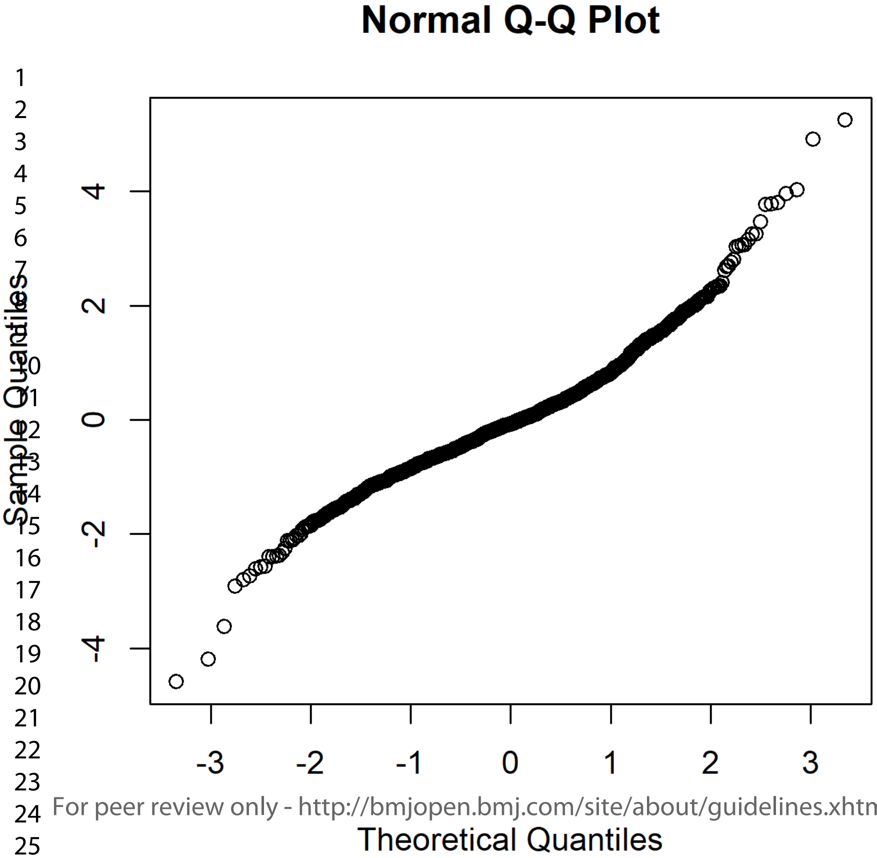

For peer review only - http://bmjopen.bmj.com/site/about/guidelines.xhtml

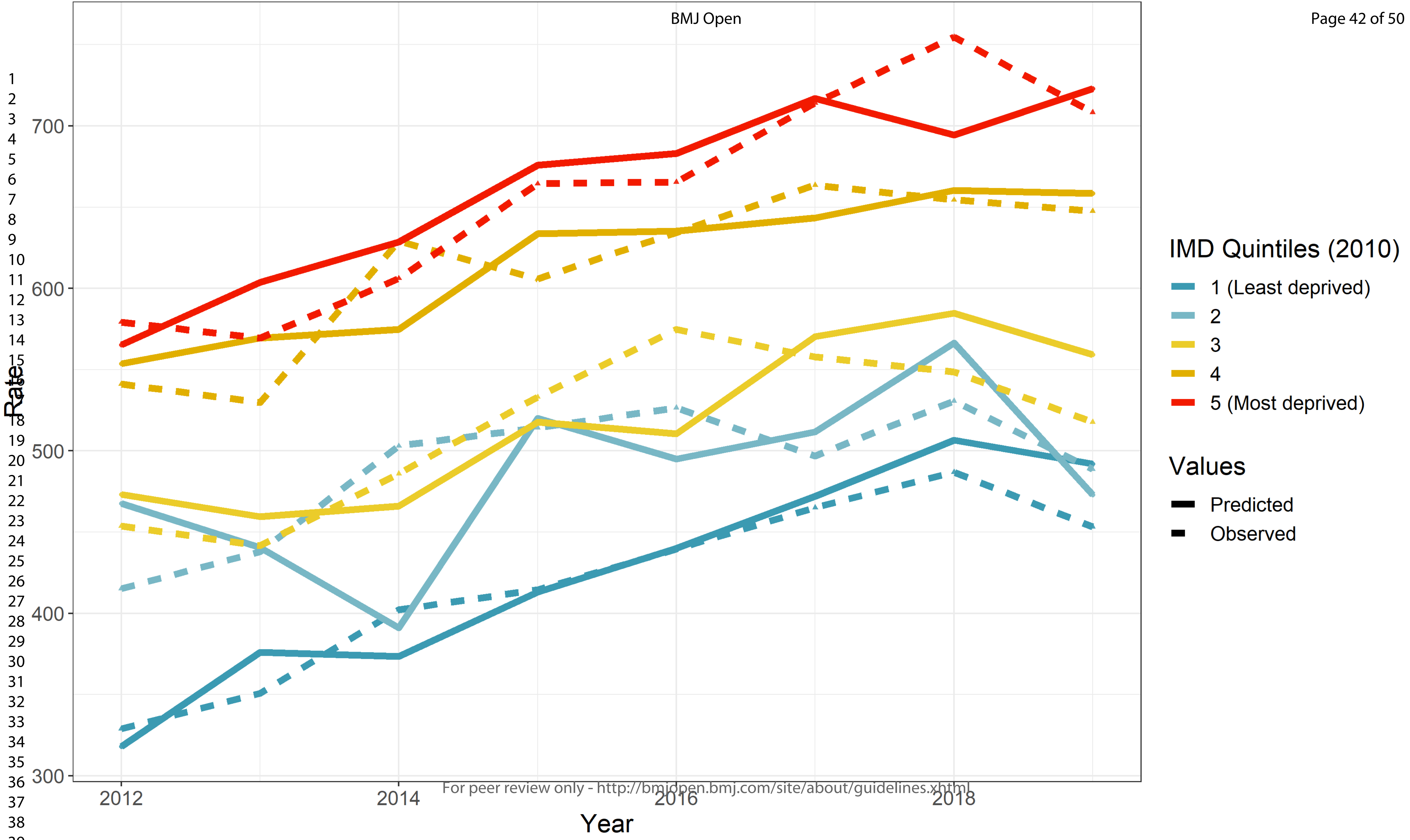
700
600
500
400
300
Rate
2012
2014
2016
2018
Year
IMD Quintiles (2010)
1 (Least deprived)
2
3
4
5 (Most deprived)
Values
Predicted
Observed

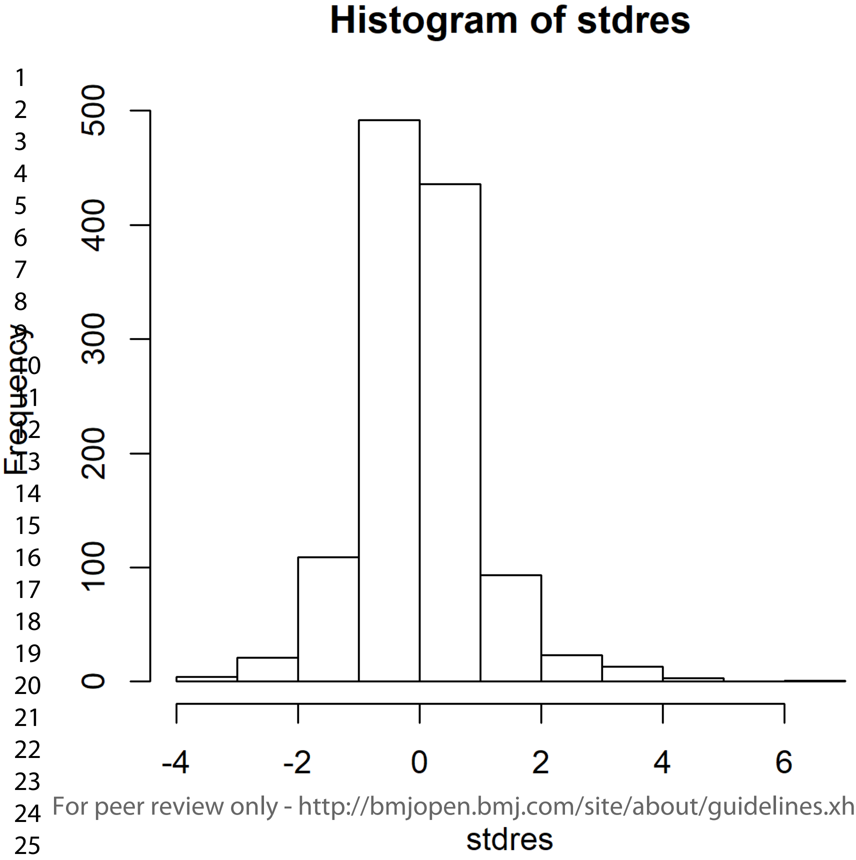
Histogram of stdres
Frequency
0
100
200
300
400
500
-4
-2
0
2
4
6
stdres

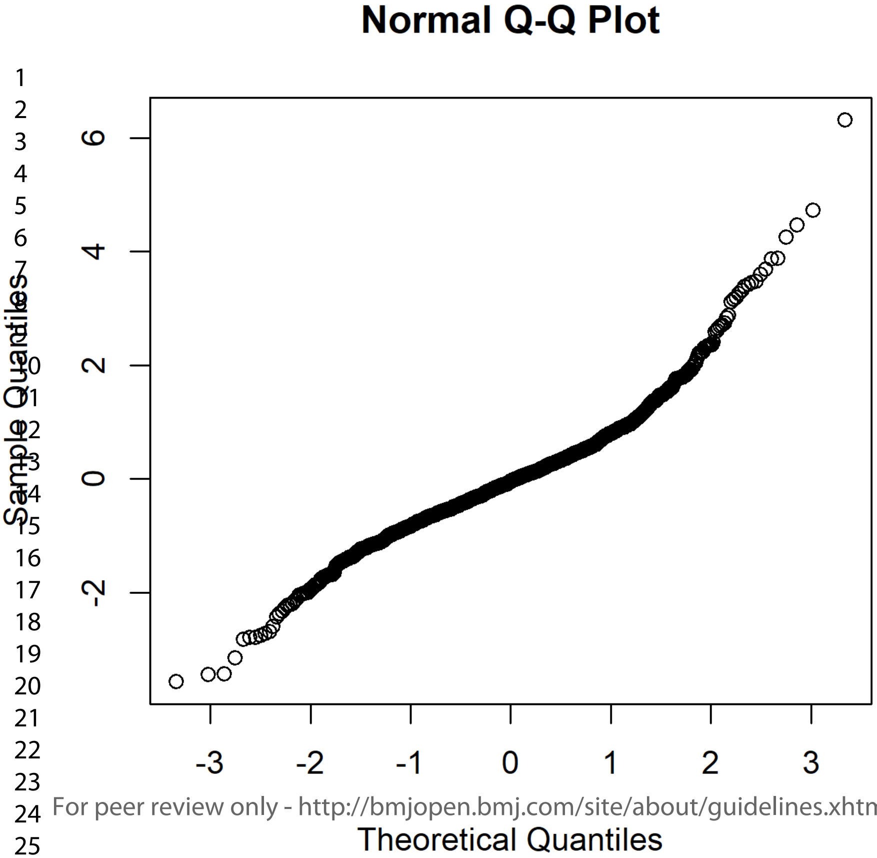

For peer review only - http://bmjopen.bmj.com/site/about/guidelines.xhtml

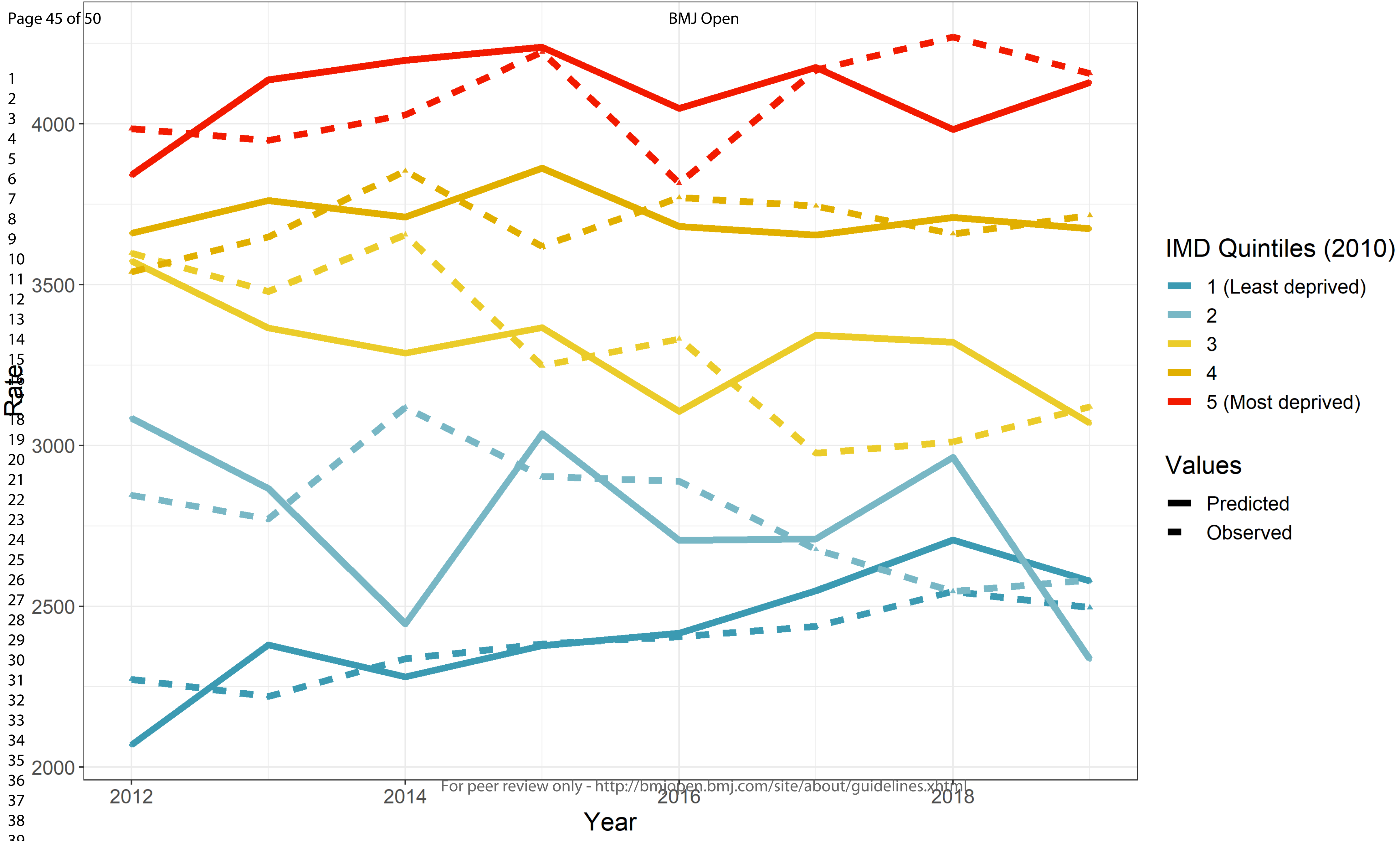
4000
3500
3000
2500
2000
Rate
2012
2014
2016
2018
Year
IMD Quintiles (2010)
1 (Least deprived)
2
3
4
5 (Most deprived)
Values
Predicted
Observed

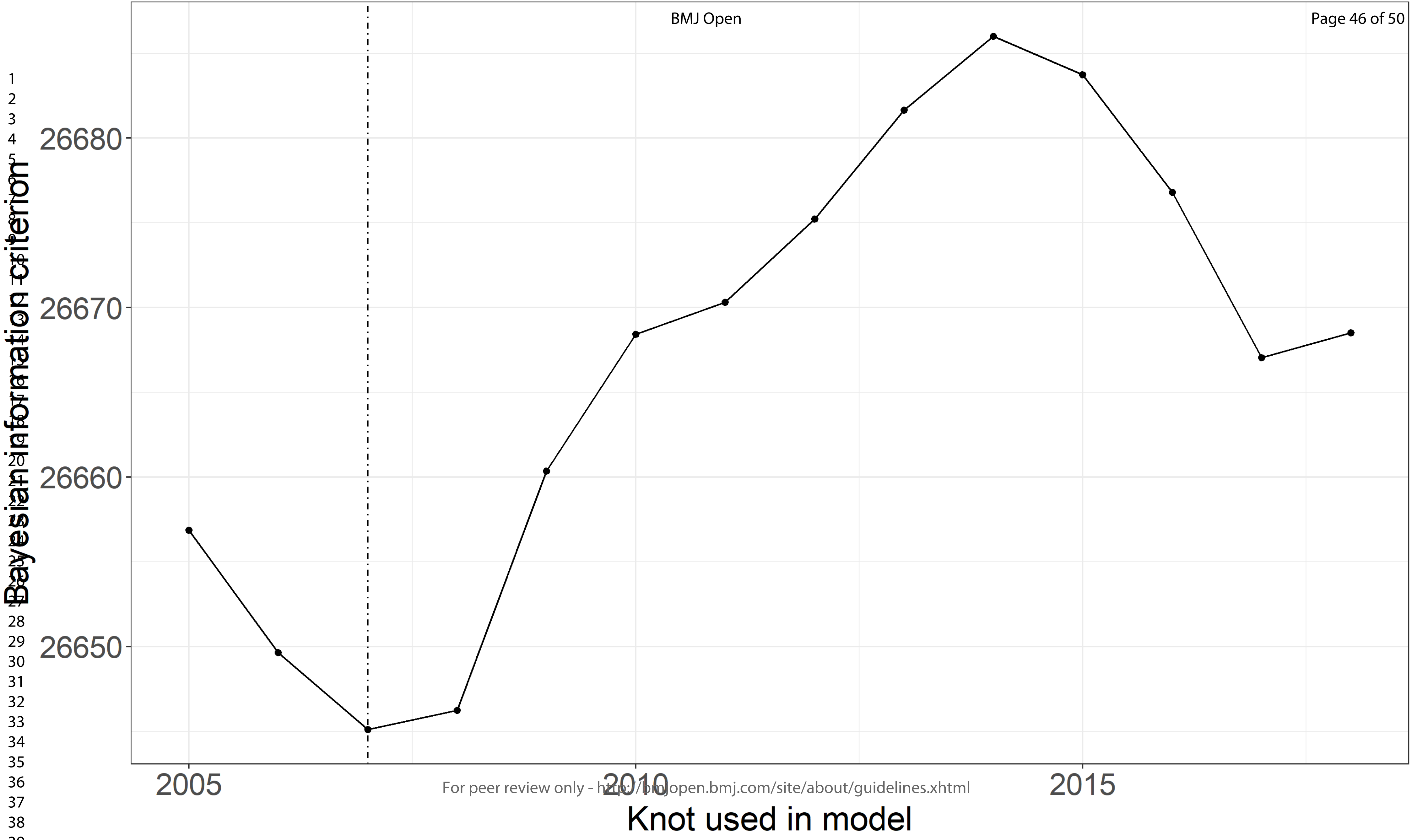
Bayesian information criterion
26680
26670
26660
26650
2005
2010
2015
Knot used in model

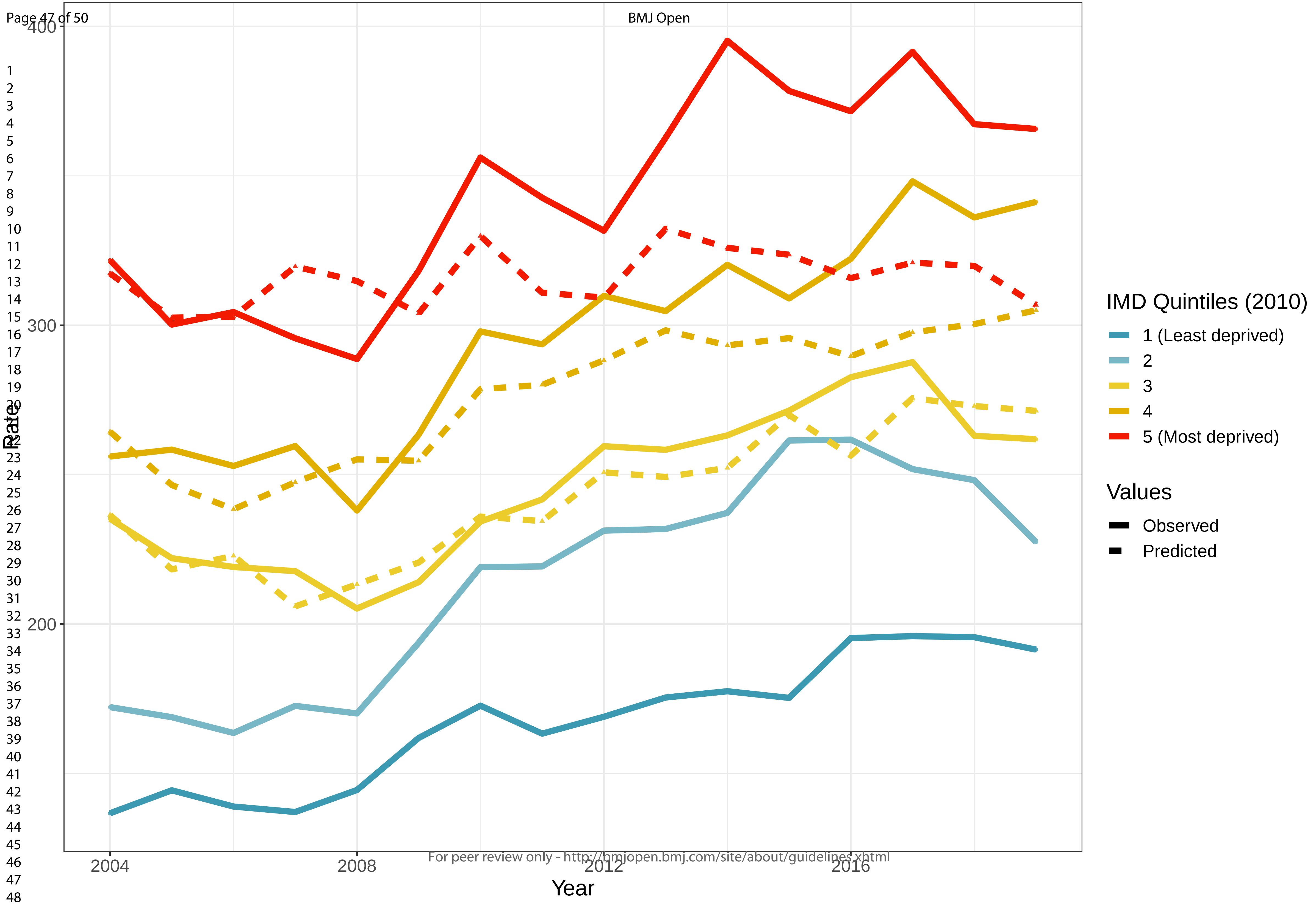
400
300
200
Rate
2004
2008
2012
2016
Year
IMD Quintiles (2010)
1 (Least deprived)
2
3
4
5 (Most deprived)
Values
Observed
Predicted

STROBE Statement—checklist of items that should be included in reports of observational studies

	Item No	Recommendation
**Title and abstract**	1	(*a*) Indicate the study's design with a commonly used term in the title or the abstract<br>Done. TITLE: "Trends in inequalities in looked after children in England 2004-2019: a local area ecological analysis"
		(*b*) Provide in the abstract an informative and balanced summary of what was done and what was found<br>Done. ABSTRACT: page 2
**Introduction**		
Background/rationale	2	Explain the scientific background and rationale for the investigation being reported.<br>Done. BACKGROUND: page 3
Objectives	3	State specific objectives, including any prespecified hypotheses<br>Done. BACKGROUND: page 2. "We investigated whether the rate of children becoming looked after between 2004 and 2019 increased more in deprived areas of the country – areas that, relative to more affluent areas, have higher levels of poverty – and the contribution of unemployment to these trends. We further quantify trends in inequalities in children experiencing other forms of child welfare intervention, in order to assess whether findings for CLA are consistent across child welfare outcomes."
**Methods**		
Study design	4	Present key elements of study design early in the paper<br>Done. METHODS: page 4
Setting	5	Describe the setting, locations, and relevant dates, including periods of recruitment, exposure, follow-up, and data collection.<br>Done. METHODS / DATA SOURCES AND MEASURES: pages 4-5
Participants	6	(*a*) *Cohort study*—Give the eligibility criteria, and the sources and methods of selection of participants. Describe methods of follow-up<br>NA: participants are the entire child population<br>*Case-control study*—Give the eligibility criteria, and the sources and methods of case ascertainment and control selection. Give the rationale for the choice of cases and controls<br>NA<br>*Cross-sectional study*—Give the eligibility criteria, and the sources and methods of selection of participants<br>NA
		(*b*) *Cohort study*—For matched studies, give matching criteria and number of exposed and unexposed<br>NA<br>*Case-control study*—For matched studies, give matching criteria and the number of controls per case<br>NA
Variables	7	Clearly define all outcomes, exposures, predictors, potential confounders, and effect modifiers. Give diagnostic criteria, if applicable.<br>Done. METHODS / DATA SOURCES AND MEASURES: pages 4-5

For peer review only - http://bmjopen.bmj.com/site/about/guidelines.xhtml

Data sources/ measurement	8*	For each variable of interest, give sources of data and details of methods of assessment (measurement). Describe comparability of assessment methods if there is more than one group. Done. METHODS / DATA SOURCES AND MEASURES: pages 4-5
Bias	9	Describe any efforts to address potential sources of bias Done. METHODS / DATA SOURCES AND MEASURES: pages 4-5. Use of routinely available data 2004-19 to assess descriptive trends.
Study size	10	Explain how the study size was arrived at. NA: the study size is the entire child population of England, analysed as aggregate data for 150 local authorities
Quantitative variables	11	Explain how quantitative variables were handled in the analyses. If applicable, describe which groupings were chosen and why. Done. METHODS/ / DATA SOURCES AND MEASURES: pages 4-5. "For our categorical measure of deprivation, LAs were assigned to quintiles according to their income deprivation score such that 20% of the 2008 child population was apportioned to each quintile."; "We also used a continuous measure of the income deprivation score, converted to a weighted rank by assigning a value from 0 to 1 based on the midpoint of the LA's range in the cumulative distribution. When using this value as a continuous exposure variable in the regression model, the estimated coefficient expresses the Slope Index of Inequality (SII)"
Statistical methods	12	(a) Describe all statistical methods, including those used to control for confounding. Done. METHODS / STATISTICAL ANALYSIS: page 7
		(*b*) Describe any methods used to examine subgroups and interactions Done. METHODS / STATISTICAL ANALYSIS: page 7 . "We included an interaction between the spline terms for the effect of year and deprivation to allow for potential differences in trend by SEC."
		(*c*) Explain how missing data were addressed Done. SUPPLEMENTARY MATERIALS / APPENDIX 3. "In the data, for reasons of confidentiality, numbers from one to five inclusive were supressed. For each missing value we therefore imputed a random integer in this range. There were only three cases of missing data, across two years, early in the implementation of the CIN census: age stratified data were not available for Havering and Newham in 2012, or Norfolk in 2013. Given the low degree of missingness, we performed complete case analyses."
		(*d*) *Cohort study*—If applicable, explain how loss to follow-up was addressed NA *Case-control study*—If applicable, explain how matching of cases and controls was addressed NA *Cross-sectional study*—If applicable, describe analytical methods taking account of sampling strategy NA
		(e) Describe any sensitivity analyses. NA
**Results**		
Participants	13*	(a) Report numbers of individuals at each stage of study—eg numbers potentially eligible, examined for eligibility, confirmed eligible, included in the study, completing follow-up, and

For peer review only - http://bmjopen.bmj.com/site/about/guidelines.xhtml

		analysed<br>NA
		(b) Give reasons for non-participation at each stage<br>NA
		(c) Consider use of a flow diagram<br>NA
Descriptive data	14*	(a) Give characteristics of study participants (eg demographic, clinical, social) and information on exposures and potential confounders<br>NA
		(b) Indicate number of participants with missing data for each variable of interest<br>Done. SUPPLEMENTARY MATERIALS / APPENDIX 3. "In the data, for reasons of confidentiality, numbers from one to five inclusive were supressed. For each missing value we therefore imputed a random integer in this range. There were only three cases of missing data, across two years, early in the implementation of the CIN census: age stratified data were not available for Havering and Newham in 2012, or Norfolk in 2013. Given the low degree of missingness, we performed complete case analyses."
		(c) *Cohort study*—Summarise follow-up time (eg, average and total amount)<br>NA
Outcome data	15*	*Cohort study*—Report numbers of outcome events or summary measures over time<br>NA
		Case-control study—Report numbers in each exposure category, or summary measures of exposure<br>NA
		Cross-sectional study—Report numbers of outcome events or summary measures<br>NA
Main results	16	(*a*) Give unadjusted estimates and, if applicable, confounder-adjusted estimates and their precision (eg, 95% confidence interval). Make clear which confounders were adjusted for and why they were included.<br>Done. RESULTS: pages 5-8. We have provided data on unadjusted trends in descriptive plots. In the models we have adjusted for unemployment.
		(*b*) Report category boundaries when continuous variables were categorized<br>NA
		(*c*) If relevant, consider translating estimates of relative risk into absolute risk for a meaningful time period<br>Done. RESULTS: pages 5-8. Absolute change in rates reported.
Other analyses	17	Report other analyses done—eg analyses of subgroups and interactions, and sensitivity analyses.<br>Done. SUPPLEMENTARY MATERIALS / APPENDICES 6-7. Age stratified analyses of crude CLA, CPP and CIN rates by LA deprivation quintile; Crude CPP rates by deprivation quintile, stratified by category of abuse
**Discussion**		
Key results	18	Summarise key results with reference to study objectives.<br>Done. DISCUSSION: page 9
Limitations	19	Discuss limitations of the study, taking into account sources of potential bias or imprecision. Discuss both direction and magnitude of any potential bias<br>Done. DISCUSSION: page 9
Interpretation	20	Give a cautious overall interpretation of results considering objectives, limitations,

For peer review only - http://bmjopen.bmj.com/site/about/guidelines.xhtml

		multiplicity of analyses, results from similar studies, and other relevant evidence.<br>Done. DISCUSSION: pages 9-11
Generalisability	21	Discuss the generalisability (external validity) of the study results.<br>Done. DISCUSSION / STRENGTHS AND LIMITATIONS: page 10
**Other information**		
Funding	22	Give the source of funding and the role of the funders for the present study and, if applicable, for the original study on which the present article is based<br>Done. FUNDING SOURCES: page 12.

*Give information separately for cases and controls in case-control studies and, if applicable, for exposed and unexposed groups in cohort and cross-sectional studies.

Note: An Explanation and Elaboration article discusses each checklist item and gives methodological background and published examples of transparent reporting. The STROBE checklist is best used in conjunction with this article (freely available on the Web sites of PLoS Medicine at http://www.plosmedicine.org/, Annals of Internal Medicine at http://www.annals.org/, and Epidemiology at http://www.epidem.com/). Information on the STROBE Initiative is available at www.strobe-statement.org.

For peer review only

For peer review only - http://bmjopen.bmj.com/site/about/guidelines.xhtml
